# Phenylbenzothiazole-Based Platinum(II) and Diplatinum(II) and (III) Complexes with Pyrazolate Groups: Optical Properties and Photocatalysis

**DOI:** 10.1021/acs.inorgchem.3c03532

**Published:** 2024-01-10

**Authors:** David Gómez de Segura, Andrea Corral-Zorzano, Eduardo Alcolea, M. Teresa Moreno, Elena Lalinde

**Affiliations:** Departamento de Química, Instituto de Investigación en Química (IQUR), Complejo Científico Tecnológico, Universidad de La Rioja, Madre de Dios 53, Logroño 26006, Spain

## Abstract

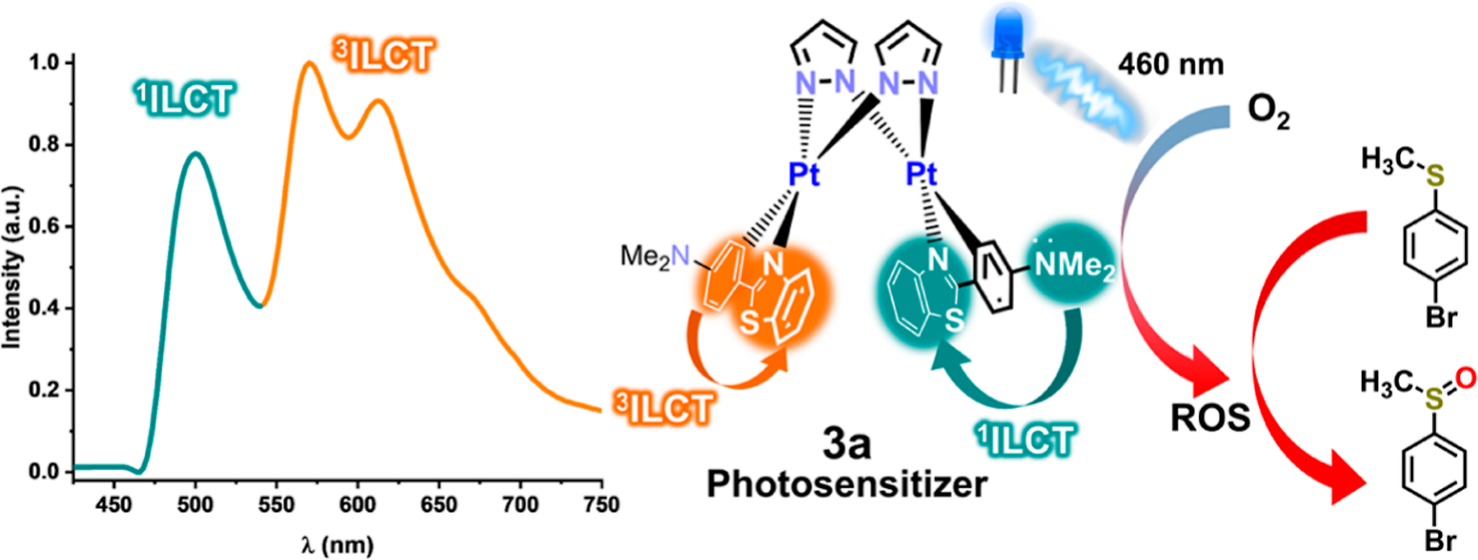

Based on 2-phenylbenzothiazole (pbt) and 2-(4-dimethylaminophenyl)benzothiazole (Me_2_N-pbt), mononuclear [Pt(pbt)(R′_2_-pzH)_2_]PF_6_ (R′_2_-pzH = pzH **1a**, 3,5-Me_2_pzH **1b**, 3,5-^i^Pr_2_pzH **1c**) and diplatinum (Pt^II^–Pt^II^) [Pt(pbt)(μ-R′_2_pz)]_2_ (R′_2_-pz = pz **2a**, 3,5-Me_2_pz **2b**, 3,5-^i^Pr_2_pz **2c**) and [Pt(Me_2_N-pbt)(μ-pz)]_2_ (**3a**) complexes have been prepared. In the presence of sunlight, **2a** and **3a** evolve, in CHCl_3_ solution, to form the Pt^III^–Pt^III^ complexes [Pt(R-pbt)(μ-pz)Cl]_2_ (R = H **4a**, NMe_2_**5a**). Experimental and computational studies reveal the negligible influence of the pyrazole or pyrazolate ligands on the optical properties of **1a**–**c** and **2a**,**b**, which exhibit a typical ^3^IL/^3^MLCT emission, whereas in **2c** the emission has some ^3^MMLCT contribution. **3a** displays unusual dual, fluorescence (^1^ILCT or ^1^MLCT/^1^LC), and phosphorescence (^3^ILCT) emissions depending on the excitation wavelength. The phosphorescence is lost in aerated solutions due to sensitization of ^3^O_2_ and formation of ^1^O_2_, whose determined quantum yield is also wavelength dependent. The phosphorescence can be reversibly photoinduced (365 nm, ∼ 15 min) in oxygenated THF and DMSO solutions. In **4a** and **5a**, the lowest electronic transitions (S_1_–S_3_) have mixed characters (LMMCT/LXCT/L’XCT **4a** and LMMCT/LXCT/ILCT **5a**) and they are weakly emissive in rigid media. The ^1^O_2_ generation property of complex **3a** is successfully used for the photooxidation of *p*-bromothioanisol showing its potential application toward photocatalysis.

## Abstract 2

We present a novel series of luminescent mononuclear (**1a–c)** and bimetallic (Pt^II^–Pt^II^) (**2a**−**c**, **3a**) and (Pt^III^–Pt^III^) (**4a**, **5a**) complexes based on phenylbenzothiazole (pbt, Me_2_N-pbt) cyclometalating chromophores and pyrazole (**1**) or pyrazolate-bridging ligands (**2**–**5**). Interestingly, the Pt^III^–Pt^III^ complexes (**4a**, **5a**) are unusually emissive in rigid media and **3a**, which displays a dual emission fluorescence (^1^ILCT or ^1^MLCT/^1^LC) and an extremely oxygen-sensitive ^3^ILCT phosphorescence, depending on the excitation wavelength, has been successfully used for the photooxidation of *p*-bromothioanisol.

## Introduction

Luminescent transition-metal compounds have attracted considerable attention owing to their wide range of applicability, such as emitters for light-emitting devices, optoelectronic materials, bioimagen probes, sensors, and photocatalysis.^1^ Among these complexes, cyclometalated platinum complexes are considered one of the most promising materials due to their rich and tunable excited-state properties, which can be fine-tuned by molecular design.^2^ Thus, mononuclear Pt^II^ complexes exhibit highly efficient triplet-state phosphorescence attributed to ^3^LC(^3^ππ), ^3^MLCT, or ^3^LC/^3^MLCT and ^3^LLCT/^3^MLCT mixtures, depending on the cyclometalating and ancillary ligands. A remarkable feature of these complexes is their propensity to develop, in the ground and/or upon excitation, noncovalent *intermolecular* platinum–platinum contacts through the interaction of the filled 5d_*z*^2^_ orbitals and ππ stacking interactions, particularly favored by the coordination of planar chelating aromatic ligands. These interactions are accompanied by simultaneous changes in the spectroscopic and optical properties, such as assembly-induced metal–metal–to ligand charge-transfer (MMLCT) transitions, which appear at lower energies than the corresponding monomers, and may be utilized to achieve single-doped white OLEDs^3^ or stimuli-responsive functional materials.^4^ Many of these systems have demonstrated to be solid-state low-red or near-infrared (NIR) emitters, with attractive applications in OLEDs^5^ or biological imaging.^6^

**Chart 1:**
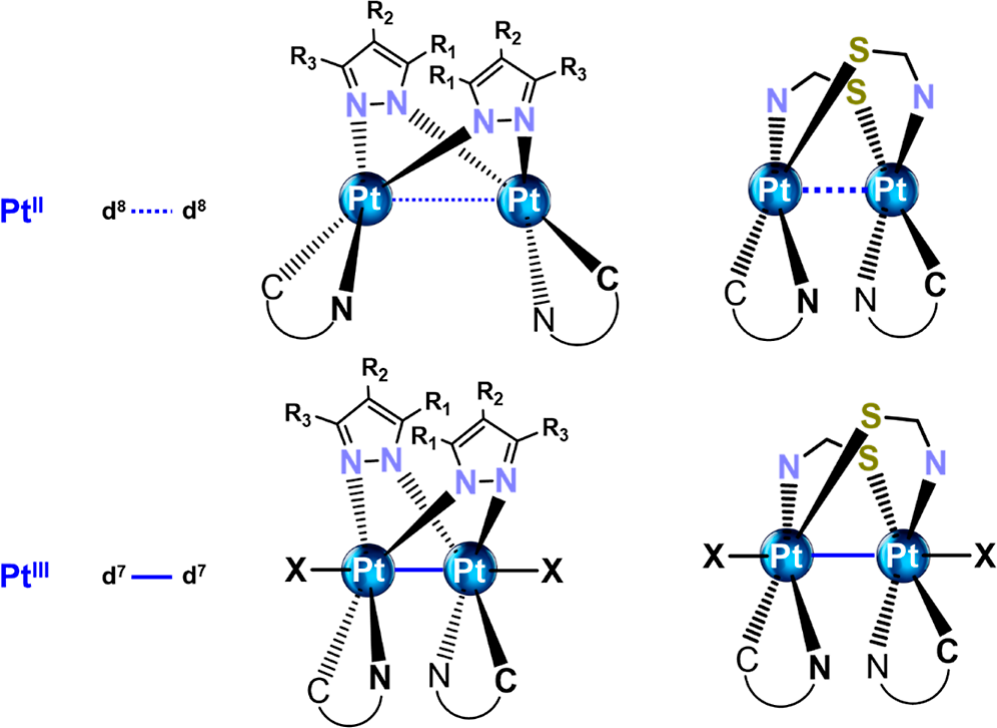
Schematic Drawings of Diplatinum(II) and (III) Complexes Bearing Pyrazolate and N^S Bridging Ligands.

Another successful approach to modulate the optical properties is through the design of new bimetallic platinum(II) complexes in which the *intramolecular* Pt···Pt distance can be synthetically manipulated, primarily by the nature and bulkiness of the bridging ligands and to a minor extent by the cyclometalating groups. In addition, in bimetallic complexes, the efficiency is usually notably increased, which has been attributed to the enhanced coupling between the T_1_ state and higher lying singlet states due to the incorporation of a second Pt center.^7^ Considerable interest has focused on two main categories, one featuring a *butterfly* shape and pyrazolate bridging ligands with relatively longer metal–metal separations and the other having a *half-lantern* shape and bridging thiolates with shorter intermetallic distances (Chart 1). Indeed, very efficient NIR phosphorescent OLED emitters have been reported using single-emissive binuclear *half-lantern* cycloplatinated complexes with various thiolates (pyridyl-thiolate, oxadiazole-thiolate, or benzo[*d*]thiazole-2-thiolate) as bridging ligands associated with a very strong ^3^MMLCT emission due to relatively short Pt···Pt distances.^5b,8^ In the *butterfly* shape, Pt^II^ complexes bearing pyrazolate bridging groups, the electronic structure mainly depends on the steric bulkiness of the substituent groups of the pyrazolate ligands, which modulates the Pt···Pt separation not only in the ground state but also in the excited state. When the Pt···Pt separation is relatively large, the low-lying transitions are due to mixed LC/MLCT excitations located on separated platinum units because, upon excitation, there is a local T_1_ minimum with a similar geometry to the S_0_ ground state. However, if the Pt···Pt separation are shorter, upon excitation, easy intersystem crossing (on a subpicosecond time scale) to the ^1,3^MMCT state takes place, leading to a T_1_-global minimum in which further contraction of Pt–Pt distance (∼0.2–0.3 Å) occurs, due to depopulation of the dσ*, characteristic of the ^3^MMLCT excited state.^9^ In these systems, the photoinduced molecular structural change that takes place upon excitation, strongly depends on the surrounding environment of the molecule and, as a consequence, access to both excited states occurs giving rise to dual emissions depending on the media and the concentration.^8a,10^ The dynamics and properties of the excited states of these binuclear complexes have been explored by ultrafast spectroscopic techniques (femtosecond absorption spectroscopy) and density functional theory (DFT) calculations in recent years.^9a,11^ The electronic characteristics of the chromophoric cyclometalating group also play a key role on the final electronic nature of these diplatinum complexes, but its effect has been comparatively less explored.^12^ In this field, despite the tremendous role that Pt···Pt *nonbonded* interactions play on the optical properties of cycloplatinated complexes, much less attention has been devoted to complexes featuring formal covalent Pt–Pt bonds (Chart 1).^13^ Indeed, in diplatinum cycloplatinated compounds, reports on emissive Pt^III^–Pt^III^ (d^7^–d^7^) derivatives are quite rare.^14^ In these complexes, the lowest lying excited state has usually a remarkable metal center character (dσ_M_–dσ*_M_), being therefore nonemissive.

Heterocycles featuring benzothiazole frameworks have been widely recognized as biologically active compounds^15^ and are also important fluorophores used to construct donor/acceptor systems displaying intramolecular charge-transfer properties.^16^ In this context, over the past few years, we have focused on the design and study of the optical and biological properties of cyclometalated Pt^II^, Pt^IV^, and Ir^III^ mononuclear complexes based on 2-phenylbenzothiazole (pbt) and 2-(4-dimethylaminophenyl)benzothiazole (Me_2_N-pbt) frameworks.^17^ Following our interest in these systems, here we report new families of mononuclear [Pt(pbt)(R′_2_-pzH)_2_]PF_6_ (R′_2_-pzH = pzH **1a**, 3,5-Me_2_pzH **1b**, 3,5-^i^Pr_2_pzH **1c**) and binuclear (Pt^II^–Pt^II^) [Pt(pbt)(μ-R′_2_pz)]_2_ (**2a**–**c**), [Pt(Me_2_N-pbt)(μ-pz)]_2_ (**3a**) and Pt^III^–Pt^III^ [Pt(R-pbt)(μ-pz)Cl]_2_ (R = H **4a**, NMe_2_**5a**) complexes based on phenylbenzothiazole (pbt) and 2-(4-dimethylaminophenyl)benzothiazole (Me_2_N-pbt) as cyclometalated groups and different pyrazole (**1**) or pyrazolate (**2**–**5**) as bridging ligands. Their photophysical properties, supported by theoretical calculations, are presented.

On the other hand, sulfoxides, in particular asymmetric sulfoxides, are nowadays widely used in organic synthesis, fine chemicals, medicine, pesticides,^18^ and, recently, have also emerged as efficient ligands in transition-metal catalysis.^19^ Common oxidants for sulfide oxidation to sulfoxides (H_2_O_2_, K_2_S_2_O_8_, or *m*-chloroperbenzoic acid) are usually not eco-friendly or cause serious environmental pollution. In recent years, photocatalytic oxidations have been successfully reported employing O_2_ as an environmentally friendly oxidant and different organic photosensitizers, such as Rose Bengal,^20^ Rivoflavin,^21^ or Bodipy.^22^ In this area, the employment of metal phosphorescent complexes as photosensitizers is rather less developed; although recently, the efficiency of several Ir^III^, Ru^II^ and Au^I^ systems have been demonstrated.^23^ To increase the knowledge on these systems, we sought to explore the utility of complex **3a**, which displayed strong oxygen sensitivity on its phosphorescent band, for the photocatalytic oxidation of sulfides using O_2_ as a green oxidant.

## Results and Discussion

### Synthesis and Characterization

#### Synthesis and Characterization of Cationic Bis-Pyrazole Pt^II^ Complexes

**Scheme 1:**
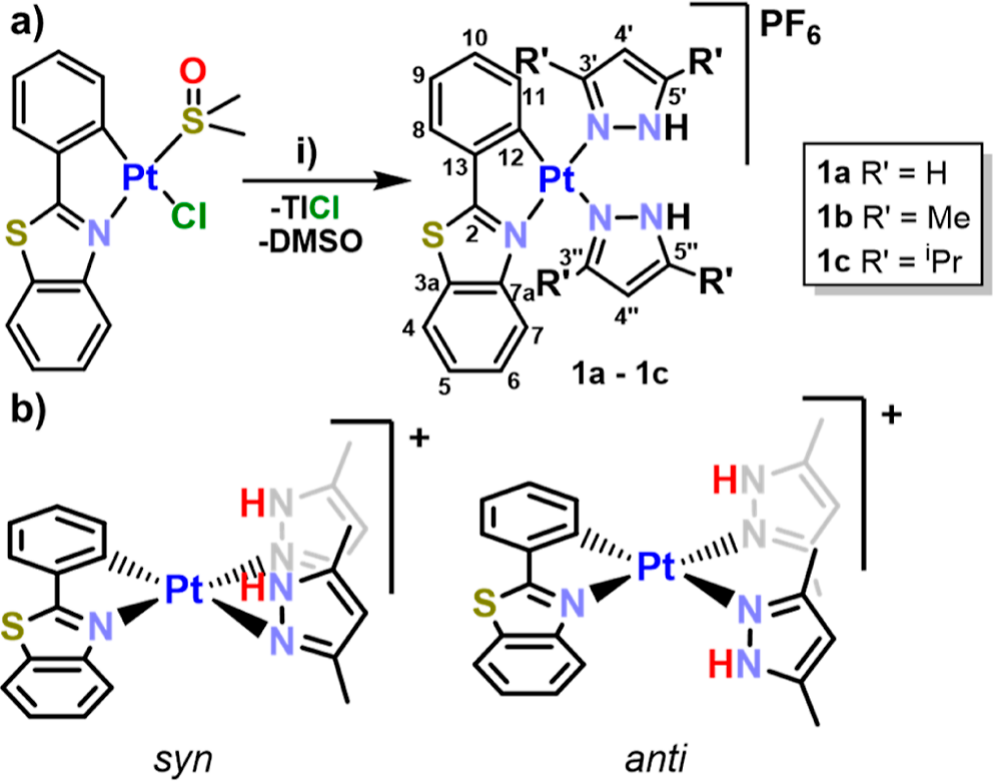
(a) Synthesis of Bis-pyrazole Complexes, (i) 3,5-R′_2_-pzH (1 equiv), TlPF_6_ (1 equiv), Acetone, 298 K, 6 h and (b) Schematic View of Atropisomers for **1b**

The reaction of the DMSO solvate [Pt(pbt)Cl(DMSO)]^17e^ with 1 equiv of TlPF_6_ and 2 equiv of the corresponding pyrazole ligand in acetone at room temperature gives rise to new bis-pyrazole complexes of the type [Pt(pbt)(R′_2_-pzH)_2_]PF_6_ (**1** R′_2_-pzH = pzH (**a**), 3,5-Me_2_pzH (**b**), and 3,5-^i^Pr_2_pzH (**c**)) (see Scheme 1 and Experimental Section). All attempts to generate related mononuclear complexes with the 2-(4-dimethylaminophenyl)benzothiazole as a cyclometalating ligand [Pt(Me_2_N-pbt)(R′_2_-pzH)_2_]PF_6_ starting from [Pt(Me_2_N-pbt)Cl(DMSO)] have been unsuccessful. In this case, the reactions evolve with formation of complex mixtures in which the chelating dimethylaminophenylbenzothiazole (Me_2_N-pbt) deprotonates the coordinated pyrazoles, giving rise to mixtures with the corresponding bridging pyrazolate bimetallic complexes.

After a workup, complexes **1a**–**c** were obtained as yellow solids in good yields (85–90%) and were fully characterized (see Figures S1–S3). They show strong IR absorptions due to the PF_6_ anion (ν 557, 841–845 cm^–1^) and broad bands at 3363–3384 cm^–1^, assigned to the tension frequency N–H of the pyrazole groups. The corresponding molecular peaks [Pt(pbt)(R′_2_pzH)_2_]^+^, found in the ESI(+) mass spectra of **1b** (*m*/*z* 597) and **1c** (*m*/*z* 709), and their molar conductivity (1:1 electrolytes) agree with the proposed stoichiometry. The presence of peaks related to the formation of [Pt_2_(pbt)_2_(pz)_2_] in **1a** (*m*/*z* 967 [Pt_2_(pbt)_2_(pz)_2_+Na]^+^, 945 [Pt_2_(pbt)_2_(pz)_2_ + H]^+^, and 877 [Pt_2_(pbt)_2_(pz)]^+^) suggests that this complex has a clear tendency to form the binuclear complex. The ^1^H and ^13^C{^1^H} NMR spectra in CD_3_COCD_3_ show one set of signals corresponding to the cyclometalated group and nonequivalent R′_2_pzH ligands. The assignment of the signals was made by ^1^H–^1^H (COSY and TOCSY) and ^1^H–^13^C (HSQC and HMBC) correlations. The more characteristic protons of the pbt ligand, H^7^ and H^11^, this last showing Pt coupling (^3^*J*_Pt–H_ ∼ 40 Hz), appear as doublets suffering an expected strong upfield shift [δ 6.18 (H^7^) and 6.26 (H^11^) **1a**, 6.26–6.19 (H^7^ and H^11^) **1b**, and 6.01 (H^7^) and 6.15 (H^11^) **1c**] compared to the precursor [Pt(pbt)Cl(DMSO)] [δ 9.62 (H^7^) and 8.42 (H^11^)], due to the anisotropic shield of the aromatic pyrazole ligand. Bis-pyrazole compound **1a** displays a broad signal with Pt satellites assigned to the H^3’^ (8.10 ppm, ^3^*J*_Pt–H_ 20.3 Hz), whereas the derivatives **1b**–**1c** show the corresponding signals of the alkyl substituents on the pyrazole co-ligands in the aliphatic region. Complexes **1a** and **1c** show two broad singlets at low frequencies due to the N–H protons of the pyrazole groups, whereas complex **1b** displays four signals indicating the presence of two conformational isomers in a *ca* 35:65 molar ratio. We note that in these complexes there are two possible relative orientations of the NH units of the pyrazole ligands in relation to the platinum coordination plane leading to a *syn/anti* isomerism (Scheme 1b). In complex **1b**, the interconversion between both conformers (atropisomers) seems to be slower than the NMR time scale. The presence of both conformers is also apparent in the H^4′,4″^ protons (δ 6.40–6.28 range, 2H) and methyl resonances (2.47–2.35 ppm, 2H) of the nonequivalent 3,5-dimethylpyrazole ligands and in the corresponding ^13^C{^1^H} NMR signals (See Figure S2). Finally, **1a**–**1c** complexes display the expected doublet (∼−72.6 ppm, ^1^*J*_F–P_ 706 Hz) and septuplet (∼−145 ppm) in the ^31^P{^1^H} and ^19^F{^1^H} NMR spectra, respectively, corresponding to the PF_6_ counteranion.

**Figure 1:**
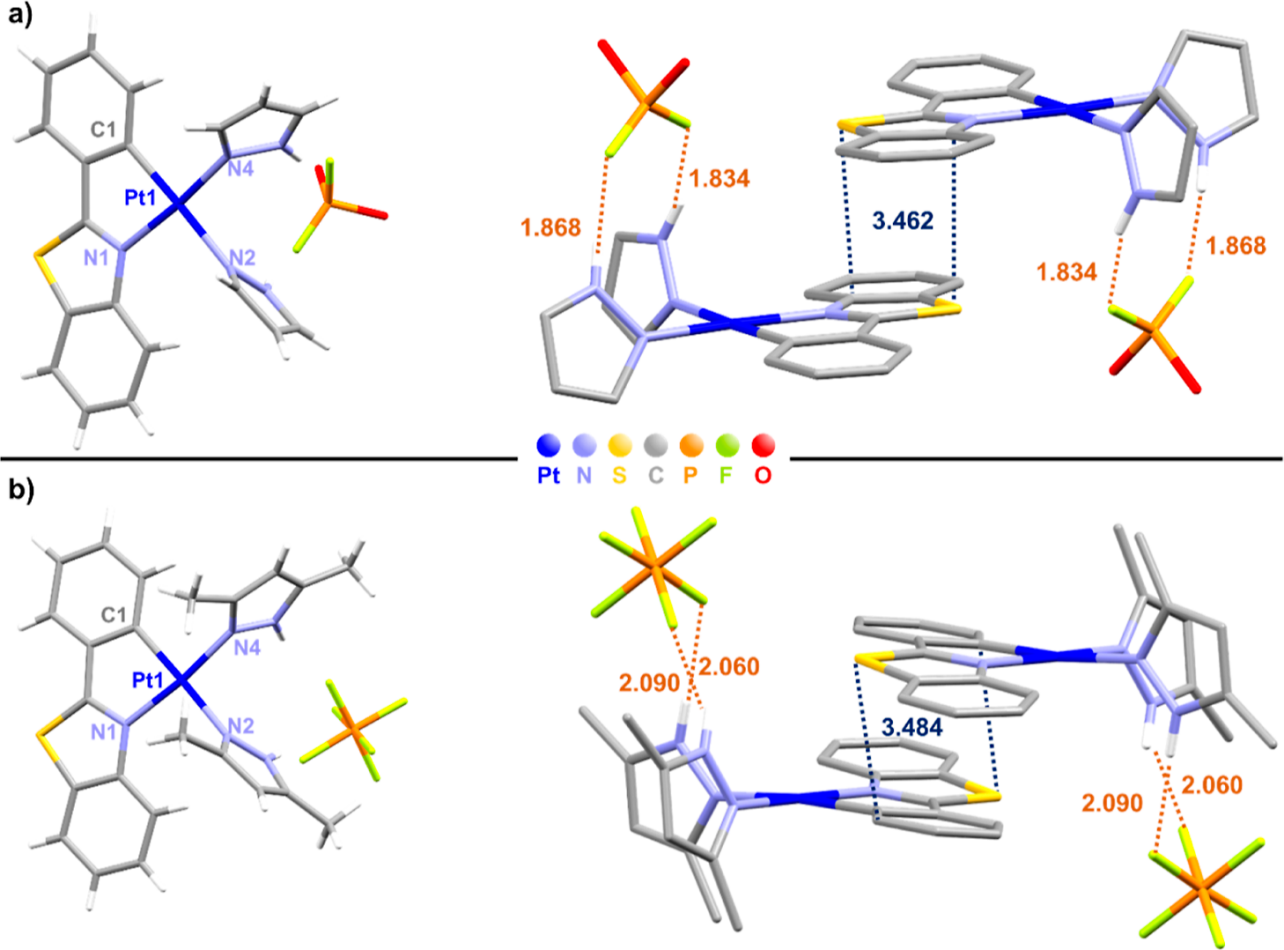
Molecular structure and crystal stacking of complexes **1a·PO**_**2**_**F**_**2**_ (a) and **1b** (b).

Suitable crystals for complexes **1a** and **1b** have been obtained by slow diffusion of *n*-hexane through a solution of the corresponding compound in acetone at low temperatures. In the case of **1a**, the X-ray diffraction study (Tables 1, S1 and S2) revealed that this complex crystallized with the PO_2_F_2_ anion generated by the partial hydrolysis of the PF_6_^–^ group (**1a·PO**_**2**_**F**_**2**_). Hydrolysis of the PF_6_^–^ anion has been previously observed.^17f^ A view of the molecular structures with the selected bond lengths and angles and a vision of the interaction of the cation and the anions is presented in Figure 1. Both cations showed the expected chelate C^N coordination of the pbt ligand and the *cis* arrangement of the two R′_2_-pzH ligands. The bite angle of the pbt ligand [80.4(9)° **1a·PO**_**2**_**F**_**2**_, 80.8(4)° **1b**] and the Pt–C(1) [2.01(2) **1a·PO**_**2**_**F**_**2**_, 2.007(10) Å **1b**] distance (Table 1) are within the expected values for this type of complexes.^24^ The Pt–N(2) distance [2.08(2) **1a·PO**_**2**_**F**_**2**_, 2.117(9) Å **1b**] *trans* to the C-metalated is longer than the Pt–N(4) distance [1.960(18) **1a·PO**_**2**_**F**_**2**_, 2.002(9) Å **1b**] (Table 1), reflecting the high *trans* influence of the metalated carbon. The phenyl benzothiazole ligand is almost coplanar with respect to the Pt coordination plane (deviations 2.60° **1a·PO**_**2**_**F**_**2**_, 7.56° **1b**). In the crystal, the pyrazole ligands display a *syn* orientation, which is stabilized by the occurrence of short hydrogen bonding interactions between the NH protons and the fluorine atoms of the counteranion. The F···H distances are shorter in **1a·PO**_**2**_**F**_**2**_ than in **1b** (1.868, 1.834 Å **1a·PO**_**2**_**F**_**2**_ vs 2.090, 2.060 Å **1b**) and within the range reported for F···H bonding interactions.^25^ Both complexes form dimers through moderate intermolecular π···π (pbt···pbt, 3.462 **1a·PO**_**2**_**F**_**2**_ and 3.484 Å **1b**) interactions (Figure 1).

**Table 1:** Selected Bond Distances (Å) and Angles (deg) of **1a·PO**_**2**_**F**_**2**_, **1b**, **2a**, **3a·THF**, **4a**, and **5a·0.5CH**_**2**_**Cl**_**2**_

parameter	**1a**·PO_2_F_2_	**1b**	**2a**	**3a**·THF	**4a**	**5a**·0.5CH_2_Cl_2_
Pt-C_C^N_	2.01(2)	2.007(10)	2.001(3)	2.001(6)	2.013(2)	2.012(6)
			1.998(3)	1.993(6)		2.012(7)
Pt-N_C^N_	2.042(16)	2.038(8)	2.036(2)	2.043(5)	2.052(2)	2.055(5)
			2.033(2)	2.039(5)		2.049(6)
Pt-N_pyr(*trans*-C)_	2.08(2)	2.117(9)	2.099(2)	2.103(5)	2.118(2)	2.146(5)
			2.090(2)	2.105(5)		2.137(6)
Pt-N_pyr(*trans*-N)_	1.960(18)	2.002(9)	1.989(2)	2.004(5)	2.0038(19)	2.001(6)
			2.000(2)	1.998(5)		2.003(6)
Pt-Pt			3.344	3.1740(4)	2.58972(19)	
Pt-Cl					2.4177(6)	2.4304(15)
						2.4112(19)
C_C^N_-Pt- N_pyr_	94.9(9)	92.6(4)	95.80(10)	95.0(2)	93.73(9)	91.4(2)
			95.80(11)	95.0(2)		92.2(3)
C_C^N_-Pt- N_C^N_	80.4(9)	80.8(4)	80.76(10)	81.6(2)	80.83(9)	80.9(2)
			80.92(11)	81.1(2)		80.8(3)
N_C^N_-Pt- N_pyr_	97.1(8)	99.2(3)	99.50(9)	98.7(2)	99.73(8)	102.3(2)
			98.42(9)	99.0(2)		100.8(2)
N_pyr_-Pt- N_pyr_	87.7(8)	87.45(3)	84.16(9)	84.8(2)	84.32(8)	84.0(2)
			84.72(9)	85.0(2)		84.3(2)
Cl-Pt-Pt					162.894(14)	165.71(6)
						166.71(6)

#### Synthesis and Characterization of Bis(pyrazolate) Diplatinum Complexes

**Scheme 2:**
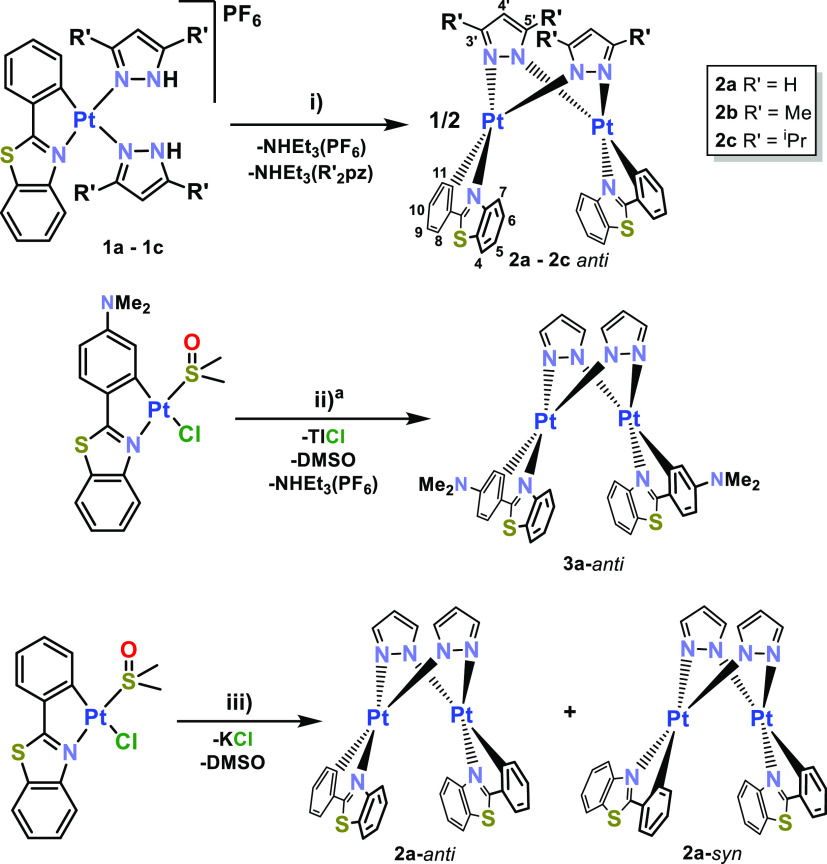
Synthesis and Conditions for the Preparation of Pt_2_^II^ Derivatives; (i) NEt_3_ (exc.), Acetone, 8 h; (ii) Hpz (1 equiv), TlPF_6_ (1 equiv), NEt_3_ (exc.), Acetone, 6 h; and (iii) Hpz (1 equiv), KOH (exc.), EtOH/Acetone, 24 h The isomer **3a**-*anti* was also obtained using via (iii).

The binuclear pbt–platinum complexes with pyrazolate bridging ligands [Pt(pbt)(μ-R′_2_pz)]_2_ (R′_2_-pz = pz **2a**, 3,5-Me_2_pz **2b**, 3,5-^i^Pr_2_pz **2c**) were prepared by the reaction between the corresponding bis-pyrazole [Pt(pbt)(R′_2_-pzH)_2_]PF_6_ (**1a**–**1c**) with excess of NEt_3_ at room temperature (Scheme 2i). The related 2-(4-dimethylaminophenyl)benzothiazole derivative [Pt(Me_2_N-pbt)(μ-pz)]_2_**3a** was also prepared by reaction of [Pt(Me_2_N-pbt)Cl(DMSO)] with 1 equiv of Hpz, TlPF_6_, and NEt_3_ (see Scheme 2ii) or by using Hpz (1 equiv) and excess of KOH (see the Supporting Information for details). Following these pathways, the binuclear complexes were selectively obtained as the *anti*-isomers. Complex **2a** was also prepared starting from the mononuclear complex [Pt(pbt)Cl(DMSO)], by using Hpz (1 equiv) and excess of KOH (Scheme 2iii). However, in this case, the reaction evolves with formation of a mixture of the isomers **2a**-*anti* and **2a**-*syn* in relation ∼5:1, as assessed by ^1^H NMR spectroscopy.

**Figure 2:**
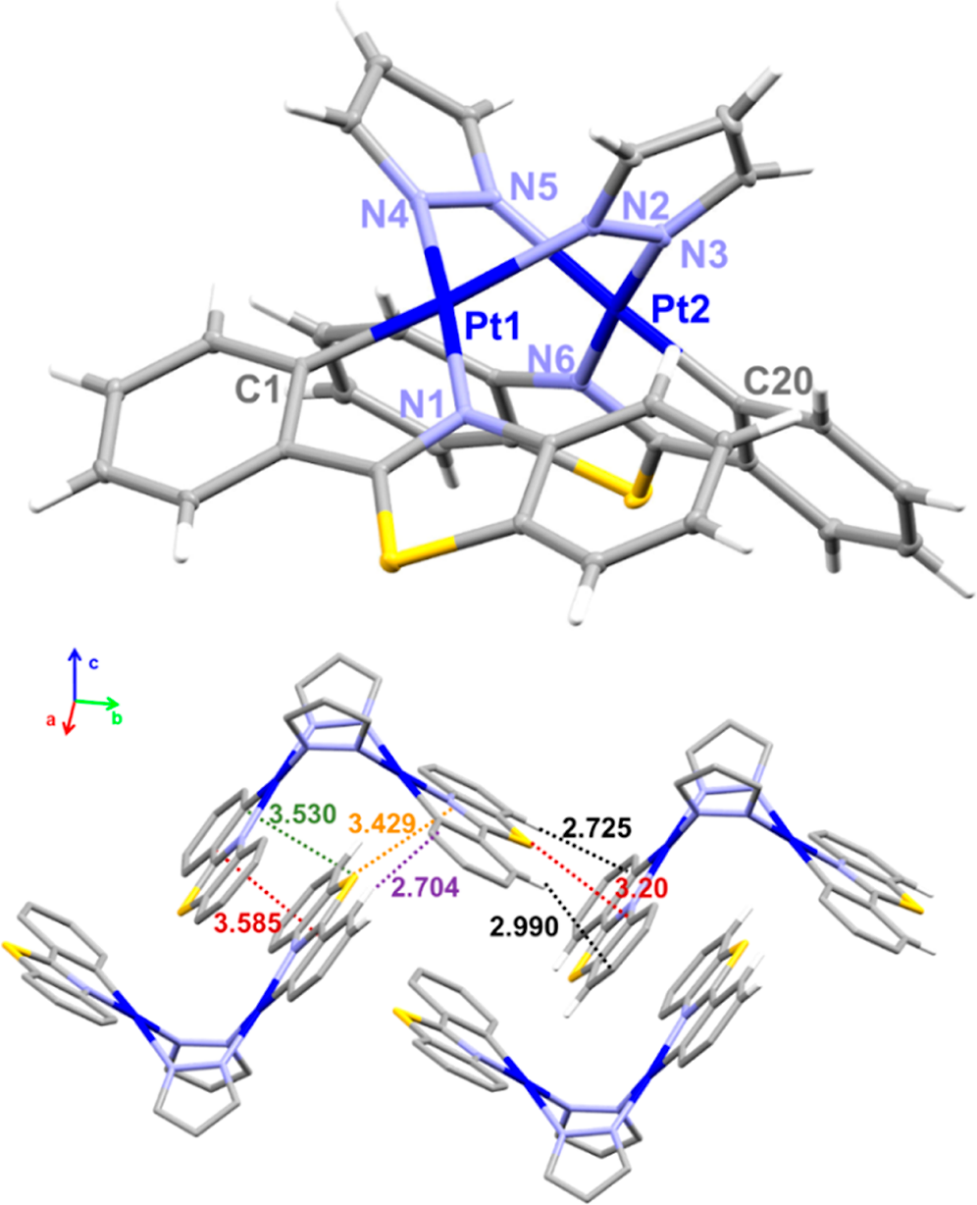
Molecular structure and crystal packing of complex **2a**.

The bimetallic nature of the complexes was supported by ESI(+) or MALDI(+)-MS analysis, as they show as the parent peak, the corresponding one due to [M + H]^+^ (*m*/*z* 945 **2a**, 1000 **2b**, 1112 **2c**, and 1030 **3a**) (Section S3 and Experimental Section in the Supporting Information). Unfortunately, only complexes **2a** and **3a** are soluble enough in THF-*d*^8^ to characterize them by ^1^H NMR spectra. The *anti-*isomers (*anti-***2a** and **3a**) exhibit the presence of only one set of pbt ligands together with one set of pyrazolate groups. In both complexes, the most deshielded signal corresponds to the H^7^ of the pbt, which appear as a doublet (δ 7.89 **2a**-*anti*, 7.74 **3a**), whereas the most shielded is attributed to the H^4’^ of bridging pyrazolate (δ 6.34 **2a**-*anti*, 6.3 **3a**) (Supporting Information, Section S2). In the *anti/syn*-**2a** mixture, the most distinct signal of the type of isomer corresponds to the H^4’^ of the pyrazolate, which are equivalent for the *anti*-**2a** isomer (δ 6.34) and inequivalent in the *syn*-**2a** (δ 6.48; 6.32) (Figure S4b). Suitable yellow crystals for X-ray studies of *anti*-**2a** and **3a·THF** were obtained from slow evaporation of a THF solution. Their molecular structures are depicted in Figures 2 and S11, and the corresponding structural bonding details are provided in Tables 1 and S2. The structures confirm the *anti*-arrangement of the Pt(R-pbt) units bridged by the two pyrazolate ligands, in coherence with the NMR spectra. Both Pt^II^ complexes display the typical butterfly-like structure with a *C*_2_ symmetry. The intermetallic distances [3.344 **2a** and 3.1740(4) Å **3a**, Table 1] are shorter than the sum of the van der Waals radii of the two Pt (3.5 Å) and comparable to those found in related μ-pyrazolate diplatinum complexes (2.834–3.486 Å).^12,26^ It has been previously shown that in this type of complexes, both the Pt···Pt distance and the tilt angle decrease due to the increasing demanding of the substituents of the bridging pyrazolate ligand (3- and 5-positions).^26d^ Also the bulkiness of the cyclometalating group affects to the proximity of the platinum fragments in these type of binuclear complexes.^27^ Surprisingly, despite the presence of bulky NMe_2_ groups in **3a**, the Pt···Pt is shorter and the angle between platinum planes is smaller than in **2a** (84.23° for **2a** and 73.50° for **3a**), indicating stronger interactions between the platinum fragments, a fact that might be attributed to the coplanarity between the NMe_2_ and the bt unit. On the basis of previous results, it is suggested that the Pt···Pt separation could likely be shorter in complex **2b** (R’ = Me) and, particularly, in **2c** featuring R’ = Pr^i^ groups, what agrees with their intense orange colors. The Pt–C and Pt–N bond distances around the Pt centers are similar to those found in related complexes. In particular, the Pt–N_pz_ distance *trans* to the C_R-pbt_ [2.099(2) **2a** and 2.103(5) Å **3a**] is longer than that of *trans* to the N of the R-pbt group [2.000(2) **2a** and 2.004(5) Å **3a**] (Table 1), in coherence with the high *trans* influence of the metalated carbon. The extended structure of both compounds shows weak π···π interactions between cyclometalated groups of different molecules giving rise to dimers (3.585–3.641 Å) supported by secondary *intermolecular* sulfur···carbon (S_R-pbt_···C_R-pbt_ ∼ 3.43 Å) and C–H···π (2.704–2.971 Å) interactions. These dimers additionally stack giving rise to a 1D infinite arrangement supported by C–H···π (2.778–2.836 Å) and (S_R-pbt_···C_R-pbt_ ∼ 3.20 Å) (Figures 2 and S11).

**Scheme 3:**
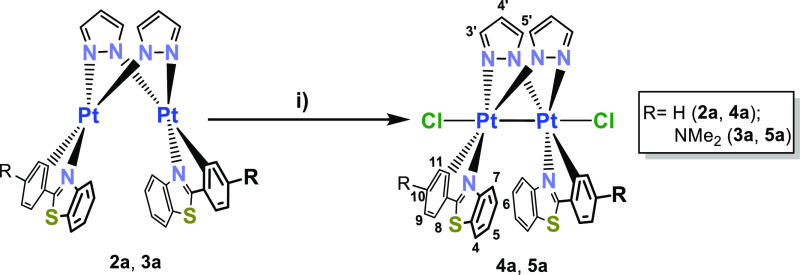
Synthesis and Conditions of Binuclear Bis-pyrazolate Pt_2_^III^ Complexes; (i) CHCl_3_, Sunlight, 298 K, 24 h or PhICl_2_, CH_2_Cl_2_, 0 °C, 8 h

We observed that complexes [Pt(R-pbt)(μ-pz)]_2_ (R = H **2a**, Me_2_N **3a**) are stable in CHCl_3_ solution in the dark, but in the presence of sunlight they evolve slowly (∼24 h) to form the metal–metal-bonded Pt^III^–Pt^III^ complexes [Pt(R-pbt)(μ-pz)Cl]_2_ (**4a**, **5a**), which precipitate in the mixture in a *ca* yield of 60%. This type of two center-two electron oxidation reactions have been previously studied and depend on many factors involving either a radical-like mechanism (with the O_2_ acting as a radical R* trap) and/or a thermally or photochemically activated processes.^26h,28^ Recent studies on pyrazolate- and thiolate-bridged diplatinum complexes with very short Pt···Pt distances indicate that the ^1^MMLCT excited states easily triggers the photooxidation of these complexes with CHCl_3_.^10b^ As expected, the complexes were also obtained by reacting **2a** and **3a** with iodobenzenedichloride (PhICl_2_) in CH_2_Cl_2_ at 0 °C, being precipitated and separated of the mixture in a 59–73% yield (Scheme 3). Complexes **4a** and **5a** have been characterized by mass spectrometry and NMR spectroscopy (Experimental Section and Sections S2 and S3 in the Supporting Information) and their structures confirmed by X-ray. The formation of the oxidized species is evident by the presence of peaks due to [M-Cl]^+^ (*m*/*z* 979 **4a**, 1065 **5a**) as parent peaks in their mass spectra.

**Figure 3:**
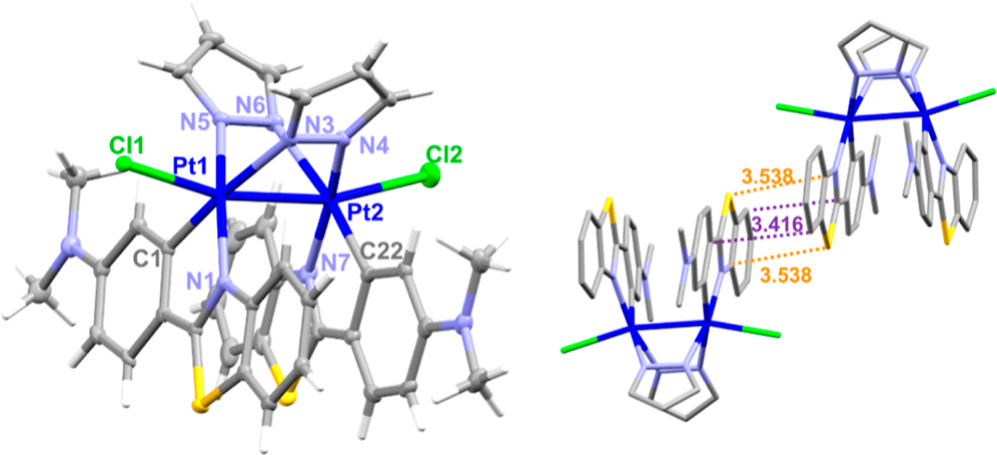
Molecular structure and crystal packing of **5a·0.5CH**_**2**_**Cl**_**2**_.

The ^1^H NMR spectra of **4a** and **5a** display only one set of signals for the R-pbt groups and for the pyrazolate ligands, indicating an *anti*-arrangement of the cyclometalated group. The *ortho* protons to the cyclometalated ligand, H^11^, are notably shielded and the three-bond platinum-coupling constant (^3^*J*_Pt–H_ 31.1 **4a**, 35.2 Hz **5a**) is smaller than in complex **1a** (43.3 Hz), in agreement with the increased oxidation state. The molecular structures of complexes **4a** and **5a·0.5CH**_**2**_**Cl**_**2**_ were determined by single-crystal X-ray diffraction (Table 1 and Figures 3 and S12). The Pt^III^–Pt^III^ complexes retain the boat-like structure of the Pt_2_N_4_ core and the *anti*-arrangement of the platinum(C^N) fragments. The interplanar angles between the platinum coordination planes are notably reduced with respect to those observed in Pt^II^–Pt^II^ precursors (35.68° **4a** and 34.30° **5a** vs 84.23° **2a** and 73.50° **3a**) due to the formation of the Pt–Pt bond. Each Pt^III^ center shows a distorted octahedral coordination environment with axial positions occupied by chloride atoms and the other Pt^III^ center, with angles Cl–Pt–Pt of 162.894(14)° for **4a** and 165.71(6)° and 166.71(6)° for **5a** (Table 1), similar to those reported for related complexes.^12,26h^ As expected, the formation of a Pt–Pt formal bond produces a shortening in the Pt–Pt distance with respect to complexes **2a** and **3a** [2.5897(2) **4a** 2.5776(3) Å **5a** vs 3.344 **2a**, 3.1740(4) Å **3a**, Table 1]. The molecular packing of these complexes shows extended π···π interactions through the R-pbt groups with distances of 3.458–3.544 Å in **4a** and 3.416–3.538 Å for **5a**, forming 1D chains (Figures 3 and S12).

### Optical Properties

#### Absorption Properties and TD-DFT Calculations

**Figure 4:**
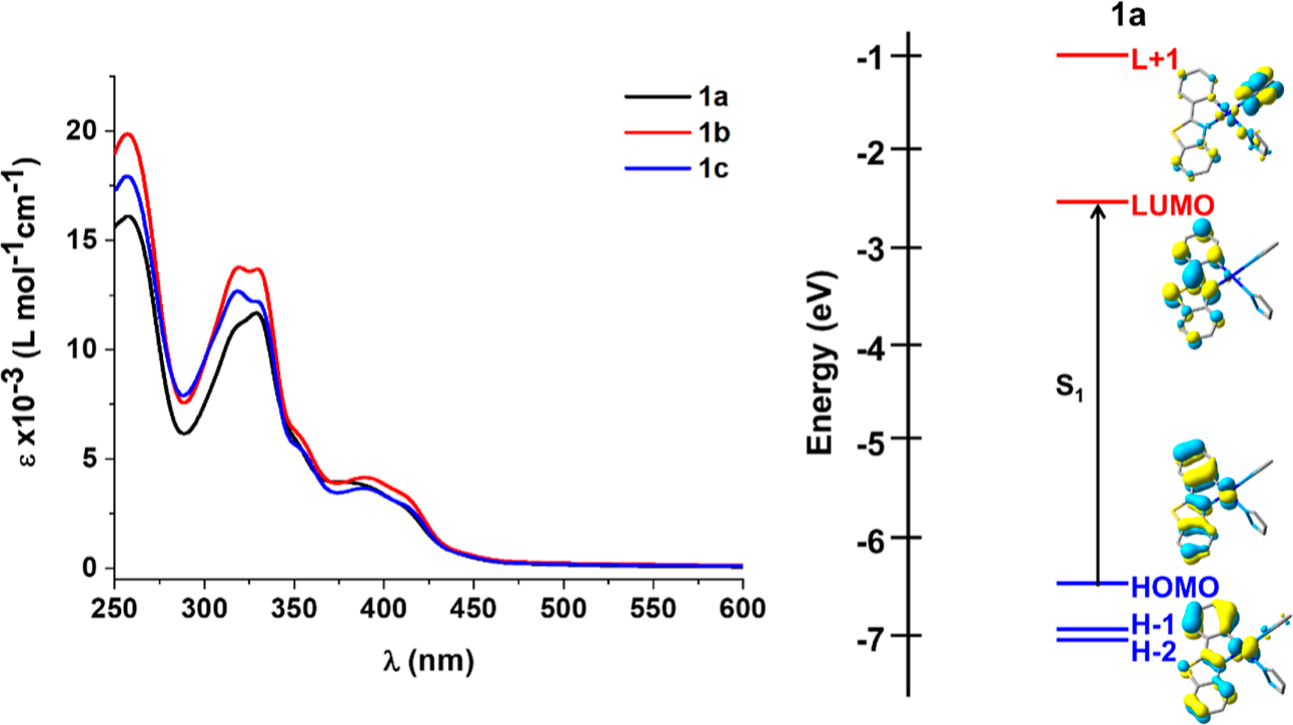
UV–vis absorption spectra of mononuclear complexes **1** and schematic representation of selected orbitals frontiers and transitions for **1a**.

The UV–vis spectra of bis(pyrazole) complexes **1a**–**1c** in THF (5 × 10^–5^ M) solution and in the solid state (Table S3, Figures 4 and S13a) are rather similar, indicating a negligible influence of the pyrazole groups. In solution, they show one intense absorption at ca. 260 nm, attributed to spin-allowed π–π* intraligand (^1^IL, pbt) transitions. They exhibit an additional band in the 300–370 nm range and a less intense feature in the low-energy region at 385–420 nm (ε ∼ 10^3^ L·mol^–1^·cm^–1^). On the basis of theoretical calculations carried out in complex **1a** (Figure 4 and Section S5, Supporting Information), which indicate that the calculated low energy transition (S_1_ 382 nm) is mainly contributed by the HOMO (80% pbt, 20% Pt) to LUMO (93% pbt, 6% Pt) excitation, the low-energy feature is mainly attributed to an intraligand ^1^IL (pbt) with some ^1^MLCT. The band in the range of 300–370 nm has also a mixed ^1^MLCT/^1^IL character. The Hpz orbitals contribute from the LUMO+1 (63%) and they are involved in the S_6_/S_8_ transitions with very low oscillator strength (Table S4 and Figure S15).

**Figure 5:**
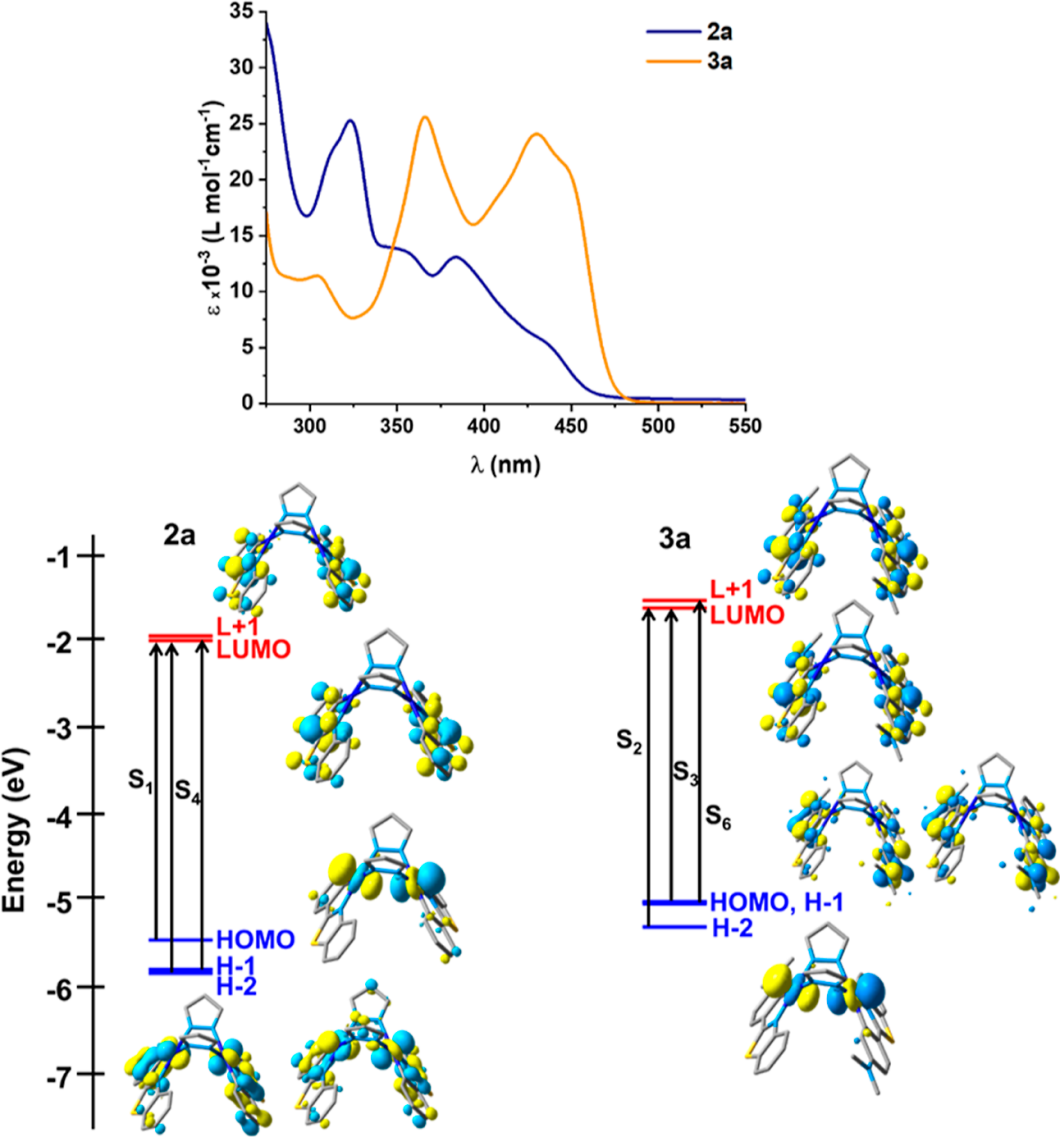
UV–vis absorption spectra of mononuclear complexes **2a** and **3a** in THF (5 × 10^–5^ M) and an schematic representation of their frontier orbitals and selected excitations.

As noted, the binuclear Pt^II^–Pt^II^ complexes **2b** and **2c** are insoluble in common solvents. Therefore, only the absorption spectra of the pyrazolate bridging complexes **2a** and **3a** could be recorded in THF solution (Figure 5). In both compounds, the band at λ < 350 nm are ascribed to π–π* intraligand (^1^IL, pbt) and ligand-to-ligand (R_2_pz to pbt) transitions. In **2a**, the bands between 350 and 410 nm are ascribed to mixed ^1^IL/^1^MLCT and the low energy feature at *ca*. 440 nm to ^1^MMLCT transitions, as supported by calculations (see Table S4). In the Me_2_N-pbt **3a**, the bands are more intense indicating a stronger intraligand charge-transfer contribution (NMe_2_ to bt) and are slightly red-shifted (**3a** 430, 450 nm ε ∼ 20 × 10^3^ vs **2a** 384, 440 nm ε 4.78 × 10^3^). As seen in Figure 5, which shows the frontier orbitals for both complexes, in both, the target LUMO and L+1 spread on the cyclometalated ligands. However, whereas in **2a**, the highest orbital HOMO has a σ* (5d_*z*^2^_–5d_*z*^2^_) character and is located on the two platinum atoms; in **3a**, a similar orbital is the H-2. In **3a**, the HOMO and HOMO–1 are destabilized in relation to **2a** and are located on the Me_2_N-pbt. For **2a**, the calculated S_1_ (452 mn) and S_2_ (439 nm) have ^1^MMLCT characters, whereas for **3a** the most intense calculated absorptions are S_2_ (423 mn) and S_3_ (415 nm) and have mixed ^1^ILCT/^1^MMLCT. It is interesting to note that in both complexes the calculated transitions with remarkable metal platinum contribution and mixed ^1^MLCT/^1^ILCT character appear at higher energy (S_4_ 397 nm **2a**; S_7_ 351 nm **3a**). The solid-state absorption spectra of **2a**–**2c** have been also recorded. The main lowest-energy band ranges from 438 (**2a**), 455 (**2b**) to 503 nm (**2c**), with extending tails, in agreement with their color (Table S3 and Figures S13, S14), what are ascribed to mixed ^1^IL/^1^MMLCT, with higher contribution of this latter on going from **2a** to **2c**, likely due to a lower Pt–Pt distance by increasing the steric bulk of the substituents. Low intense features in the tails (>480 nm **2a**, **2b** or 540 nm, **2c**) are observed, which are probably of spin-forbidden nature.

**Figure 6:**
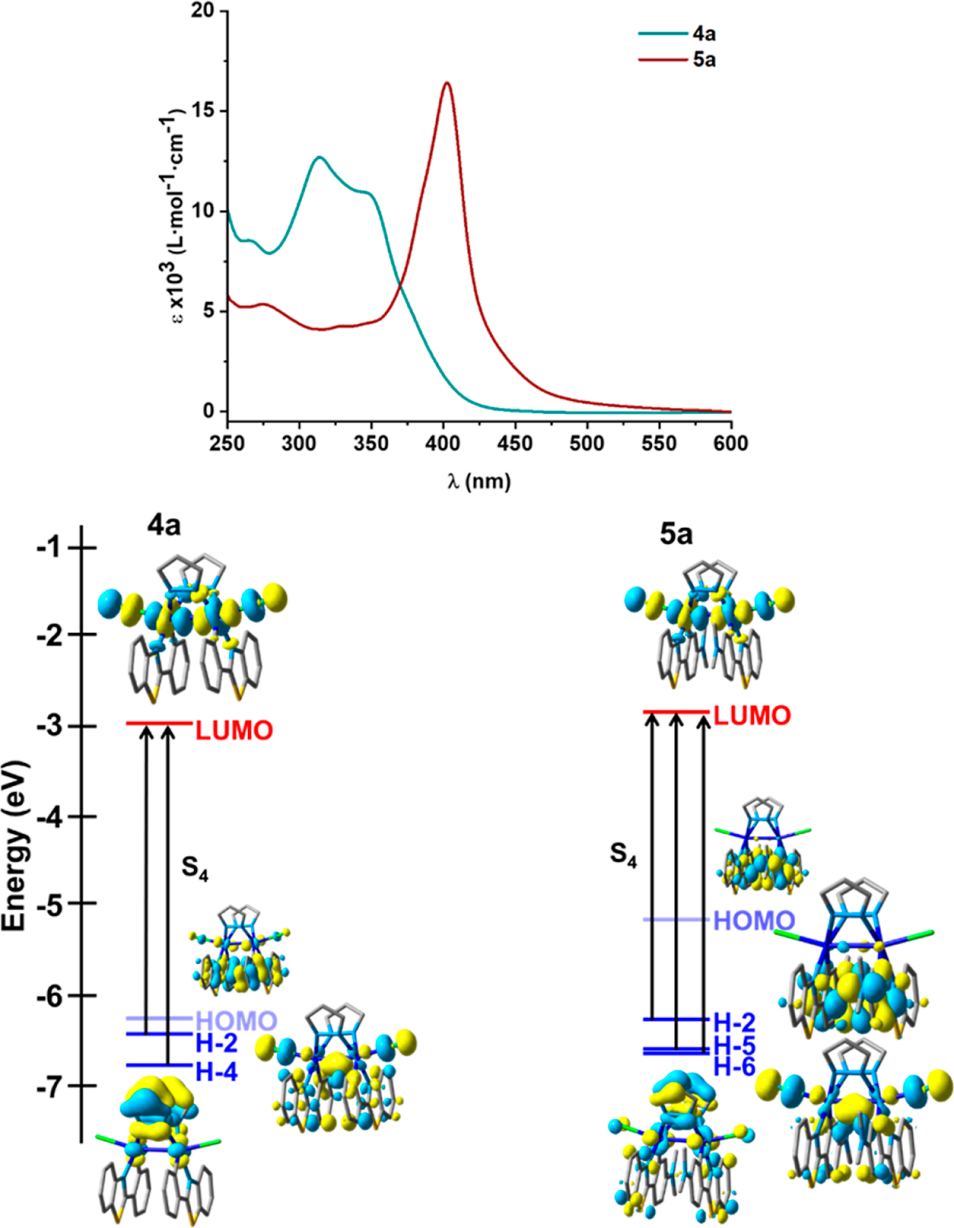
UV–vis absorption spectra in THF solution (5 × 10^–5^ M) of **4a** and **5a** and an schematic representation of their frontier orbitals and selected excitations.

The absorption spectra of Pt^III^–Pt^III^**4a** and **5a** are included in Figure 6. The most significant feature in relation to complexes **2a** and **3a** is the notable hypsochromic shift in the low energy region. Thus, **4a** displays a moderately intense absorption low energy band at 348 nm with a shoulder at 379 nm and **5a** a band at 402 with a shoulder at 444 nm, clearly blue-shifted in relation to the corresponding Pt^II^–Pt^II^ (440 **2a**, 430 nm **3a**), reflecting to the oxidation of the platinum centers and the notable change in the frontier orbitals. DFT and time-dependent DFT (TD-DFT) calculations were performed with the Gaussian 16 program package to explore the orbital frontiers and the nature of the excited states and transitions (Tables S4–S7 and Figure S16). Figure 6 includes a selection of the orbitals and excitations. In both complexes, the LUMO is essentially identical located on the ClPtPtCl axis and formed by the antisymmetrical σ* combination of the 5d_*z*^2^_ of the two platinum centers and the p_*z*_ orbitals of the chloride atoms (58% Pt–Pt and 26% Cl–Cl). Therefore, population of this orbital should cause strong distorted excited states with elongation of the Pt–Pt separation and of the Pt–Cl bonds. The symmetrical combination σ of the 5d_*z*^2^_ is mainly located on the HOMO–2, but this orbital has also a notable contribution of the phenylbenzothiazolate groups. For complex **5a**, the HOMO and H-1 are primarily located on the Me_2_N-pbt groups, while in the pbt complex, **4a** have also small platinum and Cl contributions (see Table S7). The calculated low lying S_1_–S_3_ (431 to 489 nm **4a** and 748–489 nm **5a**) transitions have very low oscillator strength and complex configuration (mixed LMMCT/LXCT/L’XCT **4a** and LMMCT/LXCT/ILCT **5a**) (L = R-pbt, X = Cl, L’ = pz). The most intense low energy-calculated band is S_4_ ascribed to H-4, H-2 to LUMO in **4a** and to a more complex configuration in **5a** (H-2, H-5, H-6, and H-10 to LUMO) having a mainly L’MMCT/L’XCT nature in **4a** and LMMCT/L’MCT/XC/MC in **5a**. The solid-state spectra also reflect the oxidation of the Pt centers exhibiting blue-shifted low-energy features in relation to the Pt^II^–Pt^II^ precursors (see Table S3, Figure S14).

#### Emission Properties and DFT Calculations

**Figure 7:**
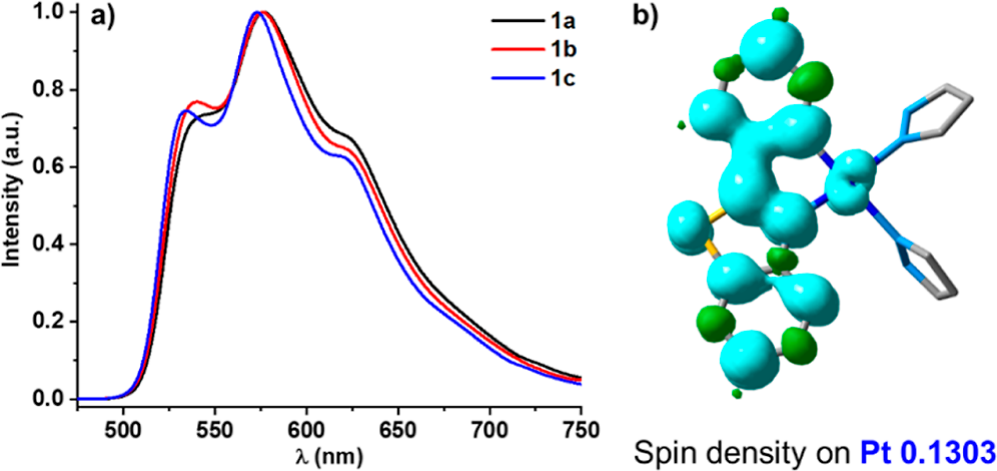
(a) Emission spectra (λ_ex_ 420 nm) of complexes **1** in THF solution (5 × 10^–4^ M) at 298 K and (b) spin density surface on T_1_ state of complex **1a**.

**Figure 8:**
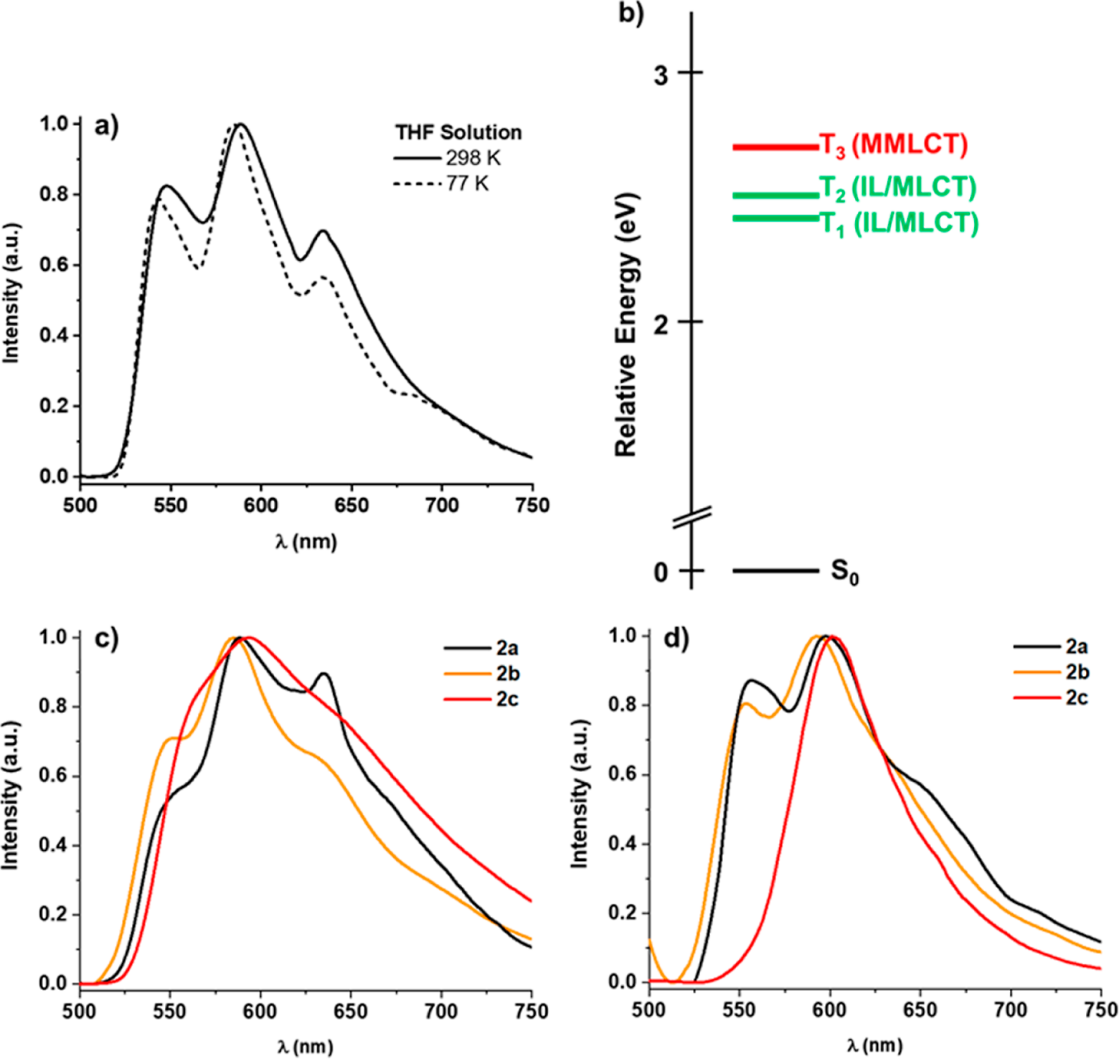
(a) Emission spectra (λ_ex_ 400–450 nm) of complex **2a** in THF solution at 298 and 77 K. (b) Relative energies and character of the vertical triplet excitations (T_1_–T_3_) for complex **2a**. Emission spectra of **2a**–**c** in solid state (c) at 298 (d) at 77 K.

**Figure 9:**
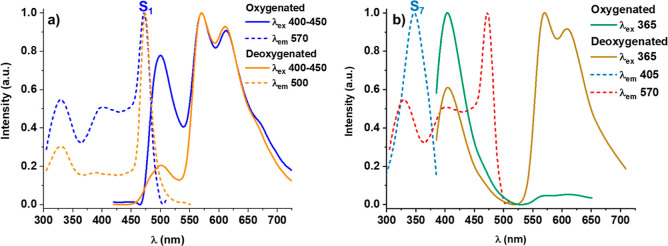
Excitation (····) and emission (—) spectra of complex **3a** in oxygenated and deoxygenated THF 5 × 10^–4^ M solution at 298 K upon excitation at (a) 400–450 and (b) 365 nm.

**Figure 10:**
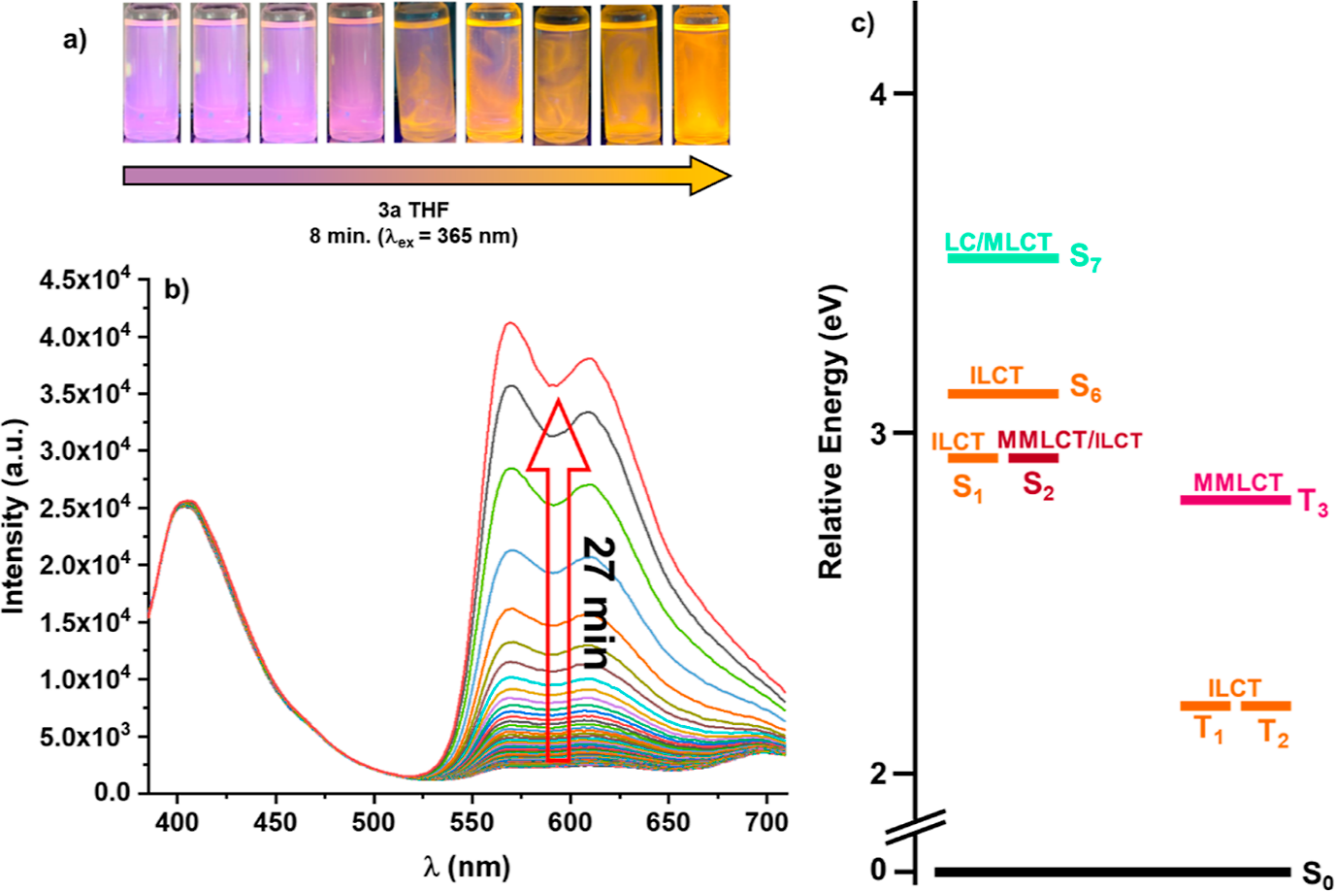
(a) Photographs showing the *switch-on* and enhancement of the phosphorescent emission of **3a** in an aerated THF solution upon excitation at 365 nm. (b) Emission spectra of **3a** in THF 5 × 10^–4^ M solution in the presence of O_2_ with different irradiation times. (c) Relative energy and character of the more intense vertical singlet and triplet excitations at the S_0_ geometry of **3a**.

The emission spectra of complexes **1** and **2a**–**5a** were registered in THF fluid solution (5 × 10^–4^ M for **1** and **2a**–**3a** and 5 × 10^–5^ M for **4a**), THF glasses at 77 K, polystyrene (PS) films at 10% wt, and in the solid state for all complexes at room and low temperatures (Tables 2 and S8, Figures 7–10 and S17–S19). Mononuclear complexes **1a**–**1c** show in the solid state, THF solution and doped films at 298 K a similar long-lived vibronically structured emission band (λ_max_ ∼ 540 nm), slight blue-shifted at low temperatures (λ_max_ ∼ 530 nm), attributed to a predominant intraligand transition (^3^IL) with some metal-to-ligand charge-transfer (^3^MLCT) characters. This emission is comparable to those reported for related neutral heteroleptic monomers [Pt(pbt)(L^X)] (L^X = O^O, N^O, P^O),^4d,17e,29^ and the assignment is further supported by optimization of the triplet excited (T_1_) state for complex **1a**^**+**^. As illustrated in Figure 7, the spin density surface at the optimized T_1_ state is centered on the pbt ligand with some contribution of the platinum (Pt ∼ 0.13). The adiabatic calculated emission wavelength (638 nm) exhibits the expected overestimated value in relation to the experimental data (∼540 nm, THF, 298 K). The calculated quantum yields are moderate in solution (10–21%, deoxygenated conditions, Table 2) but relatively low in rigid media (solid and PS, 1–8% Table S8), indicating aggregation-caused quenching characteristics (ACQs).^30^ The relatively strong π···π and S··π interactions between the pbt ligands in these rigid media, as found in their X-ray structures, could provide easy deactivation pathways and may account for the reduced efficiencies. Notwithstanding, it is worth noting that reports on luminescent mononuclear bis-pyrazole Pt^II^ complexes are rare.^31^ A comparison of the radiative and nonradiative rate constants (*k*_r_ and *k*_nr_) reveals that complex **1a** presents the highest *k*_nr_ value in relation to **1b** and **1c** (*k*_nr_ 2 × 10^5^**1a** vs 1.4 × 10^5^**1b**, and 1.5 × 10^5^**1c**) and the lowest *k*_r_ (2.2 × 10^4^**1a** vs 3.5 × 10^4^**1b** and 4 × 10^4^**1c**), resulting in a less efficient phosphor (φ 10% **1a** vs 20% **1b** and 21% **1c**). This could be associated with a less steric hindrance of the pyrazole ligand in **1a**, which provides less rigidity.

**Table 2:** Photophysical Data for Complexes **1a**–**1c** and **2a** and **3a** in THF Solution (5 × 10^–4^ M)^a^

	298 K						77 K
compound	λ_em_/nm	φ^b^	φ^c^	τ^d^/μs	*k*_r_^e^/s^–^^1^	*k*_nr_^f^/s^–^^1^	λ_em_/nm
**1a**^g^	543, 577_max_, 620	0.10	0.01	4.5	2.2 × 10^4^	2.0 × 10^5^	529, 569_max_, 618
**1b**^g^	540, 576_max_, 620	0.20	0.02	5.7	3.5 × 10^4^	1.4 × 10^5^	526, 567_max_, 616
**1c**^g^	534, 573_max_, 617	0.21	0.03	5.2	4.0 × 10^4^	1.5 × 10^5^	527, 568_max_, 615
**2a**^g^	550, 589_max_, 634	0.01	0.01	0.9	1.1 × 10^4^	1.1 × 10^6^	544, 585_max_, 634
**3a**^g^	405_max_, 570, 614^b^,^g^	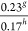	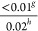	0.0016 [405]	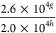	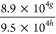	565_max_, 609
	500, 570_max_, 614^b^,^h^			0.0015 [500]			
	500, 570_max_, 614^c^,^h^			8.7 [570]			

a λ_ex_ 365–450 nm.

b Deoxygenated.

c Oxygenated.

d λ_ex_ 390 nm(LED).

e *k*_r_ = ϕ/τ_average_.

f *k*_nr_ = (1 – ϕ)/τ_average_ in deoxygenated conditions.

g λ_ex_ 365 nm.

h λ_ex_ 400–450 nm.

The bimetallic complex **2a** displays in all media a well-resolved vibronic emission profile with λ_max_ (∼540–550 nm) (Figure 8), close to that observed for complexes **1**, being therefore ascribed to a local mixed ^3^IL/^3^MLCT excited states. This behavior could be related to the relatively long Pt···Pt of 3.344 Å distance found for this complex, which makes it difficult to reach the σ^2^-σ^*1^ configuration characteristic of the ^3^MMLCT excited state.^10a,11b,26k^ The optimized S_0_ structure for complex **2a** shows a Pt–Pt separation of 3.2069 Å, comparable to that found in the crystal structure, and similar separation is found in the optimized T_1_ excited state (see Table S5), indicating the absence of short intramolecular platinum–platinum bonding interaction typical of the ^3^MMLCT excited state in T_1_. To confirm the nature of the emission, the lowest T_1_–T_3_ vertical excitations at the S_0_ geometry and the corresponding optimized T_1_–T_3_ were calculated (Tables S4, S9, S10 and Figure 8b). The two lowest vertical triplet excitations T_1,2_, which are close in energy (2.4 and 2.5 eV), and their corresponding optimized T_1,2_ states (676 nm) are located on one of the Pt(pbt) fragments having mixed ^3^IL/^3^MLCT nature. As illustrated in Figure 8b, the following excitation, T_3,_ has a ^3^MMLCT character but is located at 0.3 eV above T_1_. In **2a**, the metal contribution in T_**1**_ increases in relation to the mononuclear complex **1a** (0.2125 **2a** vs 0.1303 **1a**, Table S9) pointing to a higher metal-to-ligand charge-transfer (^3^MLCT) contribution in **2a**, in correlation with the lower lifetime recorded in THF solution for this bimetallic complex in comparison to the **1a** one (4.5, **1a** vs 0.9 μs, **2a**). The recorded quantum yield for **2a** in deoxygenated THF solution (1%) is lower than for **1a** (10%), but relatively similar in solid and PS (Table S8). This decrease in the quantum efficiency is attributable to an appreciable increase in *k*_nr_ (1.1 × 10^6^**2a** vs 2.0 × 10^5^**1a**), while there is a decrease in *k*_r_ (1.1 × 10^4^**2a** vs 2.2 × 10^4^**1a**).

For complexes **2b** and **2c**, only emission properties in the solid state were recorded (Figure 8c,d). Dimethylpyrazolate-bridge derivative (**2b**) shows, at room temperature and at 77 K, a similar band to that obtained for complex **2a** (∼550 nm) indicating emission from an ^3^IL/^3^MLCT excited state. For complex **2c**, with a bulkier substituent on the bridge group (R = ^i^Pr), the emission is broader at 298 K peaking at 560 nm and well stylized and red-shifted to 600 nm at 77 K (Figure 8d). This fact suggests the contribution of the low lying ^3^MMLCT excited state, which becomes predominant by decreasing the temperature.

Complex **3a**, comprising the donor–acceptor Me_2_N-pbt groups, displayed a significantly different behavior from that of the related complex **2a** (Table 2). It exhibits, upon excitation on the low energy band at 400–450 nm, and in a carefully deoxygenated THF solution at 298 K, in addition to a strong long-lived low energy band (LE) in the yellow-orange region (570 nm), a minor high energy feature (HE) in the blue-green region (500 nm) (see Figure 9a, orange line). The high energy feature is short-lived (1.5 ns) and displays characteristic mirror band-shaped with the longest wavelength absorption band, being therefore ascribed to S_1_ → S_0_ fluorescence having metal-perturbed intraligand charge-transfer ^1^ILCT (Me_2_Nph-to-bt) characters, whereas the LE-structured phosphorescent band is ascribed, according to calculations, to ^3^ILCT. In this complex **3a**, the two first triplet excitations T_1,2_ at the S_0_ geometry are essentially isoenergetic (556 nm) and exhibit an ILCT character (Figure 10c). The T_3_, with a MMLCT character, lies more separated from T_1_ (0.6 eV) than in **2a** (0.3 eV). The spin density distribution on the optimized T_1_ is mainly located on one of the cyclometalated groups with a lower metallic contribution in relation to the pbt-derivative **2a** (0.0809 **3a** vs 0.2125 **2a**), supporting primarily ^3^ILCT nature for the phosphorescent emission (Table S9). As was expected, the ratio F/P clearly increases in oxygenated solution due to a partial quenching of the low energy phosphorescent band (Figure 9a, blue line). The determined phosphorescence quantum yield in degassed solution is 17% and is reduced to 2% in air equilibrated solution. The excitation spectra of both bands correlate with the absorption spectrum in the low energy region, indicating that both emissions came from the same complex. However, the excitation spectra are not exactly identical in the region around 365–400 nm, where there is a notable MLCT contribution. This suggests that, while the fluorescent HE band mainly proceeds of excitation of the ^1^ILCT, the phosphorescent LE band is also notably populated from high-energy S_*n*_ excited states. Interestingly, we also observe that the emission depends on the excitation wavelength. Thus, upon excitation at 365 nm, a dual emission is also observed formed by a short-lived blue-shifted fluorescence band (1.6 ns) located at 405 nm and the structured low energy phosphorescent at 570 nm, which is extremely air sensitive being nearly absent in air-equilibrated THF solution (decreases from 23% in degassed solution to less of 1% in air equilibrated). The new fluorescence band is related to an excitation manifold located at 350 nm, whereas the excitation spectrum when detected the LE band is identical to that observed for the LE band upon exciting in the low energy region (400–450 nm) (Figure 9b). Our calculations suggest that the excitation S_7_, at 351.3 nm, has mixed ^1^MLCT/^1^LC nature with a remarkable platinum contribution and minor contribution of the NMe_2_ (Figure 10b). In agreement with this, the high quantum efficiency in **3a** upon excitation to 365 nm (ϕ = 23%) is mainly attributable to its higher *k*_r_ (2.6 × 10^4^) and lesser *k*_*n*r_ (8.9 × 10^4^) in relation to those obtained upon excitation to 400–450 nm (Table 2), probably related to the higher metal contribution in the LE emission using the high-energy excitation wavelengths. This wavelength dependence suggests the occurrence of hyper-intersystem crossing (HISC) from S_7_ (^1^MLCT/^1^LC) to T_1_, i.e., relaxation from S_7_ to T_1_ clearly competes with internal conversion (IC) to S_1_. It seems that the energy transfer from relaxed (^1^MLCT/^1^LC)* to ^1^ILCT* is nonefficient. This relatively rare behavior has been previously observed in some platinum complexes, being related to the presence of relaxed S_1_ states having strong ππ* characters with an essentially null metal contribution.^17d,32^ At 77 K, the fluorescence is lost and only the phosphorescence emission band at 565 nm is developed regardless of the wavelength used in the excitation.

Interestingly, we observed that in aerated THF solution, upon prolonged photoexcitation at 365 nm, a continuous enhancement of the phosphorescent band is observed, rising its maximum intensity in *ca* 27 min (Figure 10b). The process is also visible with a hand UV–vis lamp in which the initial violet emission, attributed to ^1^MLCT/^1^LC fluorescence, was gradually changing to a final orange enhanced emission (Figure 10a). The intense orange emission was switched off by simply shaking the solution allowing its oxygenation. A similar process, which is reversible and can be repeated several times, was also observed in DMSO but does not take place in other solvents, such as acetonitrile, toluene, or MeOH. This relatively rare behavior has been previously observed for us^17d^ and other groups,^33^ being explained by the occurrence of a local sensitization caused by energy transfer from the low energy triplet to ^3^O_2_ producing singlet ^1^O_2_ able to selectively react with the solvent (THF^34^ or DMSO^17d^), thus creating a free oxygen microenvironment that *switch-on* the phosphorescence (^3^ILCT). The strong sensibility of the phosphorescent emission in this complex (see Table 2) encourages us to determine its efficiency as an ^1^O_2_ sensitizer. The singlet oxygen generation of complex **3a** was examined in acetonitrile solution (5 × 10^–4^ M) on the infrared region detecting the characteristic emission profile of ^1^O_2_ at λ_em_ ∼ 1274 nm (Figure S21) upon excitation at both 365 and 400 nm, respectively. Phenalenone (PN), a universal reference compound which can be used in various solvents,^35^ has been employed. The measured quantum yield (ϕ_Δ_) of ^1^O_2_ was notably higher upon exciting at 365 nm than upon exciting at 400 nm (37% vs 11%), in agreement with the higher ^3^ILCT phosphorescence efficiency (23% vs 17%, Table 2). These values indicate that this complex can be used as a photosensitizer.

In the solid state, this complex (**3a**) displays a broader emission red-shifted at low temperatures (Figure S19), indicating some additional ^3^MMLCT contribution.

Because the initial structural report of a diplatinum d^7^–d^7^ complex, K_2_[Pt_2_(SO_4_)_4_(H_2_O)_2_],^36^ different types of high-valent diplatinum d^7^–d^7^, either with bridging and unbridged ligands, have been reported.^37^ These complexes have attracted a great interest in many efficient catalyst processes, such as the oxidation of unsaturated organic molecules, facilitated by the strong electron-withdrawing ability of the unusually high oxidation state of the Pt^III^ atom.^37a^ Initial suggestions^38^ and recent contributions^13,39^ support that these complexes can be sometimes key intermediate in the oxidation pathway from Pt^II^ to Pt^IV^. Indeed, their rich reactivity indicates that the electron distribution along the Pt–Pt bond can be viewed as a resonance structure between Pt^III^–Pt^III^ and Pt^II^–Pt^IV^.^37e,40^ Despite their rich chemistry, and also the well-known emissive properties of either Pt^II^ and Pt^IV^ complexes, reports on emissive d^7^–d^7^ derivatives are quite rare, mainly due to the fact that in these complexes the target orbital and therefore, the lowest-lying excited state possess metal–metal antibonding (dσ*_M-M_) character,^28^ being therefore short life and nonemissive. To the best of our knowledge, only three types of luminescent binuclear Pt^III^ complexes have been reported: (i) of the type [Pt_2_ (μ-pop)_4_X_2_]^4–^ (pop = P,P-pyrophosphite, P_2_O_5_H_2_^2–^; X = Cl, Br, SCN) or [Pt_2_ (μ-pop)_4_X_2_]^2–^ (X = py);^41^ (ii) type [Pt_2_ (μ-C_6_H_3_-5R-2-AsPh_2_)_4_X_2_] (R = methyl o isopropyl, X = Cl, Br, I)^14a^ and (iii) [Pt(C^N)(μ-pxdt)]_2_ (pxdt = oxadiazole-thiol). In these latter, donor–acceptor bridging ligands have been employed giving rise to emission from an excited state having ligand-to-metal–metal charge transfer-(LMMCT) character.^14b^

**Figure 11:**
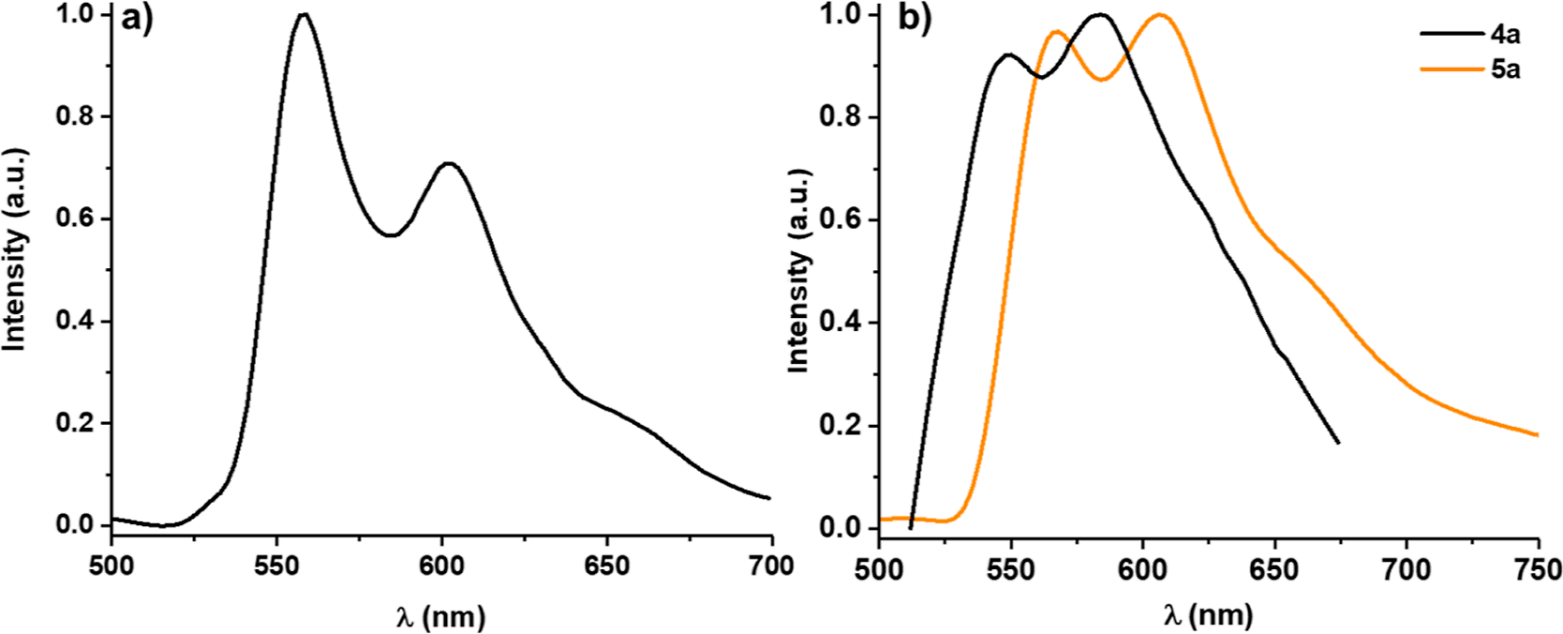
Emission spectra (λ_ex_ 365–420 nm) of (a) **4a** in THF solution (5 × 10^–5^ M) at 77 K and (b) of **4a** and **5a** in the PS film (10% wt).

The Pt^III^–Pt^III^ complexes (**4a** and **5a**) are weakly emissive, showing in PS rigid media and in glassy THF solution (only **4a**), a structured low-efficient (2% **4a** and <1% **5a** in PS) emission profile associated with a ^3^IL character centered on the R-pbt group (550 **4a** and 566 nm **5a**) (Figure 11). The lowest TD-DFT (T_1,2_ for **4a** and T_1–3_ for **5a**) vertical excitations at the S_0_ geometry (Table S4) are of LMMCT/LXCT in nature and are expected to be not emissive, in accordance with the lack of emission in fluid solution. The weak emission observed in rigid media is tentatively associated with close higher excited states (T_3_ for **4a** and T_4_ for **5a**) having a mainly ^3^ILCT character with a minor ^3^MLCT/^3^XLCT additional contribution for **4** (see Table S4). The calculated values (487.5 nm **4a** and 603 nm **5a**) agree with the experimental red shift observed for **5a** relative to **4a** attributed to the incorporation of the donor NMe_2_ group, which increases the energy of the HOMO decreasing the gap of the transition.

### Electrochemical Properties

The electrochemical properties of complexes **2a**–**5a** were investigated using cyclic voltammetry in anhydrous CH_2_Cl_2_ with (NBu_4_)PF_6_ as a supporting electrolyte in the dark.

The potentials and HOMO/LUMO energy estimations are listed in Table 3 and voltammograms covering the anodic (**2a**, **3a**) and cathodic (**2a**–**5a**) regions are depicted in Figure S22. Cyclometalated diplatinum complexes with butterfly or half-lantern shape exhibit mainly a two-electron oxidation process, assignable to the oxidation of the divalent species Pt_2_(II) to trivalent species Pt_2_(III);^8b,8d,12,42^ although in some cases, two one-electron redox waves corresponding to the Pt_2_(III)/Pt_2_(II) couple were observed.^43^ The Pt^II^–Pt^II^ complexes **2a** and **3a** show in the anionic window two bad resolved quasi-reversible or irreversible process with *E*^ox^ 0.63, 0.96 V **2a**, 0.73, and 1.07 V **3a** (vs Ag/AgCl), probably due to two steps of one-electron oxidation from Pt_2_(II,II) to Pt_2_(III,II) and to Pt_2_(III,III). The irreversibility of the oxidation processes is caused by the nucleophilic reactions of the coordinating solvent to the electrogenerated Pt^III^ species.^12^ The Pt^III^–Pt^III^ complexes **4a** and **5a** do not exhibit significant redox peaks by scanning the potential in the anodic direction.

**Table 3:** Electrochemical Data^a^ and HOMO/LUMO Energy Estimations for Complexes **2a–5a**

	cyclic voltammetry				DFT calculations	
	*E*^ox^^b^ (V)	*E*^red^^c^ (V)	*E*_HOMO_^d^ (eV)	*E*_LUMO_^d^ (eV)	*E*_HOMO_^e^ (eV)	*E*_LUMO_^e^ (eV)
**2a**	0.63	–1.01	–4.98	–3.35	–5.46	–2.00
	0.96					
**3a**	0.73	–1.10	–5.07	–3.24	–5.06	–1.67
	1.07					
**4a**		–0.69		–3.13	–6.28	–2.97
		–1.21				
**5a**		–1.07		–3.27	–5.12	–2.84

a All measurements were carried out in dark conditions at 298 K in 0.1 M solution of (NBu_4_)PF_6_ in dry CH_2_Cl_2_ at 100 mV s^–1^ vs Ag/AgCl reference electrode.

b Quasi-reversible or irreversible anodic peaks.

c Irreversible cathodic peaks.

d Estimated HOMO/LUMO energy by electrochemistry data [*E*_HOMO/LUMO_ = −(*E*^ox/red^ +4.8 – *E*^Fc/Fc+^)].

e Estimated HOMO/LUMO energy by DFT calculations.

Complexes constructed with the NMe_2_-pbt cyclometalating ligand show an irreversible reduction wave with shape and potential values that are similar (*E*^red^ −1.10 V Pt_2_^II^**3a**, −1.07 V Pt_2_^III^**5a**). However, the pbt complexes displays different reduction behavior. Thus, whereas the Pt_2_^II^ complex **2a** shows an irreversible wave at −1.01 V, two reduction waves at −0.69 and −1.21 V were resolved for **4a** (Pt_2_^III^). HOMO and LUMO energy levels were estimated from these CV data by using the relationship *E*_HOMO/LUMO_ = −(*E*^ox/red^ + 4.8 – *E*^Fc/Fc+^), where *E*^Fc/Fc+^ (0.45 V) is the potential of ferrocene vs Ag/AgCl and 4.8 eV is the energy level of ferrocene to the vacuum energy level. The calculated LUMO energies are between −3.13 and −3.35 V, being similar for the NMe_2_-pbt complexes (−3.24 **3a**, −3.27 V **5a**), what is an accordance with a LUMO mainly located in the cyclometalated ligand. Notwithstanding, the estimated HOMO and LUMO energies do not correlate well with those obtained by DFT calculations.

### Photocatalytic Studies

**Scheme 4:**
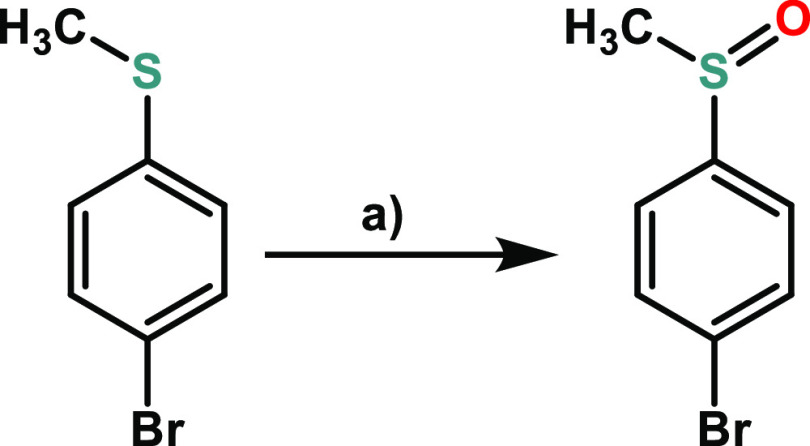
Photooxidation of *p-*Bromothioanisole to the Corresponding Sulfoxide Reagents and conditions: (a) CD_3_OD, Blue LED (460 nm), photosensitizer (**3a**).

In recent years, visible light photocatalysis has received a great attention allowing to furnish new molecules and structural motifs with lower energy consuming when compared with reactions under thermal or ultraviolet (UV).^44^ In this area, luminescent cyclometalated transition metals (Ru^II^, Ir^III^ and Pt^II^) are among the most frequently employed sensitizers for energy transfer and photoredox catalysis.^45^ In particular, some of these complexes have been previously described as efficient photosensitizers for the generation of reactive oxygen species (^1^O_2_, O_2_^•–^), being successfully employed as photocatalysts for the photooxidation of different organic molecules.^46^ Among the various oxidation reactions, the photo generation of sulfoxides from sulfides using oxygen as oxidant is of great interest,^21,23a,23c,47^ mainly due to its relevance in the synthesis of biologically active compounds used in the pharmaceutical industry and also in organic synthesis.^18^ We recently reported the ability of dimethylphenylbenzothiazole platinum complexes to generate, under photoexcitation, sensitized singlet oxygen (^1^O_2_) able to induce oxidation of DMSO to DMSO_2_.^17d^ In this line, the good ability of complex **3a** to photo sensitize singlet oxygen encouraged us to estimate its photocatalytic activity. In particular, we investigated the induced photooxidation ability of *p-*bromothioanisole (Scheme 4) under visible light (blue light; λ = 460 nm) in the presence of **3a** and oxygen as a model for heterogeneous catalysis.^48^ The evolution of the catalysis was monitored by NMR spectroscopy. Photosensitizer **3a** showed a good photostability under irradiation of blue light in suspension and solid state (measured by UV–vis spectroscopy from 0 to 50 h, Figure S23). This reaction was evaluated in different conditions and molar ratio of catalyst and substrate (Table 4, entries 1 and 2), with 1 and 5 mol % of the metal complex. In both cases, the reaction occurred with similar results allowing >95% conversion after 50 h of reaction. However, with 5% the reaction shows slight greater efficiencies at shorter times (3–4 h of reaction, Table S11). As illustration, after 15 h of reaction with 1% showed a 45% conversion, while with 5% a 55%. These results allow us to conclude that the increase of the amount of catalyst does not produce remarkable changes in the efficiencies.

**Table 4:** Heterogeneous Visible-Light Oxidative Reactions with Different Conditions

entry	% photosensitizer (%)	light	atmosphere	time (h)	conversion (%)
1	1	+	air	50	>95
2	5	+	air	50	>95
3^a^	5	+	air	36	>95
4	1	+	N_2_	50	–
5	1	–	air	50	–
6	–	+	air	50	–
7^b^	1	+	air	50	10
8^c^	1	+	air	50	23

a Double amount of reagent (*p-*bromothioanisole) and photosensitizer than in the entry 2.

b In the presence of DABCO.

c In the presence of BQ.

When the reaction was carried out with double amount of catalyst and substrate (Table 4, entry 3), the 95% of conversion was reached at 36 h, revealing that the increment in the concentration of the photocatalytic reactions improves the efficiency. Under hypoxic conditions, the conversion was considerably reduced because of the impossibility to produce ROS in the absence of oxygen (Table 4, entry 4). In the absence of light (Table 4, entry 5), no conversion was observed and, similarly, without a catalyst (**3a**) in the presence of air the reaction does not take place (Table 4, entry 6).

Generally, two mechanisms involving two main ROS intermediates such as ^1^O_2_ and O_2_^•–^ have been proposed for the photocatalytic oxidation of sulfides:^49^ (a) The photooxygenation promoted by ^1^O_2_ generated by a photosensitizer and (b) a photosensitized electron-transfer (ET) oxidation using ^3^O_2_ through O_2_^*–^ as an intermediate. In both mechanisms, a similar zwitterionic persulfide intermediate (R_1_R_2_S^+^O–O^–^) is proposed, which reacts with a second molecule of R_1_R_2_S leading to the formation of two molecular sulfoxides. Discrimination between both mechanisms is not an easy task.^21^ To get insights into the catalytic mechanism, several control experiments were carried out. Thus, the photocatalytic reaction was evaluated out in the presence of 1,4-diazabicyclo[2.2.2]octane (DABCO), a well-known singlet oxygen quencher, to evaluate the potential production of these species. As can be observed in the Table 2-entry 7, in the presence of DABCO (3 equiv), reagents (sulfide, **3a**), and light, the reaction is notably reduced (10%) and only traces of oxidized product were obtained, pointing to a key role of complex **3a** as an oxygen sensitizer. On the other hand, the addition of a superoxide radical (O_2_^•–^) quencher like 1,4-benzoquinone (BQ, 3 equiv) to the photooxidation process (Table 4, entry 8) also results in a remarkable decrease of the reaction yield to 23%, revealing a key role of these reactive oxygen species too. These observations suggest that both ^1^O_2_ and superoxide radicals play an important role in this photocatalytic reaction.

## Conclusions

In summary, we have prepared novel series of mononuclear (**1a–c)**, bimetallic (Pt^II^–Pt^II^) (**2a**–**c**, **3a**), and (Pt^III^–Pt^III^) (**4a**, **5a**) complexes incorporating phenylbenzothiazole (pbt) and 2-(4-dimethylaminophenyl)benzothiazole (Me_2_N-pbt) as cyclometalating chromophore groups and pyrazole (**1**) or pyrazolate bridging ligands (**2**–**5**). Experimental data and computational studies reveal the negligible influence of the pyrazole or pyrazolate bridging ligand on the optical properties of complexes **1a**–**c** and **2a**,**b**, which exhibit typical low-lying IL/MLCT electronic transitions. Only complex **2c**, incorporating the bulky 3,5-^i^Pr_2_pz bridging groups exhibits in the solid state an emission with some contribution of the ^3^MMLCT excited state, which is clearly predominant at low temperatures (560 nm at 298 K; 600 nm 77 K). **3a** incorporating the donor–acceptor Me_2_N-pbt ligand displays unusual dual, Fluorescence (^1^ILCT or ^1^MLCT/^1^LC) and phosphorescence (^3^ILCT) emissions depending on the excitation wavelength. The efficiency of the population of the triplet manifold increases upon photoexcitation of excited states having a higher metal d contribution. The phosphorescence can be reversibly photoinduced in oxygenated THF and DMSO solutions upon continuous excitation (365 nm, ∼ 15 min) and quenched by shaking. The complex also photosensitizes ^1^O_2_, with a higher quantum yield at λ_ex_ of 365 nm than at 400 nm (37 vs 11%). Computed results for the low-lying T_1_–T_3_ excited states in complexes **2a** and **3a** indicate that T_1,2_ have a mixed ^3^IL/^3^MLCT nature in **2a** and mainly ^3^ILCT character in **3a**. In both complexes, the T_3_ has a ^3^MMLCT character and lies more separated from T_1_ (0.6 eV) in **3a** than in **2a** (0.3 eV). The diplatinum complexes **4a** and **5a** increase the small number of luminescent d^7^–d^7^ compounds reported, with a weak emission, in rigid media, ascribed to ^3^ILCT. Finally, complex **3a**, which demonstrates the ability to photosensitize singlet oxygen, has been further applied for the photooxidation of *p*-bromothioanisol under visible light (460 nm). Control of this reaction suggests that both, ^1^O_2_ and superoxide radicals, play an important role in this photocatalytic reaction.

## Supporting Information Available

Characterization of complexes (NMR spectra, crystal data), photophysical properties and computational details, and photocatalytic studies (PDF)
