# Supplementary material for: Phenylbenzothiazole-Based Platinum(II) and Diplatinum(II) and (III) Complexes with Pyrazolate Groups: Optical Properties and Photocatalysis
Source: Inorg Chem. 2024 Jan 10;63(3):1589–606. doi: 10.1021/acs.inorgchem.3c03532 (PMC10806813; doi:10.1021/acs.inorgchem.3c03532)
Supplement: Supplementary file 1 — ic3c03532_si_001.pdf [file ic3c03532_si_001.pdf]

Phenylbenzothiazole-Based Platinum(II) and Diplatinum(II) and (III) Complexes with Pyrazolate Groups: Optical Properties and Photocatalysis

David Gómez de Segura, Andrea Corral-Zorzano, Eduardo Alcolea, M. Teresa Moreno* and Elena Lalinde*

Departamento de Química, Instituto de Investigación en Química (IQUR), Complejo Científico Tecnológico, Universidad de La Rioja, Madre de Dios 53, 26006 Logroño, Spain. E-mail: teresa.moreno@unirioja.es; elena.lalinde@unirioja.es

Contents:

Contents:	Page
**1.- Experimental Section**	S2
**2.- NMR Spectra**	S8
**3.- Mass Spectra**	S15
**4.- Crystal Structures**	S18
**5.- Photophysical Properties and Theoretical Calculations**	S24
**6.- Electrochemical Properties**	S44
**7.- Photocatalytic Studies**	S45

1.- Experimental Section

General Comments. All reactions were carried out under an atmosphere of dry argon, using standard Schlenk techniques. Solvents were obtained from a solvent purification system (M-BRAUN MS SPS-800). Mass spectra were recorded with electrospray ionization on an interphase ESI/APCI Bruker Microtof-Q spectrometer with positive ion mode, with MeOH/H_2O 90/10 and 0.1% formic acid as a mobile phase or on a Microflex MALDI-TOF Bruker (MALDI) spectrometer operating in the linear and reflector modes using DCTB as matrix. IR spectra of powders were obtained on a Nicolet Nexus FT-IR covering the region between 4000 and 200 cm^{-1} and data processing was carried out with Omnic. NMR spectra were recorded on Bruker AVANCE ARX 300 or 400 spectrometers at 298 K. Chemical shifts are reported in parts per million (ppm) relative to external standards ($SiMe_4$ for ^1H and $^{13}C\{^1H\}$, $CFCl_3$ for $^{19}F\{^1H\}$ and H_3PO_4 for $^{31}P\{^1H\}$) and all coupling constants are given in hertz (Hz). The UV-VIS absorption spectra were measured with a Hewlett Packard 8453 spectrophotometer. Diffuse Reflectance UV-vis (DRUV) spectra were carried out in SiO_2 pellets, using a Shimazdu UV-3600 spectrophotometer with a Harrick Praying Mantis accessory, and recalculated following the Kubelka Munk function. Excitation and emission spectra were obtained with an Edimburg FLS 1000 spectrofluorimeter. Lifetime measurements on the solid state and PS films (> 10 μs) were performed with an Edimburg FLS 1000 spectrofluorimeter with μF2 pulse lamp (Power: 100 W, Fuse: 3.15 Amp A/S). The lifetimes in THF solution (below 10 μs) at 298 K were measured with a Datastation HUB-B with a nanoLED controller, using the technique "Time Correlated Single Photon Counting" (TCSPC). The nanoLEDs employed for lifetime measurements were of 390 nm with pulse lengths of 1.2 ns. The decay data were treated with the software DAS6 (Jobin Yvon-Horiba). The absolute Quantum Yields were determined with a Hamamatsu Absolute PL Quantum Yield Measurement System. Cyclic voltammograms were registered on a potenciostat Voltalab PST 050 with the CV cell consisting of a platinum disk as working electrode, a Pt wire counter electrode and an Ag/AgCl reference electrode. The measurements were carried out at 298 K under N_2 atmosphere, using degassed 5×10^{-4} M solutions of the complexes in dry CH_2Cl_2 and 0.1 M $(NBu_4)PF_6$ as the supporting electrolyte. The ferrocene/ferricinium couple served as the internal reference (+0.45 V *vs* Ag/AgCl). The starting materials [Pt(pbt)Cl(DMSO)][1] and [Pt(Me_2N-pbt)Cl(DMSO)][2] were prepared

according to the published procedure. Other commercially available reagents were used as received.

Synthesis of [Pt(pbt)(pzH)$_2$]PF$_6$ (1a)

Pyrazole (0.026 g, 0.385 mmol) and $TlPF_6$ (0.0672 g, 0.192 mmol) were added to a suspension of [Pt(pbt)Cl(DMSO)] (0.101 g, 0.194 mmol) in 25 mL of acetone. After 6 h of stirring at room temperature, the white solid (TlCl) was filtered through celite and washed with acetone (3 x 5 mL). The filtrate was evaporated to dryness and the residue treated with Et_2O (3 x 5 mL) to obtain **1a** as a pale-yellow solid (0.114 g, 86 %). Anal. Calcd for $C_{19}H_{16}F_6N_5PPtS$ (686.47): C, 33.24; H, 2.35; N, 10.20; S, 4.67. Found: C, 33.50; H, 2.55; N, 9.96; S, 4.82 %. ESI (+): *m/z* (%): 877.0280 $[Pt_2(pbt)_2(pz)]^+$ (23), 945.0667 $[Pt_2(pbt)_2(pz)_2+H]^+$ (100), 967.0450 $[Pt_2(pbt)_2(pz)_2+Na]^+$ (12). IR (cm^{-1}): υ(N-H) 3363 (m, br), υ(PF_6) 841 (vs), υ(PF_6) 557 (s), υ(Pt-N) 471 (w). Λ_M (Acetone 5 x 10^{-4} M): 128.6 Ω^{-1} cm^2 mol^{-1}. ^{1}H NMR (400 MHz, CD_3COCD_3): δ = 13.66 (s, NH'), 13.60 (s, NH''), 8.32 (s, a, $H^{5''}$), 8.25 (s, a, $H^{5'}$), 8.21 (d, $^3J_{H-H}$ = 8.2, H^4), 8.17 (s, a, $H^{3''}$), 8.10 (s, $^3J_{Pt-H}$ = 20.3, $H^{3'}$), 7.79 (d, $^3J_{H-H}$ = 7.4, H^8), 7.52 (t, $^3J_{H-H}$ = 7.8, H^5), 7.33 (t, $^3J_{H-H}$ = 7.8, H^6), 7.24 (t, $^3J_{H-H}$ = 7.4, H^9), 7.11 (t, $^3J_{H-H}$ = 7.3, H^{10}), 6.81 (bs, $H^{4''}$), 6.72 (bs, $H^{4'}$), 6.26 (d, $^3J_{H-H}$ = 7.9, $^3J_{Pt-H}$ = 43.3, H^{11}), 6.18 (d, $^3J_{H-H}$ = 8.8, H^7). ^{13}C{^{1}H} NMR (100.6 MHz, CD_3COCD_3): δ = 185.2 (s, C^2), 150.0 (s, C^{7a}), 142.6 (s, $C^{3'}$) 142.5 (s, $C^{3''}$), 142.2 (s, C^{13}), 140.2 (s, C^{12}), 133.9 (s, $C^{5'}$), 133.8 (s, $C^{5''}$), 133.6 (s, C^{11}), 132.7 (s, C^{10}), 131.7 (s, C^{3a}), 129.1 (s, C^6), 127.1 (s, C^5), 126.5 (s, C^8), 126.2 (s, C^9), 124.8 (s, C^4), 119.4 (s, C^7), 108.9 (m, $C^{4'}$, $C^{4''}$). ^{31}P{^{1}H} NMR (162.1 MHz, CD_3COCD_3): δ = -144.3 (sept, $^1J_{P-F}$ = 706, PF_6). ^{19}F{^{1}H} NMR (376.5 MHz, CD_3COCD_3): δ = -72.6 (d, $^1J_{P-F}$ = 706, PF_6).

Synthesis of [Pt(pbt)(3,5-Me$_2$pzH)$_2$]PF$_6$ (1b)

Complex **1b** was obtained as a pale-yellow solid (0.121 g, 85 %) following the same procedure as **1a** starting from 3,5-Me$_2$pzH (0.037 g, 0.385 mmol), $TlPF_6$ (0.067 g, 0.192 mmol) and [Pt(pbt)Cl(DMSO)] (0.1006 g, 0.194 mmol). Anal. Calcd for $C_{23}H_{24}F_6N_5PPtS$ (742.58): C, 37.20; H, 3.26; N, 9.43; S, 4.32. Found: C, 37.58; H, 3.58; N, 9.20; S, 4.54 %. ESI (+): *m/z* (%): 597.1370 $[M-PF_6]^+$ (100), 501.0688 $[M-PF_6\text{-}3,5\text{-}Me_2pzH]^+$ (49). IR (cm^{-1}): υ(N-H) 3384 (m, br), υ(PF_6) 845 (vs), υ(PF_6) 557 (s), υ(Pt-N) 459 (w). Λ_M (Acetone 5 x 10^{-4} M): 135.4 Ω^{-1} cm^2 mol^{-1}. ^{1}H NMR (400 MHz, CD_3COCD_3): δ = 12.84 (s, NH'), 12.77 (s, NH''), 12.56 (s, NH'$_t$), 12.51 (s, NH''$_t$), 8.21 (d, $^3J_{H-H}$ = 7.3, H^4), 7.76

(d, $^{3}J_{H\text{-}H}$ = 7.3, H^8), 7.52 (t, $^{3}J_{H\text{-}H}$ = 7.3, H^5), 7.37 (t, $^{3}J_{H\text{-}H}$ = 8.0, H^6), 7.23 (t, $^{3}J_{H\text{-}H}$ = 7.8, H^9), 7.14-7.07 (m, H^{10}), 6.40-6.28 (m, 4H, $H^{4'}$ $H^{4''}$ $H^{4'}{}_t$ $H^{4''}{}_t$), 6.26-6.19 (m, 2H, H^{11} H^7), 2.47-2.35 (m, 12H, H^{Me}). $^{13}C\{^1H\}$ NMR (100.6 MHz, CD_3COCD_3): δ = 185.3 (s, C^2), 152,8 – 151.3 (m, 4C, $C^{3'}$ $C^{3''}$ $C^{3'}{}_t$ $C^{3''}{}_t$), 150.2 (s, C^{7a}),145.1(m, 4C, $C^{4'}$,$C^{4''}$,$C^{4'}{}_t$,$C^{4''}{}_t$), 142.1 (s, C^{13}), 140.7(s, C^{12}), 133.8 (s, $^{4}J_{Pt\text{-}H}$ = 56.9, C^{11}), 132.8 (s, C^{10}), 131.8 (s, C^{3a}), 129.2 (s, C^6), 127.2 (s, C^5), 126.4 (s, C^8), 126.1 (s, C^9), 124.7 (s, C^4), 119.3 (s, C^7), 108.6-107.2 (m, 4C, $C^{5'}$ $C^{5''}$ $C^{5'}{}_t$ $C^{5''}{}_t$), 14.7-13.9 (m, 4C, C^{Me}). $^{31}P\{^1H\}$ NMR (162.1 MHz, CD_3COCD_3): δ = -144.3 (sept, $^{1}J_{P\text{-}F}$ = 706, PF_6). $^{19}F\{^1H\}$ NMR (376.5 MHz, CD_3COCD_3): δ = -72.6 (d, $^{1}J_{P\text{-}F}$ = 706, PF_6).

Synthesis of [Pt(pbt)(3,5-$^{i}Pr_2$pzH)$_2$]PF_6 (1c)

Complex **1c** was obtained as a pale-yellow solid (0.180 g, 90 %) following the same procedure as **1a** starting from 3,5-$^{i}Pr_2$pzH (0.072 g, 0.472 mmol), $TlPF_6$ (0.082 g, 0.233 mmol) and [Pt(pbt)Cl(DMSO)] (0.121 g, 0.234 mmol). Anal. Calcd for $C_{31}H_{40}F_6N_5PPtS$ (854.79): C, 43.56; H, 4.72; N, 8.19; S, 3.75. Found: C, 43.35; H, 4.98; N, 8.05; S, 4.02 %. ESI (+): *m/z* (%): 709.2633 [M-PF_6]$^+$ (100), 557.1310 [M-PF_6-3,5-$^{i}Pr_2$pzH]$^+$ (21). IR (cm^{-1}): υ(N-H) 3369 (m, br), υ(PF_6) 845 (vs), υ(PF_6) 557 (s), υ(Pt-N) 467 (w). Λ_M (Acetone 5 x 10^{-4} M): 126.4 Ω^{-1} cm^2 mol^{-1}. ^1H NMR (300 MHz, CD_3COCD_3): δ = 13.06 (s, NH'), 12.99 (s,NH''), 8.21 (dd, $^{3}J_{H\text{-}H}$ = 8.1, $^{4}J_{H\text{-}H}$ = 0.7, H^4), 7.78 (dd, $^{3}J_{H\text{-}H}$ = 7.5, $^{4}J_{H\text{-}H}$ = 1.0, H^8), 7.53 (td, $^{3}J_{H\text{-}H}$ = 7.3, $^{4}J_{H\text{-}H}$ = 1.2, H^5), 7.37 (td, $^{3}J_{H\text{-}H}$ = 8.0, $^{4}J_{H\text{-}H}$ = 1.2, H^6), 7.23 (td, $^{3}J_{H\text{-}H}$ = 7.3, $^{4}J_{H\text{-}H}$ = 1.2, H^9), 7.12 (td, $^{3}J_{H\text{-}H}$ = 7.7, $^{4}J_{H\text{-}H}$ = 1.2, H^{10}), 6.53 (d, $^{4}J_{H\text{-}H}$ = 2.3, $H^{4'}$), 6.41 (d, $^{4}J_{H\text{-}H}$ = 2.5, $H^{4''}$), 6.15 (d, $^{3}J_{H\text{-}H}$ = 7.5, H^{11}), 6.01 (d, $^{3}J_{H\text{-}H}$ = 8.9, H^7), 3.71 (sept, $^{3}J_{H\text{-}H}$ = 7.1 $H^{CH\,(pz)}$), 3.64 (sept, $^{3}J_{H\text{-}H}$ = 7.1 $H^{CH\,(pz)}$), 3.24 (sept, $^{3}J_{H\text{-}H}$ = 7.1 $H^{CH\,(pz)}$), 3.17 (sept, $^{3}J_{H\text{-}H}$ = 7.1 $H^{CH\,(pz)}$), 1.41-0.92 (m, 24H, $H^{Me\text{-}iPr}$). $^{13}C\{^1H\}$ NMR (75.5 MHz, CD_3COCD_3): δ = 185.1 (s, C^2), 162.7 (s, $C^{5''}$), 161.8 (s, $C^{5'}$), 156.0 (s, $C^{3'}$), 155.6 (s, $C^{3''}$), 154.8 (s, C^{3a}), 149.9 (s, C^{7a}), 141.9 (s, C^{13}), 141.0 (s, C^{12}), 133.8 (s, C^{11}), 132.7 (s, C^{10}), 128.9 (s, C^5), 127.0 (s, C^6), 126.3 (s, C^8), 125.9 (s, C^9), 124.7 (s, C^7), 119.2 (s, C^4), 101.4 (s, $C^{4'}$), 100.9 (s, $C^{4''}$), 27.5, 27.0, 26.9 (s, 4C, CH-pz),23.7-22.2 (m, 8C, $C^{Me\ iPr}$). $^{31}P\{^1H\}$ NMR (91.2 MHz, CD_3COCD_3): δ = -144.3 (sept, $^{1}J_{P\text{-}F}$ = 706, PF_6). $^{19}F\{^1H\}$ NMR (282.4 MHz, CD_3COCD_3): δ = -72.6 (d, $^{1}J_{P\text{-}F}$ = 706, PF_6).

Synthesis of [Pt(pbt)(μ-pz)]$_2$ (2a)

Procedure a: A solution of complex **1a** (0.206 g, 0.300 mmol) in acetone (20 mL) was treated with excess of NEt_3 (1.5 mL). The mixture was stirred for 24 h to obtain complex **2a** as an orange-yellow solid (0.074 g, 53 %). Anal. Calcd for $C_{32}H_{22}N_6Pt_2S_2$ (944.84): C, 40.68; H, 2.35; N, 8.89; S, 6.79. Found: C, 40.36; H, 2.48; N, 8.65; S, 6.49 %. ESI (+): *m/z* (%): 945.0658 $[M+H]^+$ (100), 967.0420 $[M+Na]^+$ (16), 1890.1146 $[2M+H]^+$ (15). IR (cm^{-1}): υ(Pt-N) 465 (m). ^1H NMR (400 MHz, THF-d^8): δ = 7.89 (d, $^3J_{H-H}$ = 7.6, H^7), 7.75 (s, $^3J_{Pt-H}$ = 14.1, $H^{3'}$), 7.89 (d, $^3J_{H-H}$ = 7.1, H^8), 7.50 (s, $H^{5'}$), 7.40 (d, $^3J_{H-H}$ = 7.1, H^{11}), 7.31 (t, $^3J_{H-H}$ = 7.4, H^6), 7.25 (t, $^3J_{H-H}$ = 8.0, H^5), 7.05-6.96 (m, H^{10}, H^9, H^4), 6.34 (s br, $H^{4'}$).

Procedure b: Pyrazole (0.027 g, 0.386 mmol) and excess of KOH (0.217 g, 3.86 mmol) were added to a solution of [Pt(pbt)Cl(DMSO)] (0.201 g, 0.386 mmol) in a mixture of EtOH/Acetone (15/10 mL). After 24 h of stirring the suspension was evaporated to dryness. The brown residue was extracted with CH_2Cl_2 (3 x 5 mL) and filtered through celite. The filtrate was evaporated to dryness to obtain an orange-yellow solid (0.116, 64 %) identified as a mixture of *anti* and *syn* isomers of **2a** (5:1 *anti/syn*).

Synthesis of [Pt(pbt)(μ-3,5-Me_2pz)]$_2$ (2b)

Complex **2b** was obtained as an orange solid (0.046 g, 56 %) following the same procedure as **2a** starting from [Pt(pbt)(3,5-Me_2pzH)$_2$]PF_6 (**1b**) (0.122 g, 0.1641 mmol) and NEt_3 (1.5 mL). Anal. Calcd for $C_{36}H_{30}N_6Pt_2S_2$ (1000.95): C, 43.20; H, 3.02; N, 8.40; S, 6.41. Found: C, 43.57; H, 3.32; N, 8.20; S, 6.61 %. MALDI-TOF (+): *m/z* (%): 1000.2020 $[M+H]^+$ (100). IR (cm^{-1}): υ(Pt-N) 440 (m).

Synthesis of [Pt(pbt)(μ-3,5-$^iPr_2$pz)]$_2$ (2c)

Complex **2c** was obtained as an orange solid (0.041 g, 53 %) following the same procedure as **2a** starting from [Pt(pbt)(3,5-$^iPr_2$pzH)$_2$]PF_6 (**1c**) (0.120 g, 0.140 mmol) and NEt_3 (1.5 mL). Anal. Calcd for $C_{44}H_{46}N_6Pt_2S_2$ (1113.16): C, 47.48; H, 4.17; N, 7.55; S, 5.76. Found: C, 47.16; H, 4.13; N, 7.71; S, 5.47 %. MALDI-TOF (+): *m/z* (%): 1112.051 $[M+H]^+$ (100). IR (cm^{-1}): υ(Pt-N) 445 (m).

Synthesis of [Pt(Me_2N-pbt)(μ-pz)]$_2$ (3a)

Procedure a: A suspension of [Pt(Me_2N-pbt)Cl(DMSO)] (0.109 g, 0.193 mmol), $TlPF_6$ (0.068 g, 0.193 mmol) and pyrazole (0.013 g, 0.194 mmol) was stirred in acetone (20 mL). After 6h, the precipitate was filtered through celite and washed with acetone (2 x 5 mL). The filtrate was reduced to 20 mL and added NEt_3 (2.5 mL). The mixture was stirred for 4h to give complex **3a** (0.074 g, 74 %) as a yellow solid, identified as the *anti*-isomer.
Procedure b: Pyrazole (0.012 g, 0.386 mmol) and excess of KOH (0.100 g, 1.77 mmol) were added to a solution of [Pt(Me_2N-pbt)Cl(DMSO)] (0.100 g, 0.177 mmol) in a mixture of EtOH/Acetone (15/10 mL). After 24 h of stirring the suspension was evaporated to dryness. The yellow residue was treated with water (10 mL) and the solid washed with ^{i}PrOH (5 mL) and Et_2O (10 mL) to obtain a yellow solid (0.062 g, 68 %), identified as the *anti*-isomer of complex **3a**.
Anal. Calcd for $C_{36}H_{32}N_8Pt_2S_2$ (1030.98): C, 41.94; H, 3.13; N, 10.17; S, 6.22. Found: C, 41.64; H, 3.33; N, 9.95; S, 6.02 %. MALDI-TOF (+): *m/z* (%): 1029.961 $[M+H]^+$ (100). IR (cm^{-1}): υ(Pt-N) 456 (m). ^{1}H NMR (400 MHz, THF-d^8): δ = 7.76 (d, $^3J_{H-H}$= 2.2, $H^{5'}$), 7.74 (d, $^3J_{H-H}$= 8.3, H^7), 7.46 (d, $^3J_{H-H}$= 2.1, $H^{3'}$),7.37 (d, $^3J_{H-H}$= 8.5, H^8), 7.15 (t, $^3J_{H-H}$= 7.5, H^6), 7.06 (t, $^3J_{H-H}$= 8.6, H^5), 6.82 (d, $^3J_{H-H}$= 8.3, H^4), 6.64 (d, $^4J_{H-H}$= 2.4, H^{11}), 6.40 (dd, $^3J_{H-H}$= 8.6, $^4J_{H-H}$= 2.2, H^9), 6.30 (t, $^3J_{H-H}$= 2.1, $H^{4'}$), 2.43 (s, 6H NMe_2).

Synthesis of [Pt(pbt)(μ-pz)Cl]$_2$ (4a)

Procedure a: A mixture of complex **2a** (0.134 g, 0.142 mmol) and $PhICl_2$ (0.058 g, 0.211 mmol) in 20 mL of CH_2Cl_2 was stirred at 0 ºC. After 8 h, the solid was filtered and washed with hexane (3 x 5 mL) to obtain **4a** as a yellow solid (0.085 g, 59 %).
Procedure b: A suspension of complex **2a** (0.074 g, 0.108 mmol) in $CHCl_3$ (15 mL) was stirred under sun-light. After 24 h, the yellow solid was filtered and washed with Et_2O (3 x 5 mL) to obtain **4a** (0.049 g, 62 %). Anal. Calcd for $C_{32}H_{22}Cl_2N_6Pt_2S_2$ (1015.75): C, 37.84; H, 2.18; N, 8.27; S, 6.31. Found: C, 37.65; H, 2.17; N, 8.42; S, 6.18 %. ESI (+): *m/z* (%): 979.0327 $[M-Cl]^+$ (100), 1037.9883 $[M+Na]^+$ (5), 2029.9928 $[2M+H]^+$ (9). IR (cm^{-1}): υ (Pt-N) 449 (w), υ (Pt-Cl) 345 (w). ^{1}H NMR (400 MHz, $CDCl_3$): δ = 8.15 (s, a, $H^{5'}$), 7.98 (s, a, $H^{3'}$), 7.66 (m, H^7), 7.53 (m, H^4), 7.37-7.30 (m, H^5, H^6), 7.18 (d, $^3J_{H-H}$ = 7.7, H^8), 6.56 (t, $^3J_{H-H}$ = 7.7, H^9), 6.40 (s, $H^{4'}$), 6.36 (d, $^3J_{H-H}$ = 7.8, $^3J_{Pt-H}$ = 31.1, H^{11}), 6.08 (t, $^3J_{H-H}$ = 7.5, H^{10}).

Synthesis of [Pt(Me_2N-pbt)(μ-pz)Cl]$_2$ (5a)

Complex **5a** was obtained as a yellow solid (0.096 g, 63 %) following the same procedure than complex **4a** (**Procedure a**), starting from **3a** (0.143 g, 0.139 mmol) and 0.057 g (0.209 mmol) of $PhICl_2$. Anal. Calcd for $C_{36}H_{32}Cl_2N_8Pt_2S_2$ (1101.88): C, 39.24; H, 2.93; N, 10.17; S, 5.82. Found: C, 39.49; H, 2.82; N, 10.01; S, 6.03 %. MALDI (+) m/z (%): 1065.02 (100) $[M\text{-}Cl]^+$, 1029.97 (38) $[M\text{-}2Cl+H]^+$. IR (cm^{-1}): υ (Pt-N) 453 (w), υ (Pt-Cl) 352 (w). ^1H NMR (400 MHz, $CDCl_3$) δ = 8.15 (d, $^3J_{Pt\text{-}H}$ = 8.5, $^3J_{H\text{-}H}$ = 2.3, $H^{5'}$), 7.97 (d, $^3J_{Pt\text{-}H}$ = 12.3, $^3J_{H\text{-}H}$ = 2.1, $H^{3'}$), 7.59 (d, $^3J_{H\text{-}H}$ = 7.5, H^7), 7.43 (d, $^3J_{H\text{-}H}$ = 7.5, H^4), 7.16-7.06 (m, 2H , H^5 H^6), 7.00 (d, $^3J_{H\text{-}H}$ = 8.4, H^8), 6.38 (t, $^3J_{H\text{-}H}$ = 2.0, $H^{4'}$), 5.81 (dd, $^3J_{H\text{-}H}$ = 9.2, $^4J_{H\text{-}H}$ = 2.4, H^9), 5.45 (d, $^3J_{Pt\text{-}H}$ = 35.2, $^4J_{H\text{-}H}$ = 2.3 ,H^{11}), 2.55 (s, 6H NMe_2). $^{13}C\{^1H\}$ NMR (100.6 MHz, $CDCl_3$) δ = 177.9 (s, C^2), 150.5 (s, C^{10}), 147.9 (s, C^{7a}), 138.9 (s, C^{12}), 136.8 (s, $C^{5'}$), 133.7 (s, $C^{3'}$), 129.2 (s, C^{3a}), 127.0 (s, C^8), 126.7 (s, C^5/C^6), 123,7 (s, C^{13}), 123.6 (s, C^5/C^6), 121.8 (s, C^7), 119.5 (s, C^4), 112.7 (s, C^{11}), 107.4 (s, C^9), 106.8 (s, $C^{4'}$), 39.5 (s, C-Me^{NMe2}).

2.- NMR Spectra

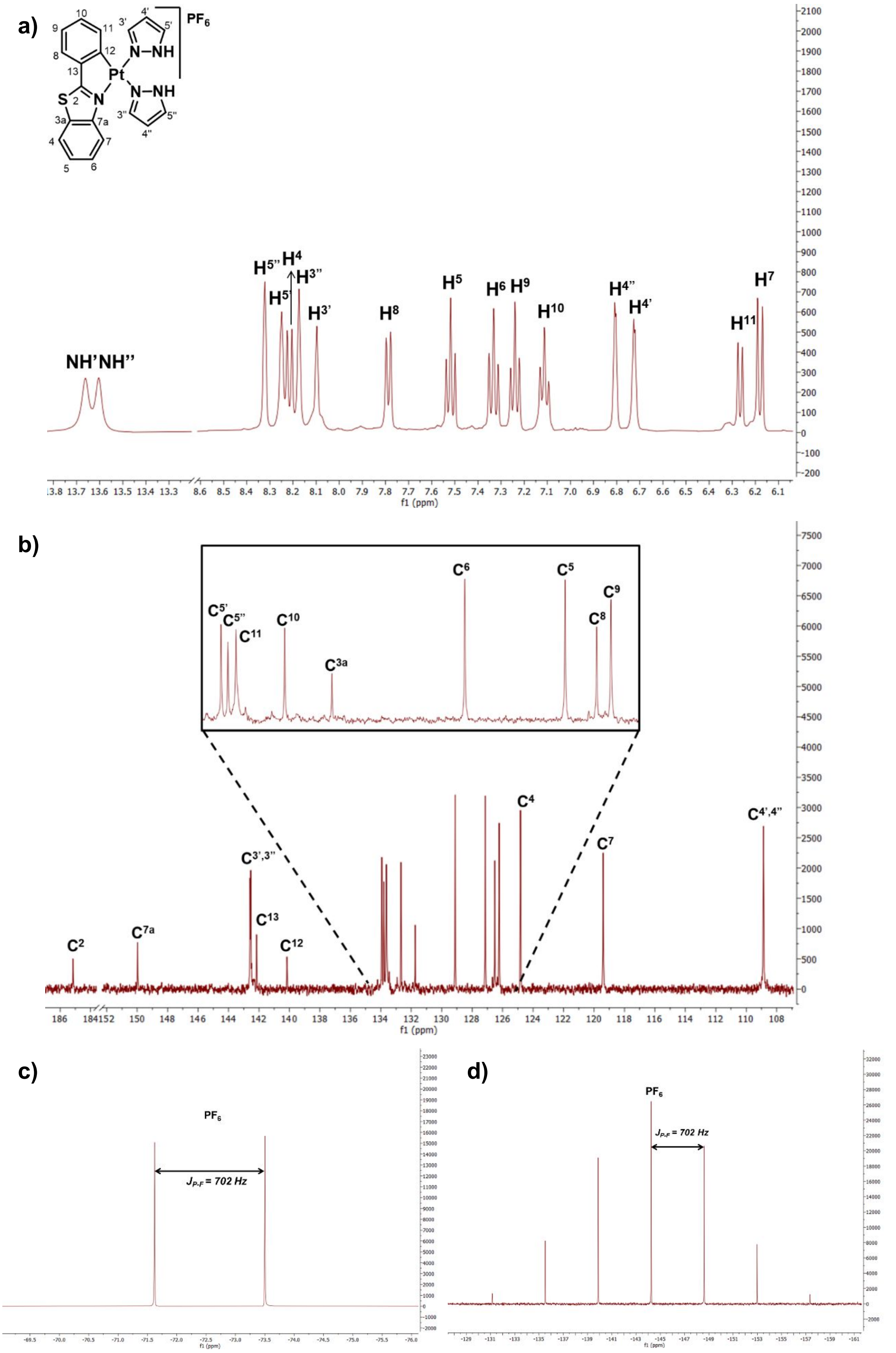

Figure S1. a) ^{1}H, b) $^{13}C\{^{1}H\}$, c) $^{19}F\{^{1}H\}$ and d) $^{31}P\{^{1}H\}$ NMR spectra of complex **1a** in CD_3COCD_3.

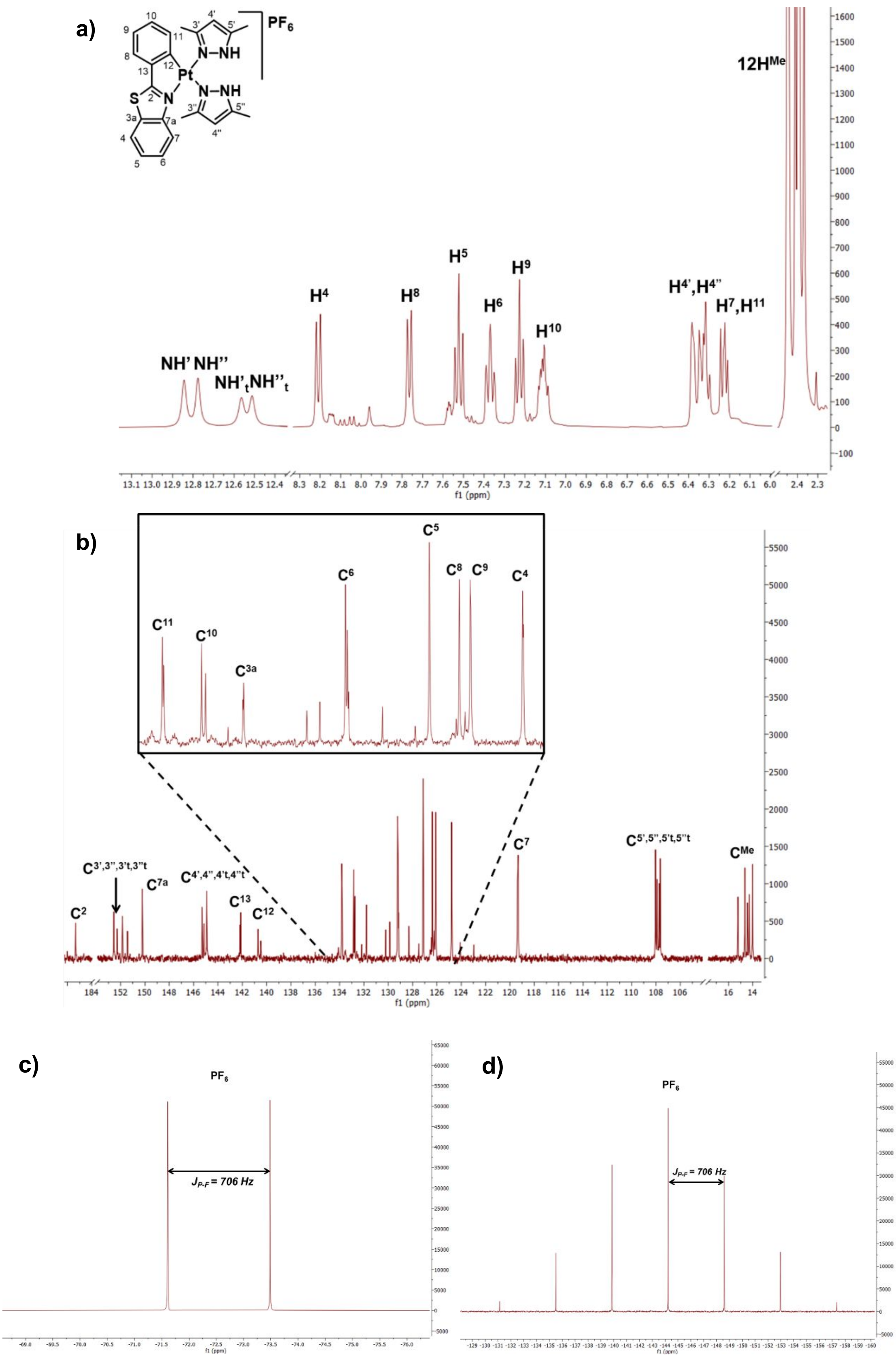

Figure S2. a) ^{1}H, b) $^{13}C\{^{1}H\}$, c) $^{19}F\{^{1}H\}$ and d) $^{31}P\{^{1}H\}$ NMR spectra of complex **1b** in CD_3COCD_3.

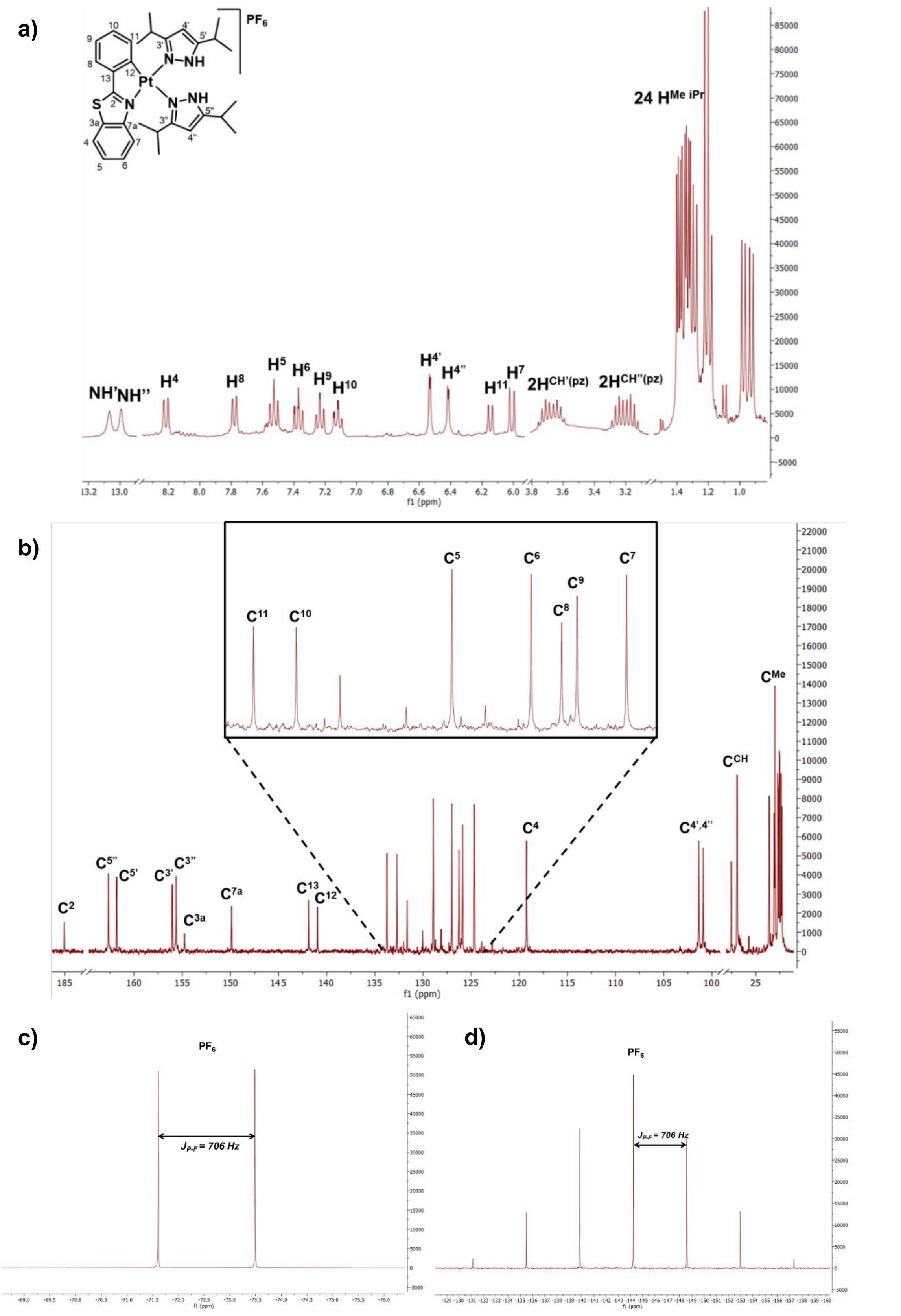

Figure S3. a) ^{1}H, b) $^{13}C\{^{1}H\}$, c) $^{19}F\{^{1}H\}$ and d) $^{31}P\{^{1}H\}$ NMR spectra of complex **1c** in CD_3COCD_3.

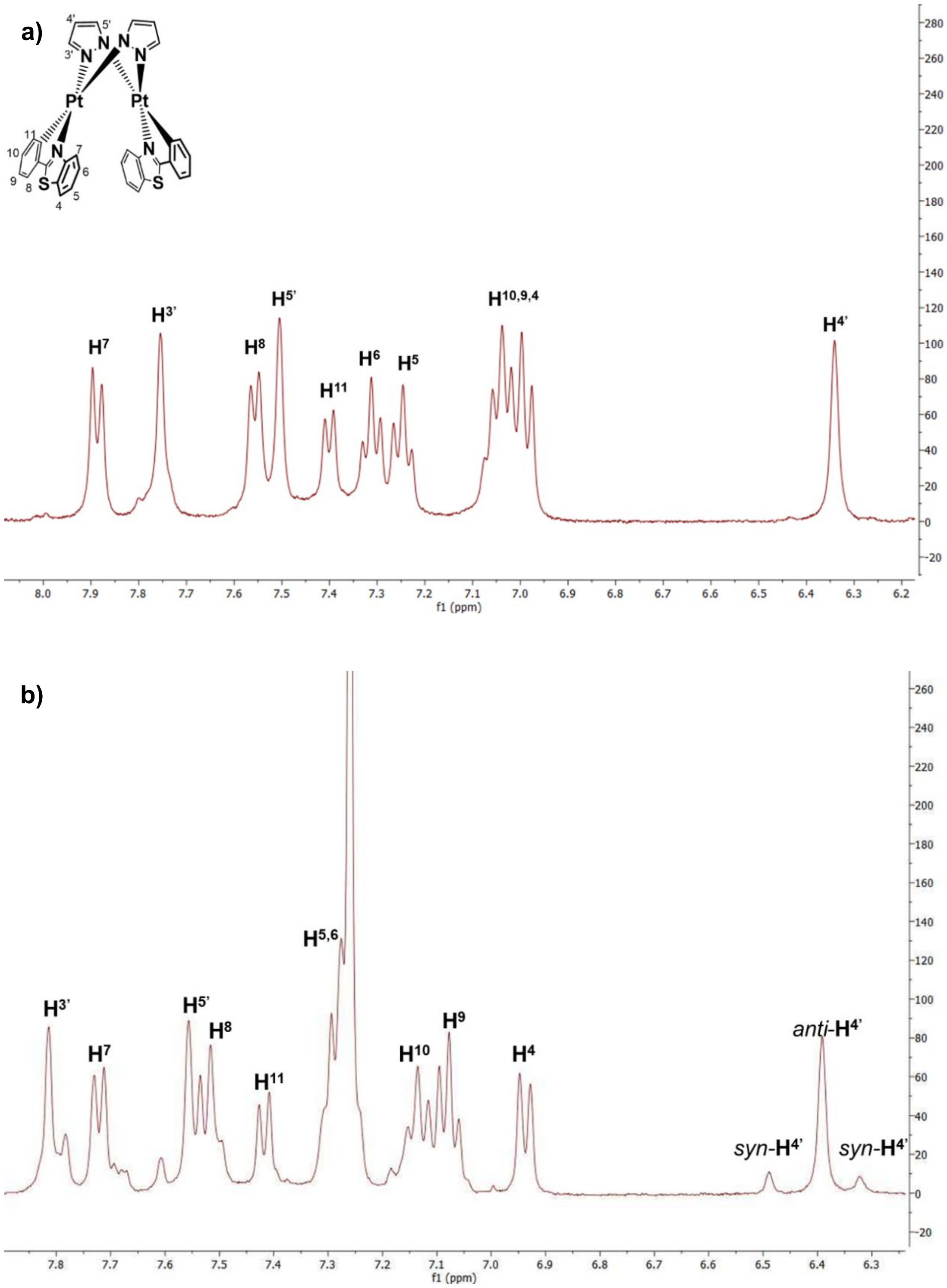

Figure S4. a) ^{1}H NMR spectra of complex **2a** in THF-d[8]; b) Mixture of *anti* and *syn* isomers (5:1 *anti/syn*) of **2a** obtained by procedure b (see Experimental).

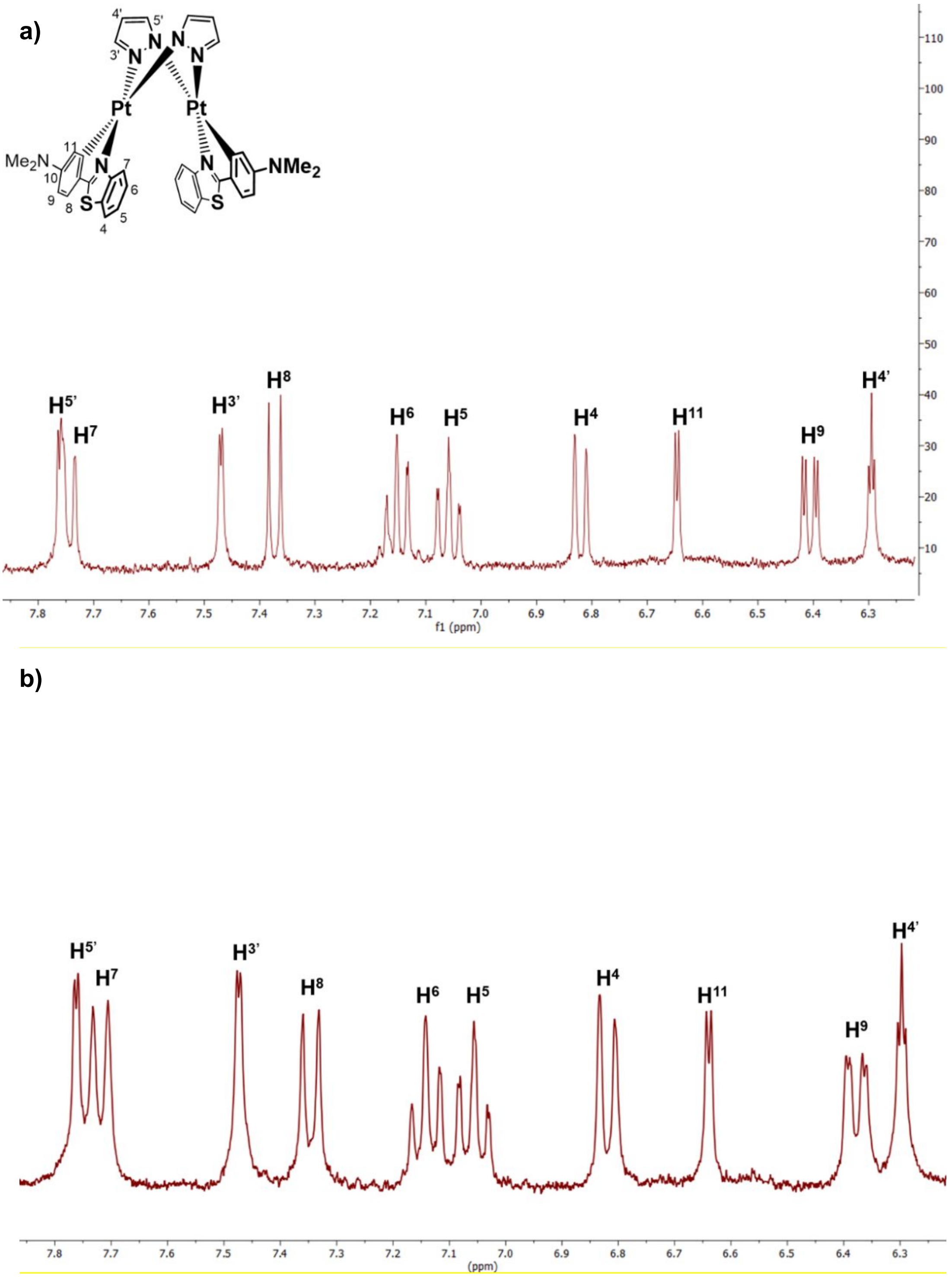

Figure S5. ^{1}H NMR spectra of complex **3a** in THF-d^{8} obtained by procedure a) a or b) b.

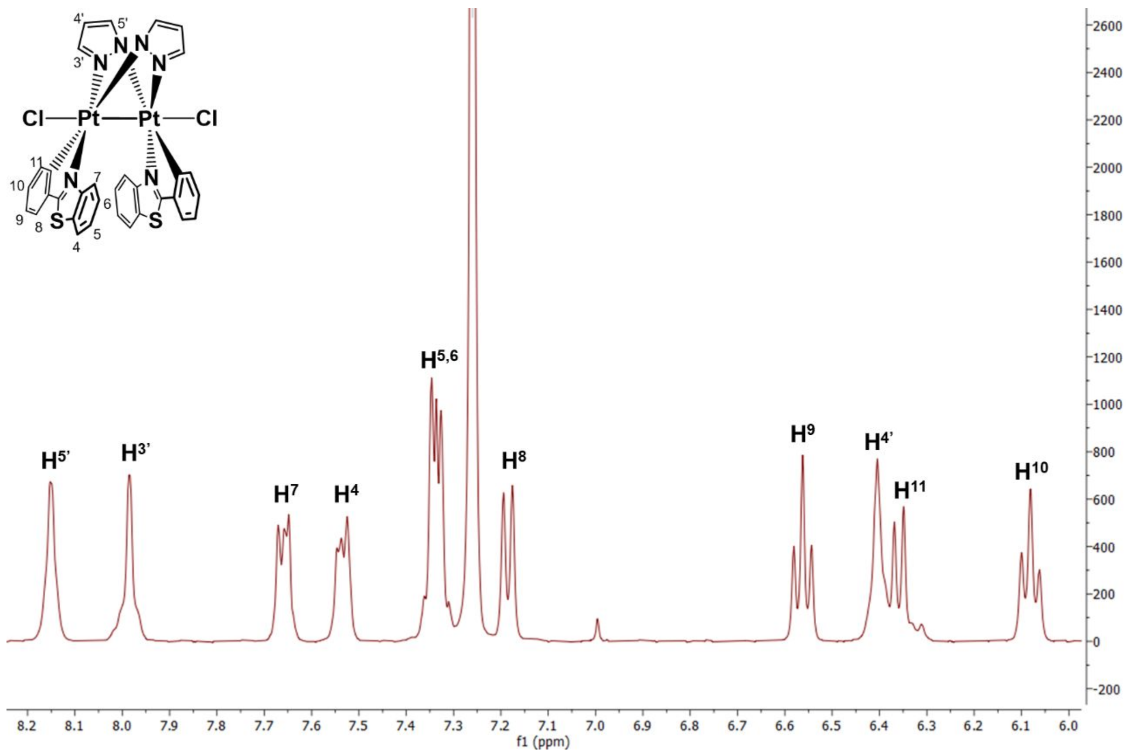

Figure S6. ^1H NMR spectra of complex **4a** in $CDCl_3$.

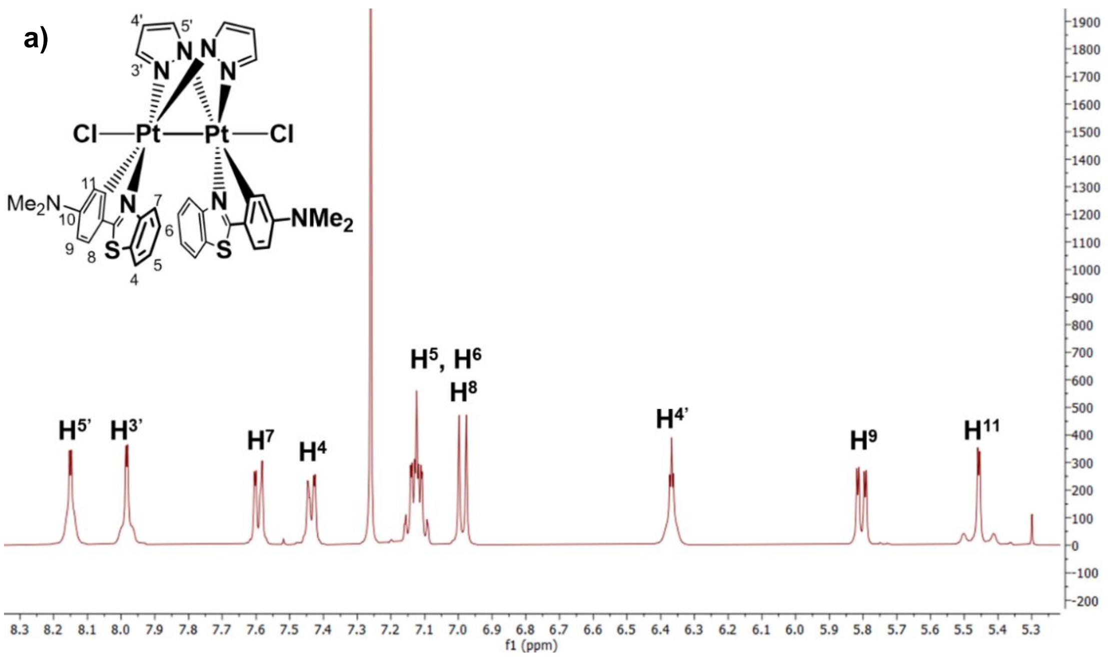

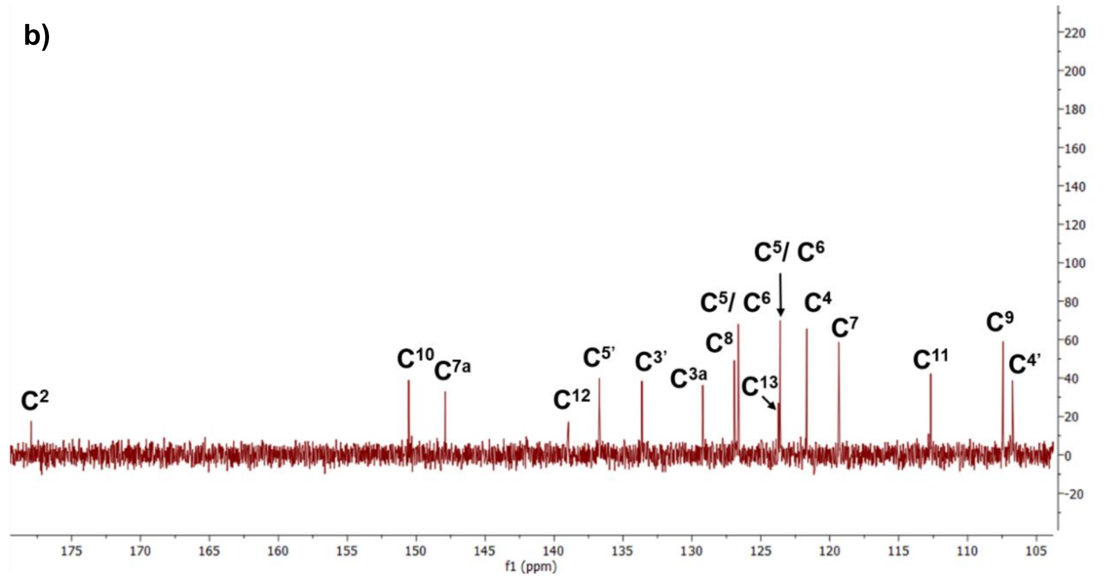

Figure S7. a) ^{1}H and b) $^{13}C\{^{1}H\}$ NMR spectra of complex **5a** in $CDCl_3$.

3.- Mass Spectra

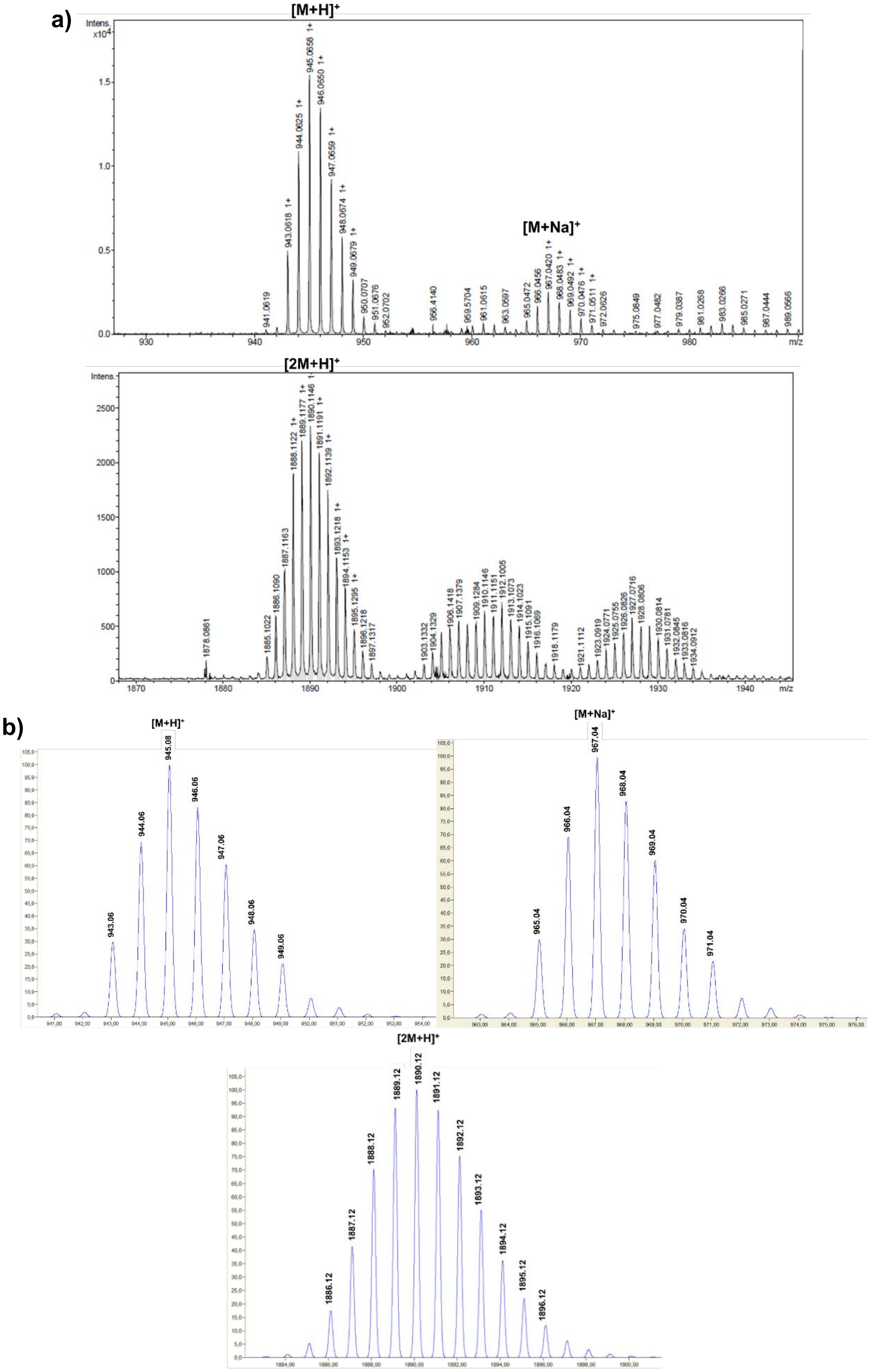

Figure S8. Mass Spectra in solution (ESI(+)) of complex **2a** a) Experimental and b) Simulated.

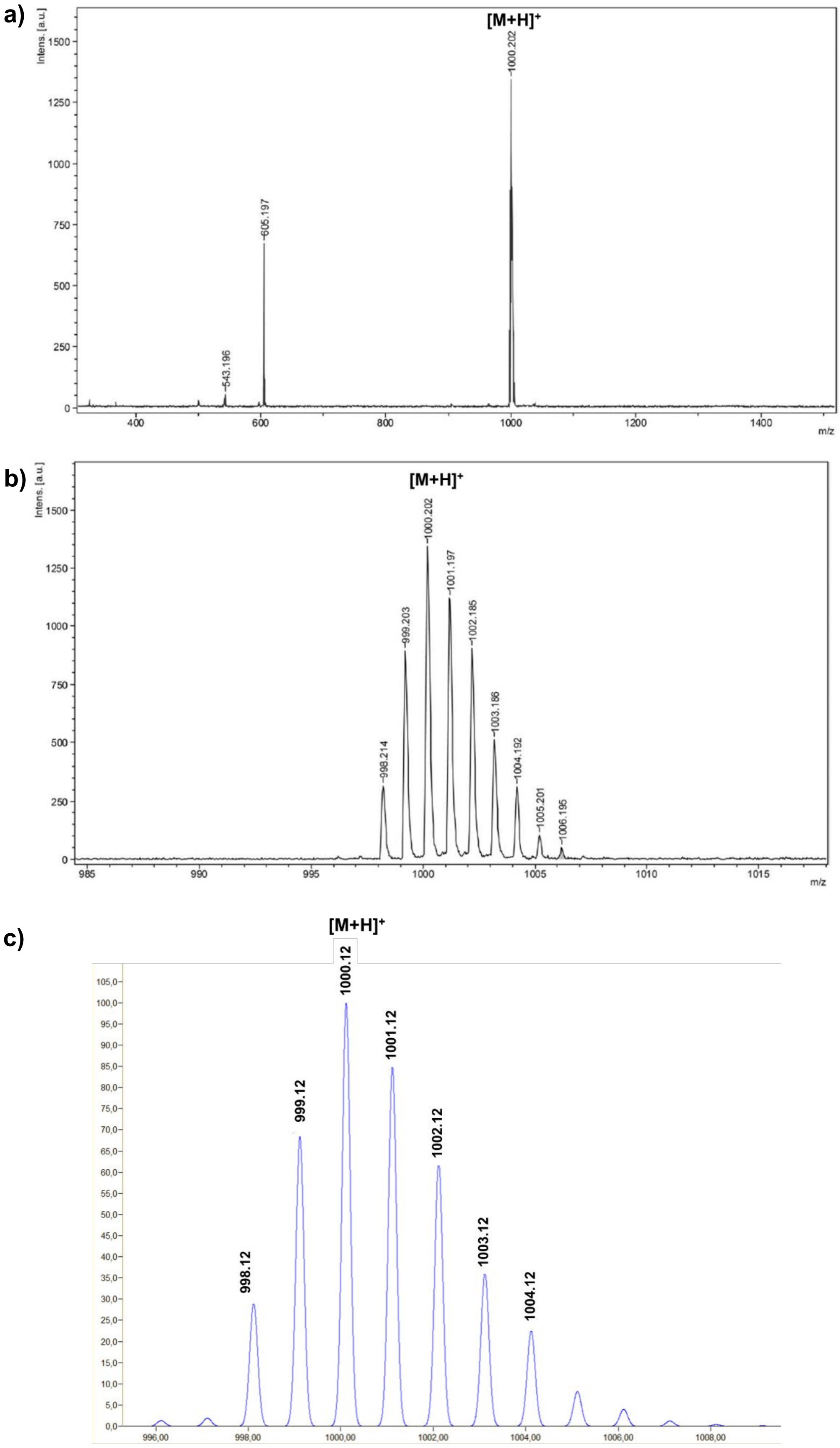

Figure S9. Mass Spectra in solid state (MALDI(+)) of complex **2b** a) Experimental, b) zoom of parent peak and c) Simulated.

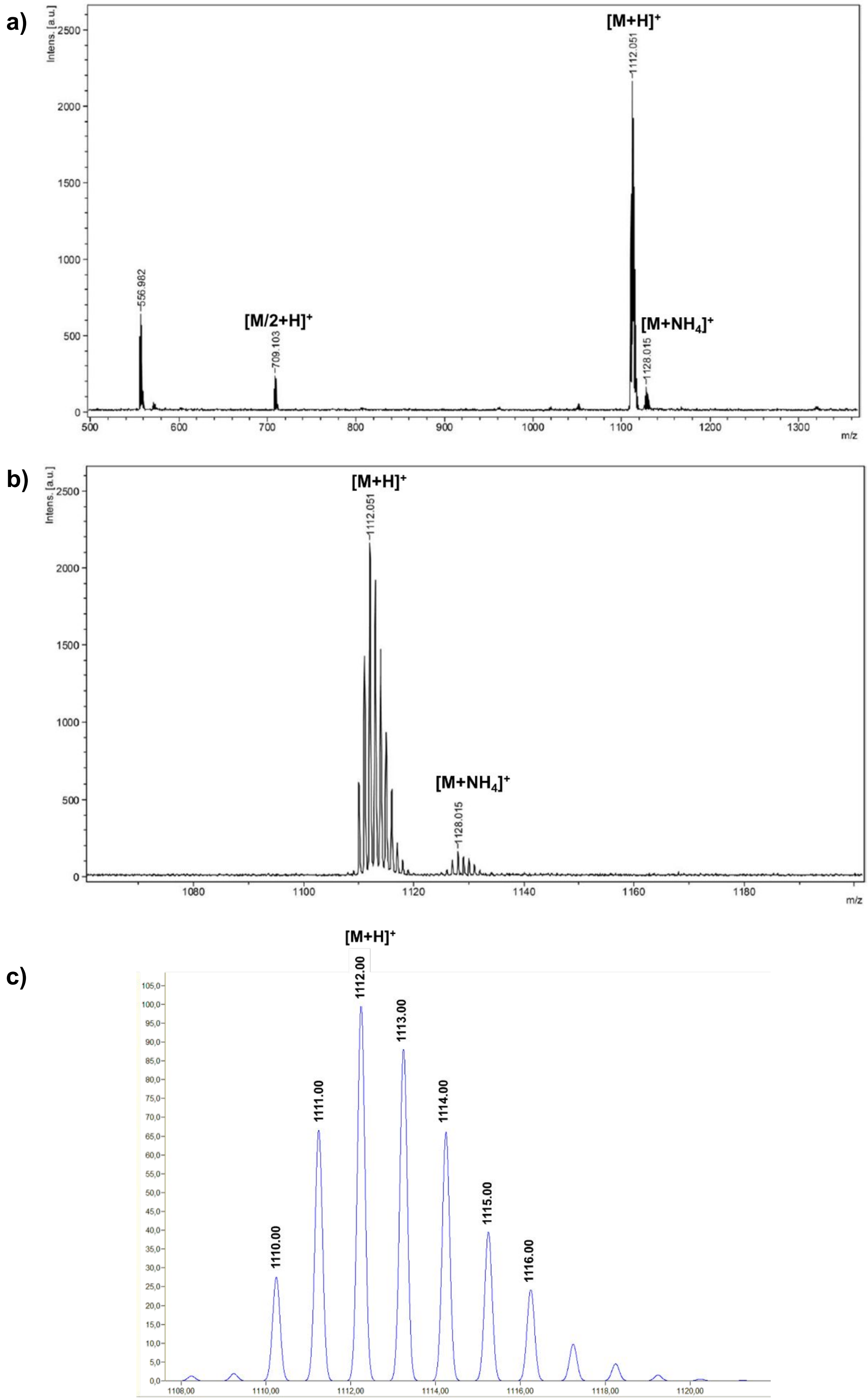

Figure S10. Mass Spectra in solid state (MALDI(+)) of complex **2c** a) Experimental, b) zoom of parent peak and c) Simulated.

4.- Crystal Structures

X-ray structure determinations. X-ray crystallographic data and selected bond lengths and angles for **1a·PO$_2$F$_2$**, **1b**, **2a**, **3a·THF**, **4a** and **5a·0.5CH$_2$Cl$_2$** are summarized in Tables S1 and S2. Yellow crystals of **1a·PO$_2$F$_2$** and **1b** were obtained by slow diffusion of *n*-hexane into a solution of the corresponding complex in acetone at low temperature, yellow crystals of **2a**, **3a·THF** and **4a** were obtained by slow evaporation solution of the complex in THF at room temperature and pale-yellow crystals of **5a·0.5CH$_2$Cl$_2$** were obtained by slow evaporation of a solution of the complex in CH_2Cl_2. X-ray intensity data were collected using Molybdenum graphite monochromatic (Mo-Kα) radiation with a Bruker APEX-II diffractometer at a temperature of 100 K with an Oxford Cryosystem temperature controller for **1b**, **2a**, **3a**, **4a** and **5a** and at 300 K for **1a** using the APEX-II software. Structures were solved by Intrinsic Phasing using SHELXT[3] with the WinGX graphical user interface.[4] Multi-scan absorption corrections were applied to all the data sets and refined by full-matrix least squares on F^2 with SHELXL.[5] hydrogen atoms were positioned geometrically, with isotropic parameters $U_{iso} = 1.2\ U_{eq}$ (parent atom) for aromatic hydrogens and CH_2 and $U_{iso} = 1.5\ U_{eq}$ (parent atom) for methyl groups. Finally, the structures show some residual peaks greater than 1 e Å^{-3} in the vicinity of the platinum atoms but with no chemical meaning. For **5a**, disordered crystallization molecules of solvents were observed, but could not be properly modelled. Examination with PLATON[6] and SQUEEZE[6-7] revealed the presence of two voids of 134 Å^3 in the unit cell, each of them containing 43 e$^-$, which are attributed to the presence of two molecules of CH_2Cl_2 (**5a·0.5CH$_2$Cl$_2$**).

Table S1. X-ray Crystallographic data for **1a·PO_2F_2**, **1b**, **2a**, **3a·THF**, **4a** and **5a·0.5CH_2Cl_2**

	**1a·PO_2F_2**	**1b**	**2a**
**Empirical formula**	$C_{19}H_{16}F_2N_5O_2PPt$ S	$C_{23}H_{24}F_6N_5PPtS$	$C_{32}H_{22}N_6Pt_2S_2$
**Molecular weight**	642.49	742.59	944.85
**T (K)**	301(2)	100(2)	100(2)
**Wavelength (Å)**	0.71076	0.71076	0.71076
**Crystal system**	Triclinic	Monoclinic	Triclinic
**Space group**	P-1	P 2_1/c	P -1
**Crystal size (mm)**	0.275 x 0.170 x 0.085	0.141 x 0.102 x 0.053	0.155 x 0.101 x 0.062
**a (Å)**	10.418(5)	14.1552(8)	10.9358(4)
**b (Å)**	10.573(4)	11.5293(5)	11.0309(5)
**c (Å)**	10.737(3)	15.7821(9)	13.0145(6)
**α (°)**	84.874(12)	90	106.853(2)
**β (°)**	83.616(13)	90.797(2)	103.858(2)
**γ (°)**	71.538(17)	90	93.385(2)
**V (Å^3)**	1113.0(8)	2575.4(2)	1444.78(11)
**Z**	2	4	2
**Density (calculated) (Mg/cm^3)**	1.917	1.915	2.172
**Absorption coefficient (mm^{-1})**	6.511	5.658	9.851
**F(000)**	616	1440	888
**θ range for data collection (°)**	2.871 to 24.713	2.278 to 27.924	2.959 to 27.918
**Index ranges**	-12<=h<=12, -12<=k<=12, -12<=l<=12	-18<=h<=18, -15<=k<=15, -20<=l<=20	-14<=h<=14, -14<=k<=14, -17<=l<=17
**Reflections collected**	46022	135779	79037
**Independent reflections**	3776 [R(int) = 0.0464]	6158 [R(int) = 0.0613]	6905 [R(int) = 0.0386]
**Data / restraints / parameters**	3776 / 6 / 257	6158 / 0 / 334	6905 / 0 / 379
**Goodness-of-fit on F^2**	1.143	1.121	1.066
**Final R indices [I>2σ(I)]**[a]	R1 = 0.0797, wR2 = 0.2199	R1 = 0.0696, wR2 = 0.1683	R1 = 0.0172, wR2 = 0.0439
**R indices (all data)**[a]	R1 = 0.0823, wR2 = 0.2228	R1 = 0.0756, wR2 = 0.1723	R1 = 0.0200, wR2 = 0.0452
**Largest diff. peak and hole (e Å^{-3}) (dmin/dmax)**	5.143 and -2.440	14.161 and -2.553	1.873 and -0.820

	3a·THF	4a	5a·0.5CH_2Cl_2
**Empirical formula**	$C_{40}H_{40}N_8OPt_2S_2$	$C_{32}H_{22}Cl_2N_6Pt_2S_2$	$C_{36}H_{32}Cl_2N_8Pt_2S_2$
**Molecular weight**	1103.10	1015.75	1101.89
**T (K)**	100(2)	100(2)	100(2)
**Wavelength (Å)**	0.71076	0.71076	0.71076
**Crystal system**	Monoclinic	Monoclinic	Monoclinic
**Space group**	$P2_1/n$	C 2/c	$P\ 2_1/c$
**Crystal size (mm)**	0.820 x 0.202 x 0.194	0.091 x 0.072 x 0.035	0.255 x 0.098 x 0.042
**a (Å)**	10.090(7)	18.6353(10)	18.303(8)
**b (Å)**	17.492(10)	9.8755(5)	11.972(5)
**c (Å)**	21.665(18)	16.9288(8)	19.253(8)
**α (°)**	90	90	90
**β (°)**	95.679(2)	99.883(2)	117.769(2)
**γ (°)**	90	90	90
**V ($Å^3$)**	3804.9(4)	3069.2(3)	4192.8(5)
**Z**	4	4	4
**Density (calculated) (Mg/cm^3)**	1.926	2.198	1.961
**Absorption coefficient (mm^{-1})**	7.500	9.451	7.781
**F(000)**	2128	1912	2104
**θ range for data collection (°)**	2.321 to 25.349	2.342 to 27.901	2.515 to 26.374
**Index ranges**	-12<=h<=12 -21<=k<=21, -26<=l<=26	-24<=h<=24, -12<=k<=12, -22<=l<=22	-22<=h<=22, -14<=k<=14, -24<=l<=24
**Reflections collected**	213669	96228	189537
**Independent reflections**	6952 [R(int) = 0.0503]	3661 [R(int) = 0.0479]	7620 [R(int) = 0.0610]
**Data / restraints / parameters**	6952 / 5 / 479	3661 / 0 / 199	7620 / 0 / 452
**Goodness-of-fit on F^2**	1.199	1.108	1.023
**Final R indices [I>2σ(I)]**[a]	R1 = 0.0301, wR2 = 0.0659	R1 = 0.0142, wR2 = 0.0291	R1 = 0.0340, wR2 = 0.0913
**R indices (all data)**[a]	R1 = 0.0354, wR2 = 0.0708	R1 = 0.0168, wR2 = 0.0300	R1 = 0.0444, wR2 = 0.0990
**Largest diff. peak and hole (e $Å^{-3}$) (dmin/dmax)**	2.233 and -1.293	1.198 and -0.856	5.588 and -0.936

Table S2. Selected distances (Å) and angles (º) for complexes **1a·PO$_2$F$_2$**, **1b**, **2a**, **3a·THF**, **4a** and **5a·0.5CH$_2$Cl$_2$**.

**1a·PO$_2$F$_2$**			
**Distances (Å)**		**Angles (°)**	
Pt(1)-N(1)	2.042(16)	C(1)-Pt(1)-N(4)	94.9(9)
Pt(1)-N(2)	2.08(2)	C(1)-Pt(1)-N(1)	80.4(9)
Pt(1)-N(4)	1.960(18)	N(1)-Pt(1)-N(2)	97.1(8)
Pt(1)-C(1)	2.01(2)	N(2)-Pt(1)-N(4)	87.7(8)
**1b**			
**Distances (Å)**		**Angles (°)**	
Pt(1)-N(1)	2.038(8)	C(1)-Pt(1)-N(4)	92.6(4)
Pt(1)-N(2)	2.117(9)	C(1)-Pt(1)-N(1)	80.8(4)
Pt(1)-N(4)	2.002(9)	N(1)-Pt(1)-N(2)	99.2(3)
Pt(1)-C(1)	2.007(10)	N(2)-Pt(1)-N(4)	87.4(3)
**2a**			
**Distances (Å)**		**Angles (°)**	
Pt(1)-N(1)	2.036(2)	C(1)-Pt(1)-N(4)	95.80(10)
Pt(1)-N(2)	2.099(2)	C(1)-Pt(1)-N(1)	80.76(10)
Pt(1)-N(4)	2.000(2)	N(1)-Pt(1)-N(2)	99.50(9)
Pt(1)-C(1)	2.001(3)	N(2)-Pt(1)-N(4)	84.16(9)
Pt(2)-N(6)	2.033(2)	C(20)-Pt(2)-N(3)	95.80(11)
Pt(2)-N(3)	1.989(2)	C(20)-Pt(2)-N(6)	80.92(11)
Pt(2)-N(5)	2.090(2)	N(6)-Pt(2)-N(5)	98.42(9)
Pt(2)-C(20)	1.998(3)	N(3)-Pt(2)-N(5)	84.72(9)
Pt(1)-Pt(2)	3.344		
**3a·THF**			
**Distances (Å)**		**Angles (°)**	
Pt(1)-N(1)	2.043 (5)	C(1)-Pt(1)-N(5)	95.0 (2)
Pt(1)-N(3)	2.103 (5)	C(1)-Pt(1)-N(1)	81.6 (2)
Pt(1)-N(5)	2.004 (5)	N(1)-Pt(1)-N(3)	98.7 (2)
Pt(1)-C(1)	2.001 (6)	N(3)-Pt(1)-N(5)	84.8 (2)
Pt(2)-N(7)	2.039 (5)	C(22)-Pt(2)-N(4)	95.0 (2)
Pt(2)-N(4)	1.998 (5)	C(22)-Pt(2)-N(7)	81.1 (2)
Pt(2)-N(6)	2.105 (5)	N(7)-Pt(2)-N(6)	99.0 (2)
Pt(2)-C(22)	1.993 (6)	N(4)-Pt(2)-N(6)	85.0 (2)
Pt(1)-Pt(2)	3.1740 (4)		

4a			
**Distances (Å)**		**Angles (°)**	
Pt(1)-N(1)	2.052(2)	C(1)-Pt(1)-N(3)	93.73(9)
Pt(1)-N(2)	2.118(2)	C(1)-Pt(1)-N(1)	80.83(9)
Pt(1)-N(3)	2.0038(19)	N(1)-Pt(1)-N(2)	99.73(8)
Pt(1)-C(1)	2.013(2)	N(2)-Pt(1)-N(3)	84.32(8)
Pt(1)-Cl(1)	2.4177(6)	Cl(1)-Pt(1)-Pt(1')	162.894(14)
Pt(1)-Pt(1')	2.58972(19)		
**5a·0.5CH_2Cl_2**			
**Distances (Å)**		**Angles (°)**	
Pt(1)-N(5)	2.003(6)	N(5)-Pt(1)-C(1)	91.4(2)
Pt(1)-C(1)	2.012(6)	C(1)-Pt(1)-N(1)	80.9(2)
Pt(1)-N(1)	2.055(5)	N(5)-Pt(1)-N(3)	84.0(2)
Pt(1)-N(3)	2.146(5)	N(1)-Pt(1)-N(3)	102.3(2)
Pt(1)-Cl(1)	2.4304(15)	N(4)-Pt(2)-C(22)	92.2(3)
Pt(2)-N(4)	2.001(6)	C(22)-Pt(2)-N(7)	80.8(3)
Pt(2)-C(22)	2.012(7)	N(4)-Pt(2)-N(6)	84.3(2)
Pt(2)-N(7)	2.049(6)	N(7)-Pt(2)-N(6)	100.8(2)
Pt(2)-N(6)	2.137(6)	Cl(2)-Pt(2)-Pt(1)	165.71(6)
Pt(2)-Cl(2)	2.4112(19)	Cl(1)-Pt(1)-Pt(2)	166.71(6)
Pt(1)-Pt(2)	2.5776(3)		

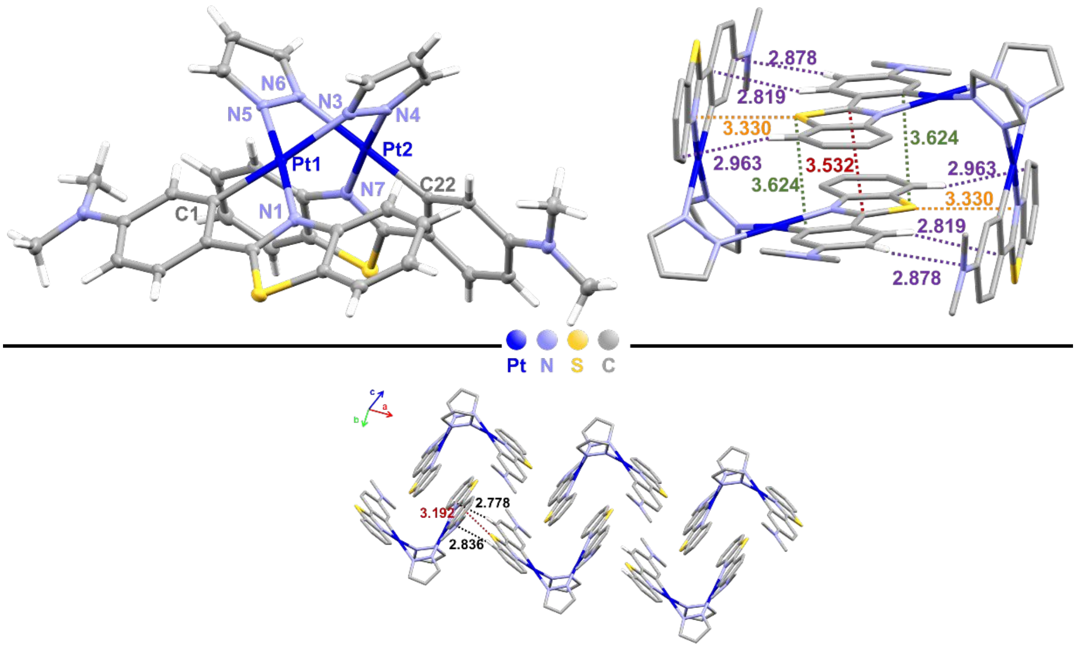

Figure S11. Crystal structure and packing of complex **3a·THF**.

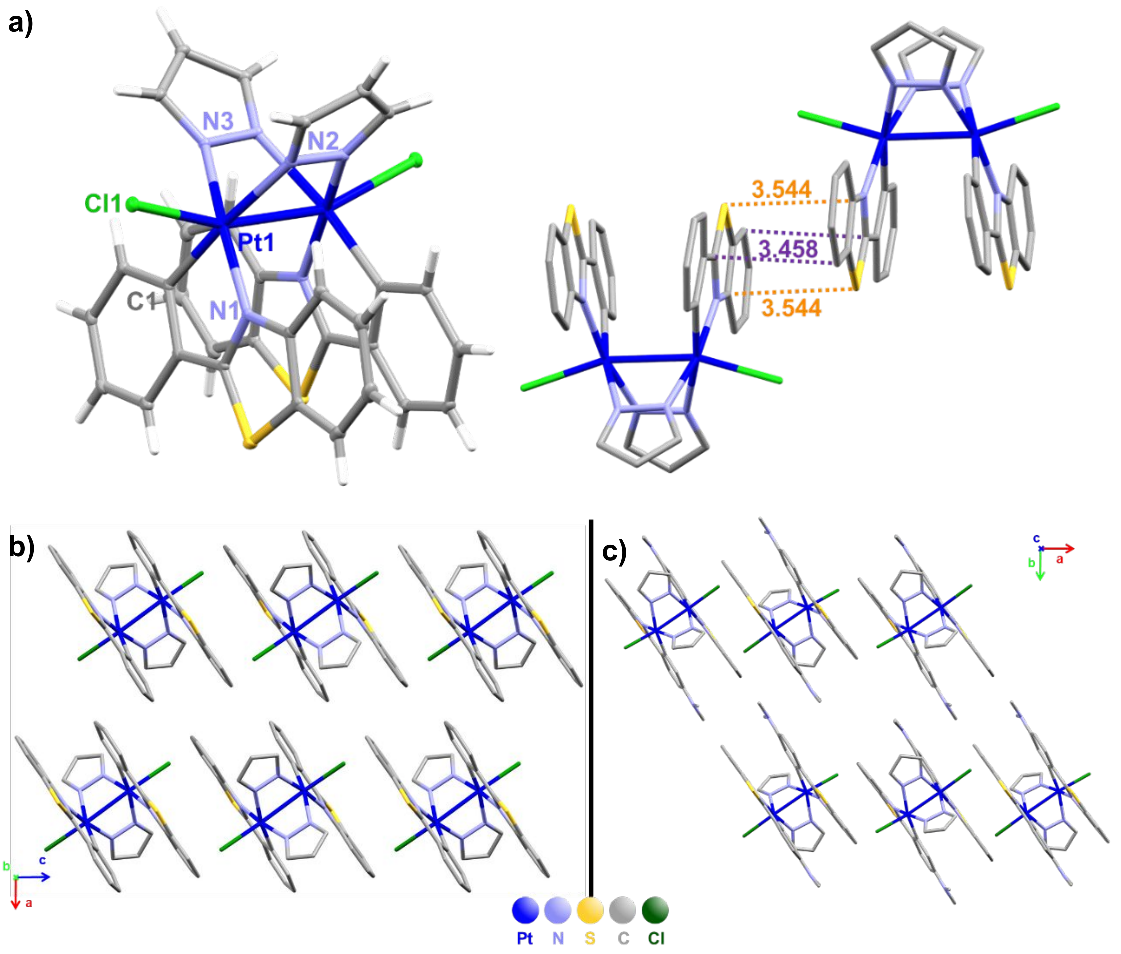

Figure S12. a) Molecular structure and crystal packing of **4a**. Packing of b) **4a** and c) **5a·0.5CH$_2$Cl$_2$**.

5.- Photophysical Properties and Theoretical Calculations

Table S3. UV-Vis absorption data for all complexes in THF solution (5 × 10^{-5} M) and solid state.

Complex	Media	λ_{abs}/nm (ε × 10^{-3}/M^{-1}cm^{-1})
**[Pt(pbt)(pzH)$_2$]PF$_6$ (1a)**	**THF**	258 (16.10), 317 (10.97), 329 (11.68), 354 (5.45), 386 (3.86), 411 (2.79)
	**Solid**	330, 400, 410 tail to 440
**[Pt(pbt)(3,5-Me$_2$pzH)$_2$]PF$_6$ (1b)**	**THF**	257 (19.87), 319 (13.77), 330 (13.68), 355 (5.84), 390 (4.16), 413 (13.23)
	**Solid**	332, 396, 412 tail to 450
**[Pt(pbt)(3,5-^{i}Pr$_2$pzH)$_2$]PF$_6$ (1c)**	**THF**	257 (17.93), 319 (12.67),329 (12.21), 355 (5.35), 390 (3.65), 413 (2.87)
	**Solid**	327, 393, 410 tail to 445
**[Pt(pbt)(μ-pz)]$_2$ (2a)**	**THF**	265 (36.52), 313(22.65), 323 (25.29), 357 (13.43), 384 (13.07), 440 (4.78) tail to 465
	**Solid**	336, 404, 438, 493, 528
**[Pt(pbt)(μ-3,5-Me$_2$pz)]$_2$ (2b)**	**Solid**	332, 420, 455, 497, 528
**[Pt(pbt)(μ-3,5-^{i}Pr$_2$pz)]$_2$ (2c)**	**Solid**	332, 404, 470, 503, 529
**[Pt(Me$_2$N-pbt)(μ-pz)]$_2$ (3a)**	**THF**	258 (82.16), 306 (11.32), 366 (25.61), 430 (24.07), 450 (20.13) tail to 475
	**Solid**	300, 374, 430 tail to 505
**[Pt(pbt)(μ-pz)Cl]$_2$ (4a)**	**THF**	266 (8.54), 314 (12.70), 348 (10.90), 379$_{sh}$ (4.80)
	**Solid**	365 tail to 518
**[Pt(Me$_2$N-pbt)(μ-pz)Cl]$_2$ (5a)**	**THF**	276 (5.36), 327 (4.26), 370 (6.29), 383 (10.15), 402 (16.41), 444 (2.66)
	**Solid**	295, 352, 408 tail to 570

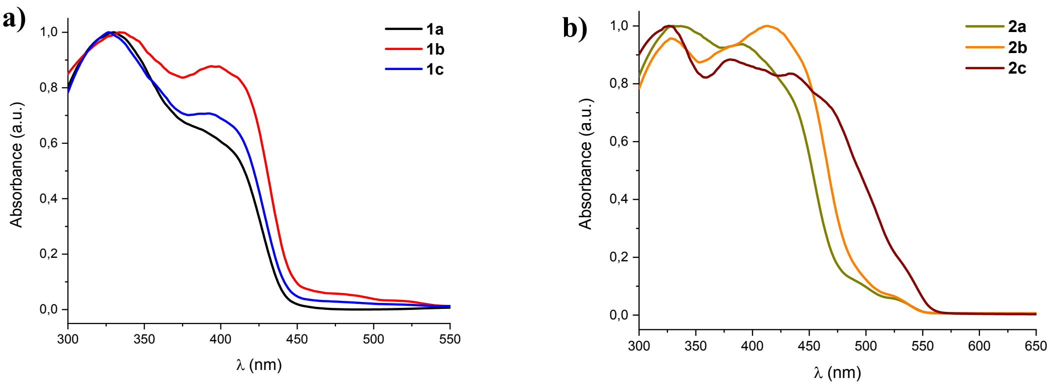

Figure S13. UV-Vis spectra of complexes a) **1**, b) **2** in solid state.

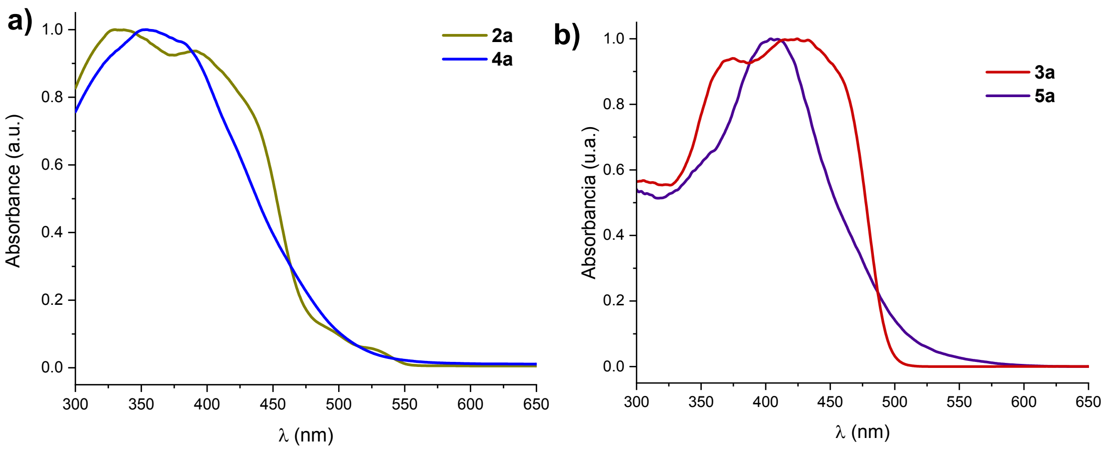

Figure S14. UV-Vis spectra of complexes a) **2a**, **4a** and b) **3a**, **5a** in solid state.

Theoretical calculations

Calculations were carried out with the Gaussian 16 package[8] for **1a**$^+$-**5a**, using Becke´s three-parameter functional combined with Lee-Yang-Parr´s correlation functional (B3LYP)[9] with the Becke-Johnson D3BJ correction.[10] Optimizations on the singlet state (S_0) were performed using as a starting point the molecular geometry obtained through X-ray diffraction analysis for all complexes. No negative frequency was found in the vibrational frequency analysis of the final equilibrium geometries. The basis set used was the LanL2DZ effective core potential for Pt and 6-31G(d,p) for the ligand atoms.[11] DFT and TD-DFT calculations were carried out using the polarized continuum model approach[12] (PCM) implemented in the Gaussian 16 software, in presence of THF. The predicted emission wavelengths were obtained by energy difference between the triplet state at its optimized geometry and singlet state at the triplet geometry. The results were visualized with GaussView 6. Overlap populations between molecular fragments were calculated using the GaussSum 3.0 software.[13]

Table S4. Selected vertical excitations energies singlets (S_n) and first triplets (T_n) computed by TD-DFT/SCRF (THF Solution) with the orbitals involved.

Complex	State	λ/nm	*f*	Transition (% Contribution)	Character
$1a^+$	T_1	496.5	-	HOMO→LUMO (88%)	IL/MLCT
	T_2	388.6	-	H-1→LUMO (83%)	IL/MLCT
	T_3	374.4	-	H-2→LUMO (79%)	IL/MLCT
	S_1	382.3	0.1411	HOMO→LUMO (94%)	IL/MLCT
	S_2	338.4	0.0078	H-3→LUMO (80%), H-2→LUMO (12%)	MLCT/IL
	S_3	323.4	0.1553	H-1→LUMO (84%)	IL/MLCT
	S_4	314.5	0.2497	H-3→LUMO (10%), H-2→LUMO (82%)	IL/MLCT
	S_5	300.0	0.0128	H-4→LUMO (93%)	MLCT/L'LCT
	S_6	280.0	0.0049	HOMO→L+1 (13%), HOMO→L+2 (12%), HOMO→L+3 (52%)	IL/LMCT/LL'CT
	S_7	276.5	0.0908	H-5→LUMO (78%)	MLCT/L'LCT
	S_8	271.1	0.0068	H-3→L+1 (11%), H-3→L+2 (12%), H-3→L+3 (47%)	MLCT/ML'CT/MC
2a	T_1	508.0	-	H-2→LUMO (29%), H-1→L+1 (34%), HOMO→LUMO (22%)	IL/MLCT
	T_2	506.2	-	H-2→L+1 (28%), H-1→LUMO (44%), HOMO→L+1 (13%)	IL/MLCT
	T_3	464.6	-	H-2→LUMO (14%), HOMO→LUMO (72%)	MMLCT/IL
	S_1	452.2	0.0648	HOMO→LUMO (97%)	MMLCT
	S_2	438.6	0.0026	HOMO→L+1 (96%)	MMLCT
	S_3	400.2	0.0067	H-2→L+1 (31%), H-1→LUMO (64%)	IL/MLCT
	S_4	396.6	0.1132	H-2→LUMO (60%), H-1→L+1 (34%)	IL/MLCT
	S_5	368.2	0.0243	H-3→LUMO (11%), H-2→LUMO (35%), H-1→L+1 (51%)	IL/MLCT
	S_6	367.3	0.0007	H-2→L+1 (63%), H-1→LUMO (32%)	IL/MMLCT
	S_7	364.4	0.0587	H-4→L+1 (17%), H-3→LUMO (67%), H-1→L+1 (11%)	L'LCT/IL/MLCT
	S_8	361.6	0.0703	H-4→LUMO (68%), H-3→L+1 (25%)	L'LCT
	S_9	349.3	0.0143	H-4→LUMO (27%), H-3→L+1 (71%)	L'LCT /IL
	S_{10}	348.1	0.0041	H-4→L+1 (79%), H-3→LUMO (19%)	L'LCT /IL
3a	T_1	556.4	-	H-1→L+1 (41%), HOMO→LUMO (55%)	ILCT
	T_2	556.3	-	H-1→LUMO (53%), HOMO→L+1 (43%)	ILCT
	T_3	437.5	-	H-2→LUMO (95%)	MMLCT
	S_1	423.1	0	HOMO→LUMO (90%)	ILCT
	S_2	422.6	0.1137	H-2→LUMO (85%), H-1→LUMO (12%)	MMLCT/ILCT
	S_3	414.5	0.3175	H-2→LUMO (11%), H-1→LUMO (83%)	ILCT/MMLCT
	S_4	405.3	0.0007	H-2→L+1 (47%), H-1→L+1 (45%)	ILCT/MMLCT
	S_5	400.7	0.0008	H-2→L+1 (49%), H-1→L+1 (47%)	ILCT/MMLCT
	S_6	399.1	0.6484	HOMO→L+1 (89%)	LC
	S_7	351.3	0.2087	H-4→LUMO (65%), H-3→L+1 (26%)	LC/MLCT
	S_8	351.0	0.0277	H-4→L+1 (24%), H-3→LUMO (67%)	ILCT/MLCT
	S_9	338.5	0.2220	H-5→LUMO (84%)	L'LCT/MLCT/ILCT

**4a**	**T_1**	624.9	-	H-2→LUMO (63%), HOMO→LUMO (24%)	LMMCT
	**T_2**	517.7	-	H-7→LUMO (22%), H-2→LUMO (13%), HOMO→LUMO (48%)	LMMCT/LXCT
	**T_3**	487.5	-	H-2→L+1 (10%), H-1→L+2 (15%), HOMO→L+1 (55%)	IL/XLCT/MLCT
	**S_1**	488.6	0.0038	HOMO→LUMO (85%)	LMMCT/LXCT
	**S_2**	455.3	0.0001	H-1→LUMO (91%)	LMMCT/LXCT
	**S_3**	430.7	0.0004	H-6→LUMO (37%), H-3→LUMO (48%)	L'MMCT/L'XCT
	**S_4**	428.0	0.0138	H-4→LUMO (76%), H-2→LUMO (18%)	L'MMCT/L'XCT
	**S_5**	415.5	0.0274	H-9→LUMO (10%), H-7→LUMO (18%), H-4→LUMO (18%), H-2→LUMO (40%)	LMMCT/XMMCT/ L'MMCT
	**S_6**	413.3	0.0009	H-8→LUMO (14%), H-6→LUMO (44%), H-3→LUMO (37%)	LMMCT/XMMCT/ L'MMCT
	**S_7**	401.0	0	H-8→LUMO (79%), H-3→LUMO (14%)	L'MMCT
	**S_8**	399.9	0.0544	H-12→LUMO (19%), H-7→LUMO (42%), H-2→LUMO (17%), HOMO→LUMO (10%)	LMMCT/XMMCT/
	**S_9**	386.0	0.0063	H-10→LUMO (19%), H-9→LUMO (75%)	L'MMCT/LMMCT/ XMMCT
	**S_{10}**	384.1	0.0074	H-13→LUMO (14%), H-5→LUMO (38%), HOMO→L+1 (43%)	IL/XLMMCT/ LMMCT/LXCT
	**S_{11}**	382.1	0.0104	H-5→LUMO (40%), HOMO→L+1 (52%)	IL/LMMCT/LXCT
	**S_{12}**	373.6	0.0216	H-14→LUMO (68%), H-10→LUMO (20%)	XMMCT/L'MMCT/ LMMCT
**5a**	**T_1**	762.8	-	HOMO→LUMO (97%)	LMMCT/LXCT
	**T_2**	630.4	-	H-2→LUMO (86%)	LMMCT/ MC
	**T_3**	624.9	-	H-1→LUMO (93%)	LMMCT/LXCT
	**T_4**	603.0	-	HOMO→L+1 (83%)	ILCT
	**T_5**	548.6	-	H-1→L+1 (57%), HOMO→L+2 (39%)	ILCT
	**S_1**	748.1	0.0004	HOMO→LUMO (99%)	LMMCT/LXCT
	**S_2**	619.6	0.0001	H-1→LUMO (99%)	LMMCT/LXCT
	**S_3**	488.9	0.0002	HOMO→L+1 (99%)	ILCT
	**S_4**	447.0	0.0245	H-10→LUMO (17%), H-6→LUMO (21%), H-5→LUMO (24%), H-2→LUMO (32%)	LMMCT/XC/MC/ L'MCT
	**S_5**	438.2	0.0007	H-8→LUMO (15%), H-7→LUMO (14%), H-4→LUMO (26%), H-3→LUMO (39%)	LMMCT/L'MCT/ XMCT
	**S_6**	430.1	0.0698	H-1→L+1 (72%), HOMO→L+2 (27%)	ILCT
	**S_7**	424.7	0.006	H-6→LUMO (33%), H-5→LUMO (60%)	LMMCT/L'MCT
	**S_8**	423.0	0	H-4→LUMO (37%), H-3→LUMO (46%)	LMMCT/L'MCT
	**S_9**	409.9	0.0003	H-9→LUMO (27%), H-7→LUMO (41%), H-4→LUMO (12%)	XMMCT/LMMCT/ L'MCT
	**S_{10}**	404.3	0.0735	H-16→LUMO (10%), H-14→LUMO (10%), H-6→LUMO (32%), H-2→LUMO (41%)	LMMCT/IX/MC /L'MMCT
	**S_{11}**	397.6	0.0001	H-9→LUMO (65%), H-8→LUMO (10%), H-4→LUMO (17%)	L'MMCT/L'XCT/ LXCT
	**S_{12}**	383.5	0.8533	H-11→LUMO (19%), H-1→L+1 (18%), HOMO→L+2 (51%)	ILCT

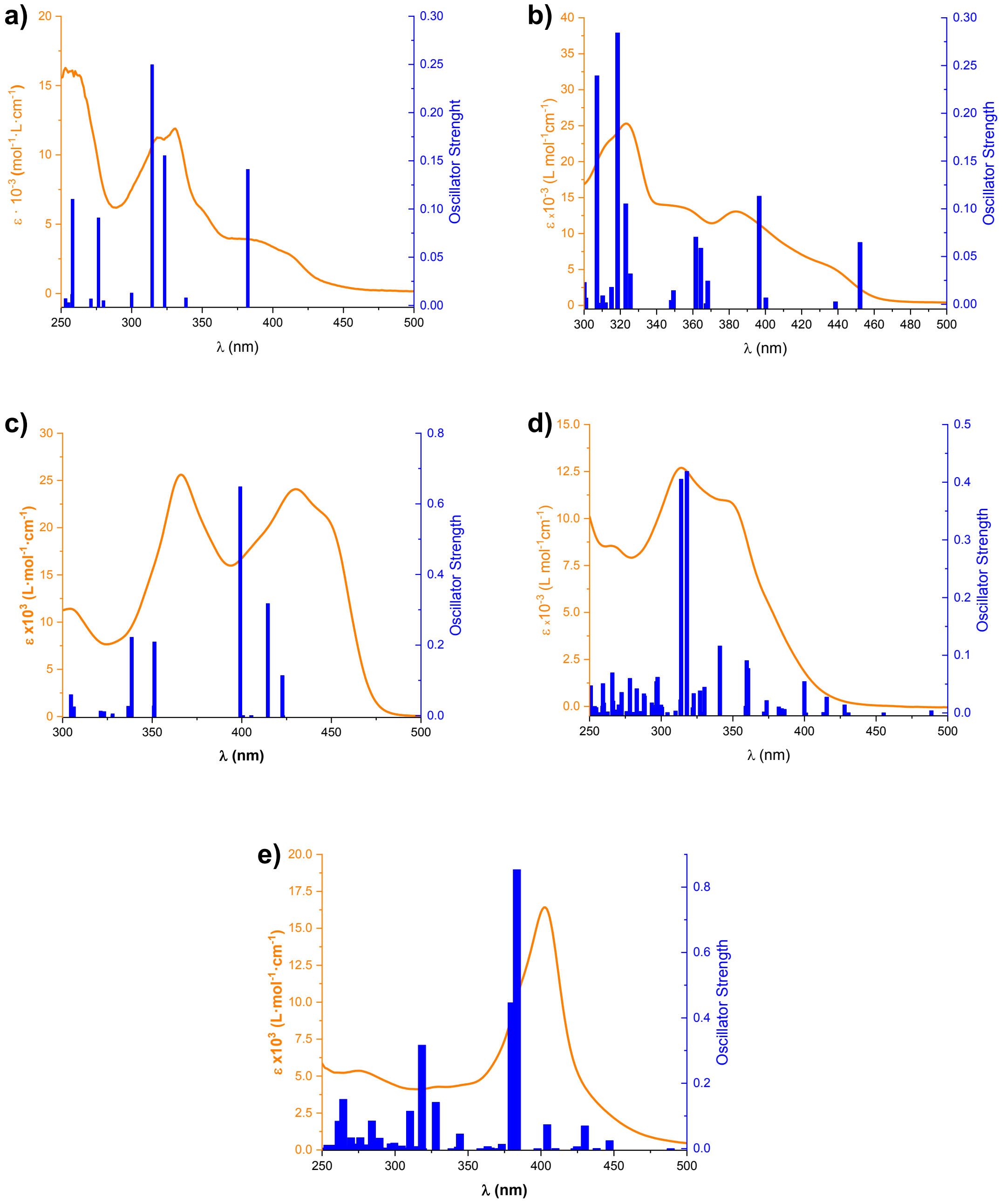

Figure S15. Stacked experimental (orange line) and calculated (blue bars) UV-Vis spectra for complexes a) **1a**$^+$, b) **2a**, c) **3a**, d) **4a** and e) **5a**.

Table S5. Selected parameter of DFT optimized geometries for ground state and triplet state in THF solution (PCM model)

$1a^+$			
**Parameter**	**X-Ray**	**S_0**	**T_1**
Pt(1) - C(1)	2.01(2)	2.00505	1.99400
Pt(1) - N(1)	2.042(16)	2.06932	2.02950
Pt(1) - N(2)	2.08(2)	2.17953	2.18713
Pt(1) - N(4)	1.960(18)	2.03674	2.04529
C(1)-Pt(1)-N(1)	80.4(9)	80.78235	82.12979
C(1)-Pt(1)-N(4)	94.9(9)	93.84912	93.42842
N(1)-Pt(1)-N(2)	97.1(8)	99.34388	98.83978
N(2)-Pt(1)-N(4)	87.7(8)	86.01655	85.87329
**2a**			
**Parameter**	**X-Ray**	**S_0**	**T_1**
Pt(1)-N(1)	2.036(2)	2.07067	2.07057
Pt(1)-N(2)	2.099(2)	2.16753	2.16458
Pt(1)-N(4)	2.000(2)	2.02401	2.02312
Pt(1)-C(1)	2.001(3)	2.00276	2.00528
Pt(2)-N(6)	2.033(2)	2.07067	2.00623
Pt(2)-N(3)	1.989(2)	2.02403	2.02962
Pt(2)-N(5)	2.090(2)	2.16756	2.17224
Pt(2)-C(20)	1.998(3)	2.00276	1.99982
Pt(1)-Pt(2)	3.344	3.20686	3.25105
C(1)-Pt(1)-N(4)	95.80(10)	95.86369	95.94242
C(1)-Pt(1)-N(1)	80.76(10)	80.45125	80.48564
N(1)-Pt(1)-N(2)	99.50(9)	99.93774	99.81392
N(2)-Pt(1)-N(4)	84.16(9)	83.86906	83.93656
C(20)-Pt(2)-N(3)	95.80(11)	95.86299	95.65388
C(20)-Pt(2)-N(6)	80.92(11)	80.45105	81.94075
N(6)-Pt(2)-N(5)	98.42(9)	99.93935	98.06394
N(3)-Pt(2)-N(5)	84.72(9)	83.86798	84.61133

3a			
**Parameter**	**X-Ray**	**S_0**	**T_1**
Pt(1)-N(1)	2.043 (5)	2.07786	2.07894
Pt(1)-N(3)	2.103 (5)	2.17034	2.16753
Pt(1)-N(5)	2.004 (5)	2.02851	2.02846
Pt(1)-C(1)	2.001 (6)	2.00269	2.00323
Pt(2)-N(7)	2.039 (5)	2.07786	2.04205
Pt(2)-N(4)	1.998 (5)	2.02851	2.03455
Pt(2)-N(6)	2.105 (5)	2.17034	2.17155
Pt(2)-C(22)	1.993 (6)	2.00269	2.00025
Pt(1)-Pt(2)	3.1740 (4)	3.19374	3.21792
C(1)-Pt(1)-N(5)	95.0 (2)	95.42290	95.49145
C(1)-Pt(1)-N(1)	81.6 (2)	80.61412	80.64236
N(1)-Pt(1)-N(3)	98.7 (2)	100.40893	100.33473
N(3)-Pt(1)-N(5)	84.8 (2)	83.51038	83.48350
C(22)-Pt(2)-N(4)	95.0 (2)	95.42292	94.93335
C(22)-Pt(2)-N(7)	81.1 (2)	80.61410	81.56433
N(7)-Pt(2)-N(6)	99.0 (2)	100.40895	99.78260
N(4)-Pt(2)-N(6)	85.0 (2)	83.51035	83.70389

4a			
**Parameter**	**X-Ray**	**S_0**	**T_1**
Pt(1)-N(1)	2.052(2)	2.07674	2.09394
Pt(1)-N(2)	2.118(2)	2.20526	2.22993
Pt(1)-N(3)	2.0038(19)	2.01974	2.04549
Pt(1)-C(1)	2.013(2)	2.01624	2.00891
Pt(1)-Cl(1)	2.4177(6)	2.49658	2.59749
Pt(1)-Pt(1’)	2.58972(19)	2.64527	3.07376
C(1)-Pt(1)-N(3)	93.73(9)	93.71923	94.84851
C(1)-Pt(1)-N(1)	80.83(9)	80.43227	80.72085
N(1)-Pt(1)-N(2)	99.73(8)	101.15117	99.29857
N(2)-Pt(1)-N(3)	84.32(8)	82.91234	82.04269
Cl(1)-Pt(1)-Pt(1’)	162.894(14)	165.40602	149.77925

5a			
**Parameter**	**X-Ray**	**S_0**	**T_1**
Pt(1)-N(5)	2.003(6)	2.02557	2.03336
Pt(1)-C(1)	2.012(6)	2.01886	1.99882
Pt(1)-N(1)	2.055(5)	2.07573	2.08278
Pt(1)-N(3)	2.146(5)	2.20816	2.19473
Pt(1)-Cl(1)	2.4304(15)	2.50437	2.78717
Pt(2)-N(4)	2.001(6)	2.02557	2.03337
Pt(2)-C(22)	2.012(7)	2.01883	1.99884
Pt(2)-N(7)	2.049(6)	2.07576	2.08279
Pt(2)-N(6)	2.137(6)	2.20812	2.19469
Pt(2)-Cl(2)	2.4112(19)	2.50439	2.78711
Pt(1)-Pt(2)	2.5776(3)	2.64538	2.76858
N(5)-Pt(1)-C(1)	91.4(2)	93.33459	93.25695
C(1)-Pt(1)-N(1)	80.9(2)	80.50118	80.42846
N(5)-Pt(1)-N(3)	84.0(2)	83.05597	83.03475
N(1)-Pt(1)-N(3)	102.3(2)	101.36159	100.49104
N(4)-Pt(2)-C(22)	92.2(3)	93.33411	93.26275
C(22)-Pt(2)-N(7)	80.8(3)	80.49977	80.42665
N(4)-Pt(2)-N(6)	84.3(2)	83.05711	83.03366
N(7)-Pt(2)-N(6)	100.8(2)	101.36142	100.48712
Cl(2)-Pt(2)-Pt(1)	165.71(6)	165.15819	160.96161

Table S6. DFT optimized geometries for ground state and triplet state (in THF solution)

$1a^+$	
**Singlet (S_0)**	**Triplet (T_1)**
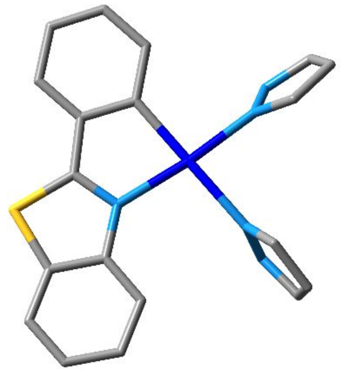	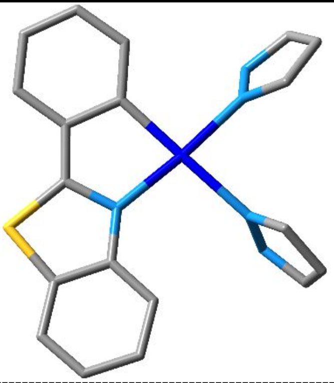
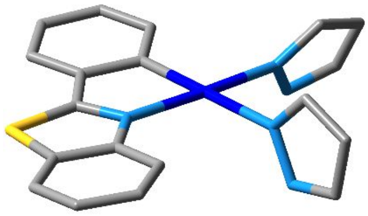	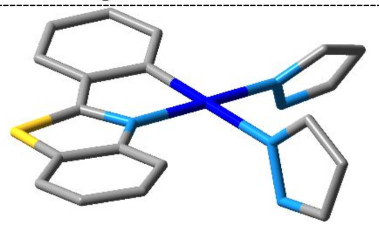

**2a**	
**Singlet (S_0)**	**Triplet (T_1)**
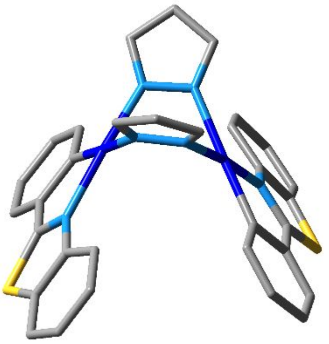	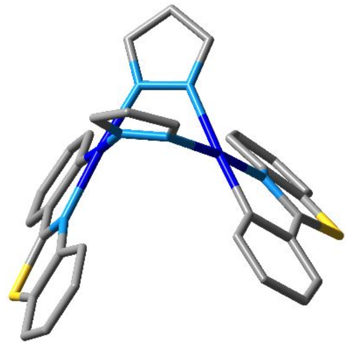
**3a**	
**Singlet (S_0)**	**Triplet (T_1)**
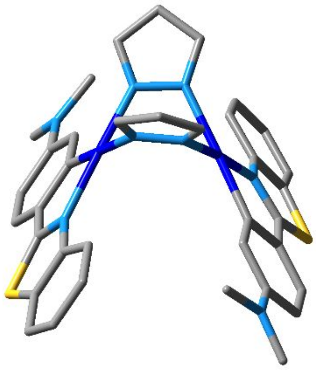	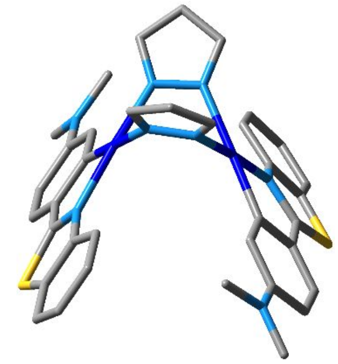
**4a**	
**Singlet (S_0)**	**Triplet (T_1)**
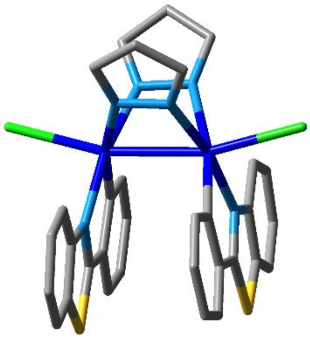	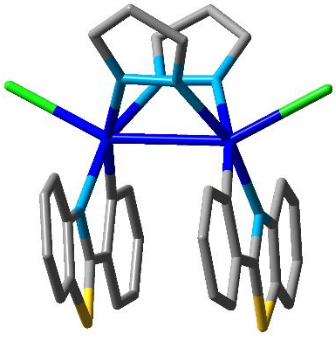

5a	
Singlet (S_0)	Triplet (T_1)
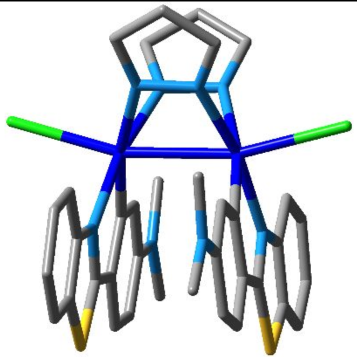	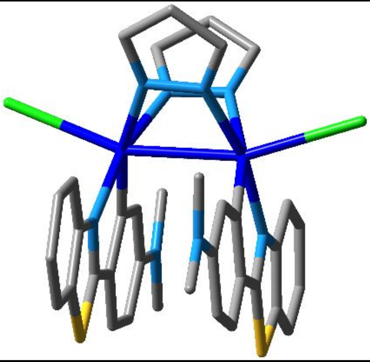

a)

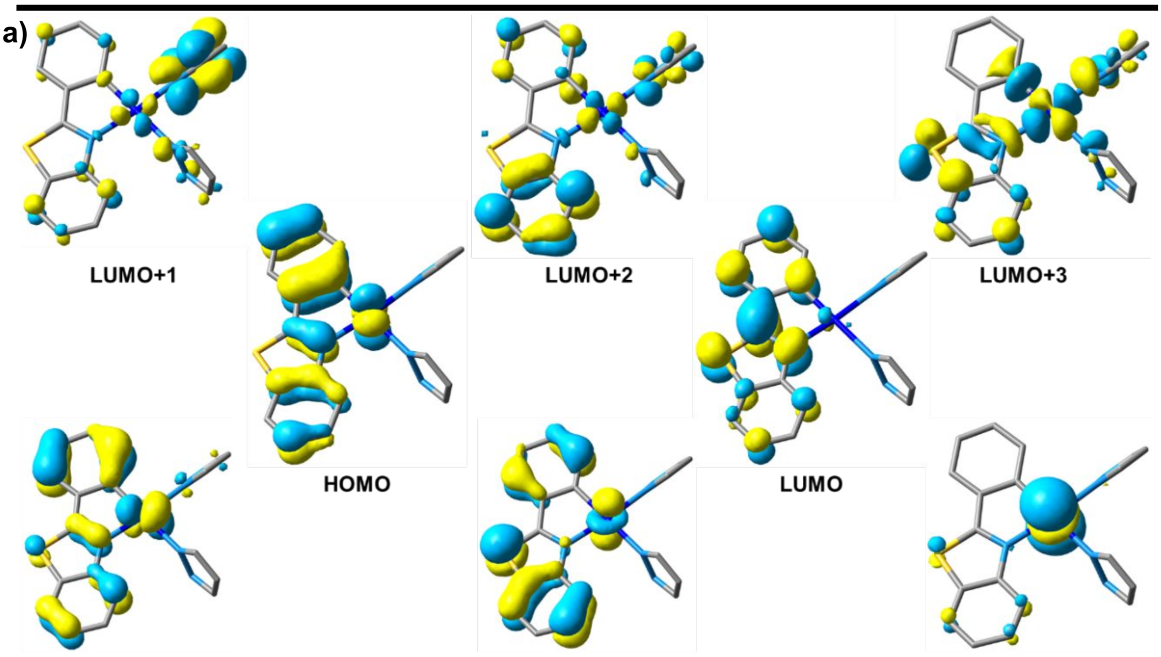

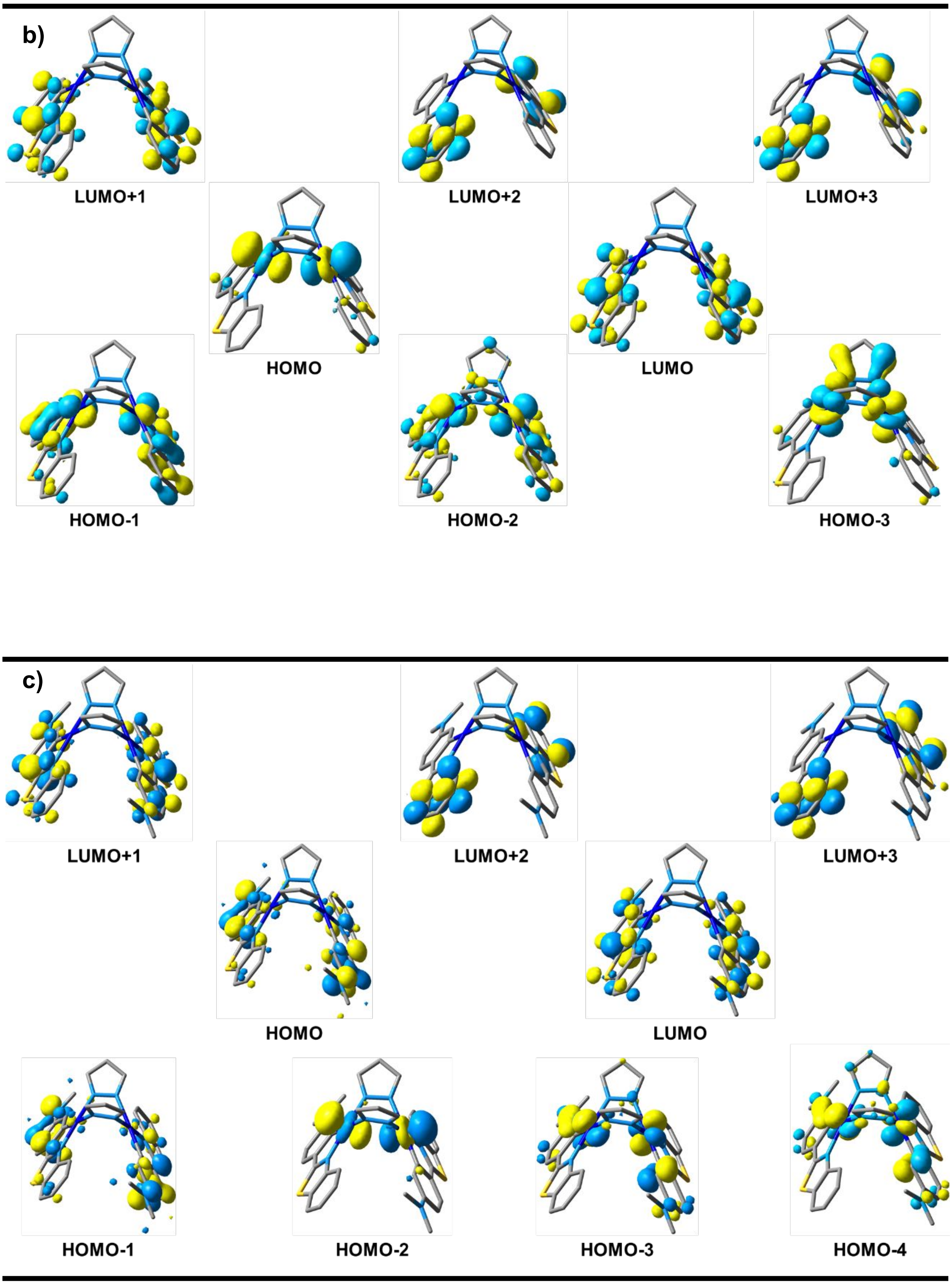
b)
LUMO+1
LUMO+2
LUMO+3
HOMO
LUMO
HOMO-1
HOMO-2
HOMO-3
c)
LUMO+1
LUMO+2
LUMO+3
HOMO
LUMO
HOMO-1
HOMO-2
HOMO-3
HOMO-4

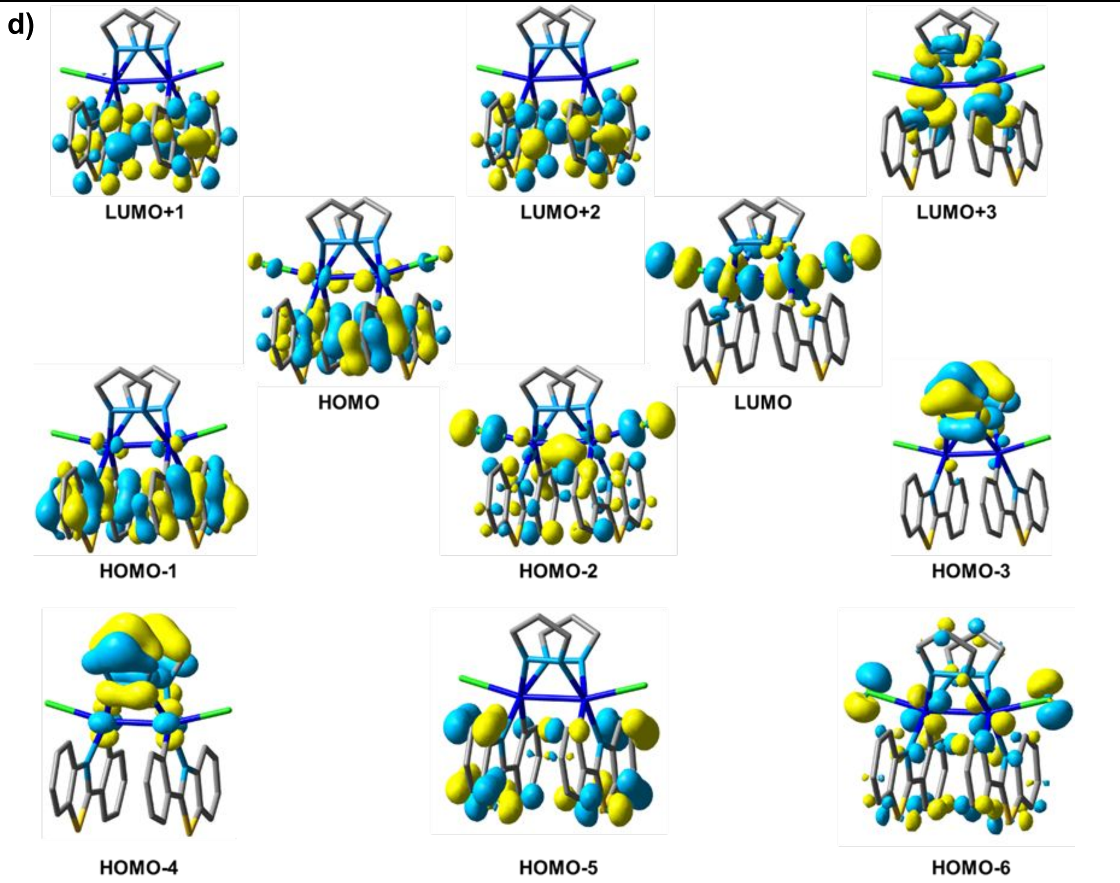
d)
LUMO+1
LUMO+2
LUMO+3
HOMO
LUMO
HOMO-1
HOMO-2
HOMO-3
HOMO-4
HOMO-5
HOMO-6

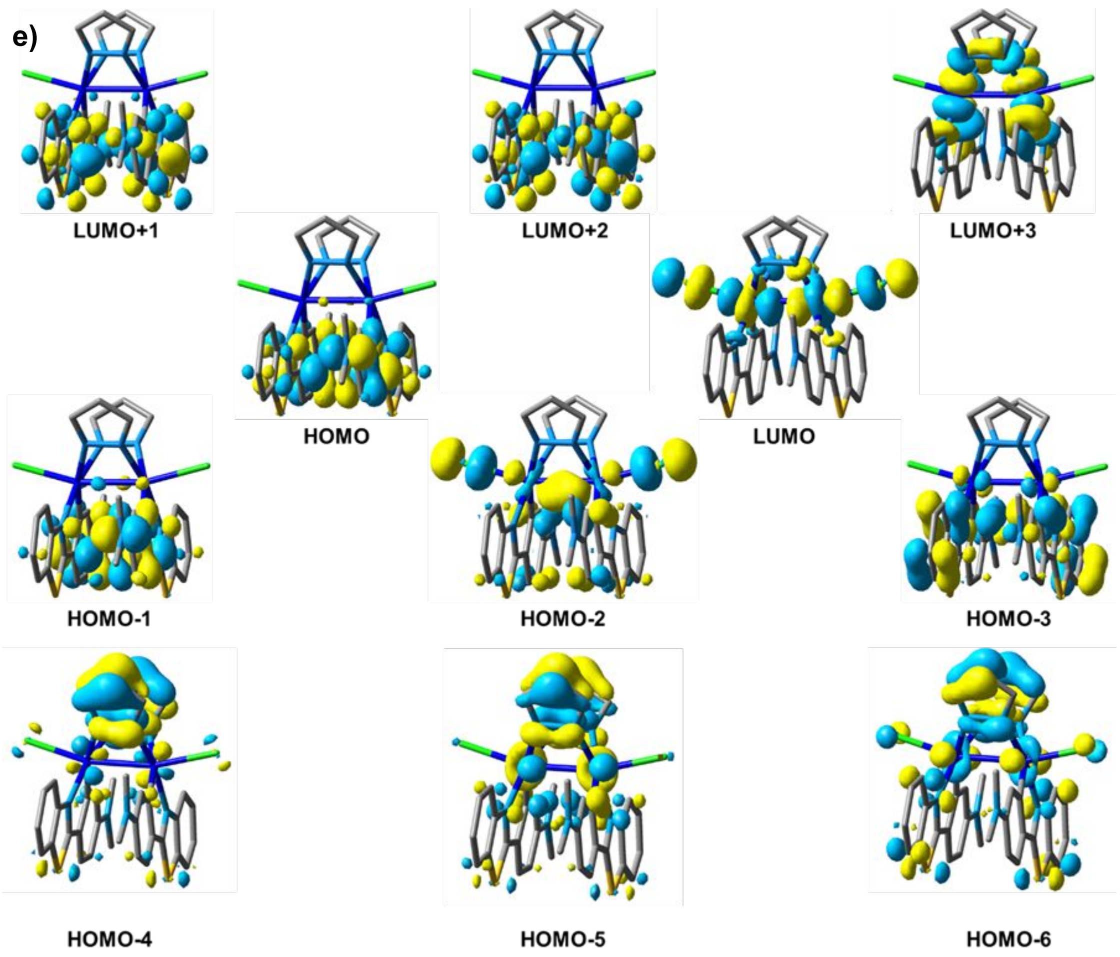

Figure S16. Selected frontier Molecular Orbitals for a) **1a**$^+$, b) **2a**, c) **3a**, d) **4a** and e) **5a** in the ground state in THF solution.

Table S7. Composition (%) of Frontier MOs in terms of ligands and metals in the ground state in solution.

.

**$1a^{+}$**					
**Orbital**	**Energy (eV)**	**Pt**	**pbt**	**pzH(1)**	**pzH(2)**
**LUMO+5**	-0.78	2	4	7	88
**LUMO+4**	-0.79	11	78	9	1
**LUMO+3**	-0.82	34	45	15	5
**LUMO+2**	-0.9	24	59	14	3
**LUMO+1**	-1.00	11	19	63	7
**LUMO**	-2.52	6	93	1	0
**HOMO**	-6.46	20	80	0	0
**HOMO-1**	-6.94	29	66	4	1
**HOMO-2**	-7.04	17	81	1	0
**HOMO-3**	-7.07	84	14	2	1
**HOMO-4**	-7.38	35	18	29	19
**HOMO-5**	-7.72	31	18	10	41

**2a**							
**Orbital**	**Energy (eV)**	**Pt(1)**	**Pt(2)**	**pbt(1)**	**pbt(2)**	**pz(1)**	**pz(2)**
**LUMO+5**	-0.15	10	10	36	36	4	4
**LUMO+4**	-0.31	9	9	41	41	0	0
**LUMO+3**	-0.41	1	1	49	49	0	0
**LUMO+2**	-0.45	4	4	45	45	0	0
**LUMO+1**	-1.94	2	2	48	48	0	0
**LUMO**	-2.00	3	3	46	46	0	0
**HOMO**	-5.46	40	40	8	8	2	2
**HOMO-1**	-5.81	17	17	31	31	2	2
**HOMO-2**	-5.83	19	19	24	24	6	6
**HOMO-3**	-6.02	21	21	11	11	18	18
**HOMO-4**	-6.05	18	18	7	7	25	25
**HOMO-5**	-6.38	2	2	27	26	22	21

**3a**							
**Orbital**	**Energy (eV)**	**Pt(1)**	**Pt(2)**	**Me_2N-pbt(1)**	**Me_2N-pbt(2)**	**pz(1)**	**pz(2)**
**LUMO+5**	-0.02	12	12	33	33	5	5
**LUMO+4**	-0.17	12	12	38	38	0	0
**LUMO+3**	-0.3	1	1	49	49	0	0
**LUMO+2**	-0.34	3	3	47	47	0	0
**LUMO+1**	-1.56	1	1	48	48	0	0
**LUMO**	-1.67	3	3	46	46	0	0
**HOMO**	-5.06	2	2	48	48	0	0
**HOMO-1**	-5.06	2	2	48	48	0	0
**HOMO-2**	-5.31	44	44	4	4	2	2
**HOMO-3**	-5.88	22	22	24	24	4	4
**HOMO-4**	-5.88	22	22	22	22	6	6
**HOMO-5**	-5.91	19	19	12	12	20	20

4a									
**Orbital**	**Energy (eV)**	**Pt(1)**	**Pt(2)**	**pbt(1)**	**pbt(2)**	**pz(1)**	**pz(2)**	**Cl(1)**	**Cl(2)**
**LUMO+5**	-0.64	1	1	49	49	0	0	0	0
**LUMO+4**	-0.85	21	21	20	20	10	10	0	0
**LUMO+3**	-1.20	22	22	19	19	9	9	0	0
**LUMO+2**	-2.02	1	1	49	49	0	0	0	0
**LUMO+1**	-2.43	1	1	48	48	0	0	1	1
**LUMO**	-2.97	29	29	4	4	4	4	13	13
**HOMO**	-6.28	4	4	42	42	0	0	3	3
**HOMO-1**	-6.44	2	2	47	47	0	0	0	0
**HOMO-2**	-6.44	8	8	27	26	1	1	14	14
**HOMO-3**	-6.73	2	2	4	4	43	43	1	1
**HOMO-4**	-6.76	7	7	3	3	40	40	0	0
**HOMO-5**	-6.80	1	1	49	49	1	1	0	0

5a									
**Orbital**	**Energy (eV)**	**Pt(1)**	**Pt(2)**	**Me_2N-pbt(1)**	**Me_2N-pbt(2)**	**pz(1)**	**pz(2)**	**Cl(1)**	**Cl(2)**
**LUMO+5**	-0.46	1	1	49	49	0	0	0	0
**LUMO+4**	-0.65	21	21	20	20	9	9	0	0
**LUMO+3**	-1.03	22	22	19	19	9	9	0	0
**LUMO+2**	-1.56	1	1	49	49	0	0	0	0
**LUMO+1**	-2.06	1	1	48	48	0	0	0	0
**LUMO**	-2.84	29	29	4	4	4	4	13	13
**HOMO**	-5.12	0	0	49	49	0	0	0	0
**HOMO-1**	-5.45	0	0	50	50	0	0	0	0
**HOMO-2**	-6.27	11	11	23	23	1	1	16	16
**HOMO-3**	-6.49	3	3	47	47	0	0	0	0
**HOMO-4**	-6.64	4	4	14	14	30	31	2	2
**HOMO-5**	-6.64	7	7	20	20	21	20	2	2
**HOMO-6**	-6.68	7	7	19	19	20	20	3	3

Table S8. Emission data for all complexes in solid state and PS film (10% *wt*) (λ_{ex} 365 – 450 nm)

Complex	Media	T^a / K	λ_{em}/nm	τ/μs	ϕ	$k_r{}^a$/s^{-1}	$k_{nr}{}^b$/s^{-1}
**$[Pt(pbt)(pzH)_2]PF_6$ (1a)**	Solid	298	532, 573_{max}, 623	10.6	0.01	9.4×10^2	9.3×10^4
		77	548, 587_{max}, 625	-	-		
	PS	298	540, 575_{max}, 622	11.7	0.04	3.4×10^3	8.2×10^4
**$[Pt(pbt)(3,5\text{-}Me_2pzH)_2]PF_6$ (1b)**	Solid	298	539, 577_{max}, 624	12.3	0.06	4.9×10^3	7.6×10^4
		77	538, 581_{max}, 631	-	-		
	PS	298	539, 575_{max},620	11.6	0.08	6.9×10^3	7.9×10^4
**$[Pt(pbt)(3,5\text{-}^iPr_2pzH)_2]PF_6$ (1c)**	Solid	298	544, 577_{max}, 624	9.9	0.08	8.1×10^3	9.3×10^4
		77	538, 580_{max}, 632	-	-		
	PS	298	545, 574_{max}, 622	10.9	0.09	8.3×10^3	8.3×10^4
**$[Pt(pbt)(\mu\text{-}pz)]_2$ (2a)**	Solid	298	553, 589_{max}, 635	14.6	0.04	2.7×10^3	6.6×10^4
		77	554, 598_{max}, 644	-	-		
	PS	298	546, 587_{max}, 630	10.4	0.09	8.7×10^3	8.8×10^4
**$[Pt(pbt)(\mu\text{-}3,5\text{-}Me_2pz)]_2$ (2b)**	Solid	298	549, 585_{max}, 633	2.6 (49%), 18.7 (51%)	0.08	7.4×10^3	8.5×10^4
		77	551, 591_{max}, 631	-	-		
**$[Pt(pbt)(\mu\text{-}3,5\text{-}^iPr_2pz)]_2$ (2c)**	Solid	298	560, 594_{max}, 647	3.1 (42%), 11.5 (58%)	0.04	5.0×10^3	1.2×10^5
		77	601	-	-		
**$[Pt(Me_2N\text{-}pbt)(\mu\text{-}pz)]_2$ (3a)**	Solid	298	570, 615_{max}	12.0	0.01	8.3×10^2	8.3×10^4
		77	600, 631_{max}	-	-		
	PS	298	570_{max}, 609	15.0	<0.01	6.7×10^2	6.6×10^4
**$[Pt(pbt)(\mu\text{-}pz)Cl]_2$ (4a)**	PS	298	550, 584_{max}	9.7 [550]	0.02	2.1×10^3	1.0×10^5
**$[Pt(Me_2N\text{-}pbt)(\mu\text{-}pz)Cl]_2$ (5a)**	PS	298	566, 606_{max}	16.3	<0.01	6.1×10^2	6.1×10^4

a) $k_r = \phi/\tau_{average}$. b) $k_{nr} = (1 - \phi)/\tau_{average}$

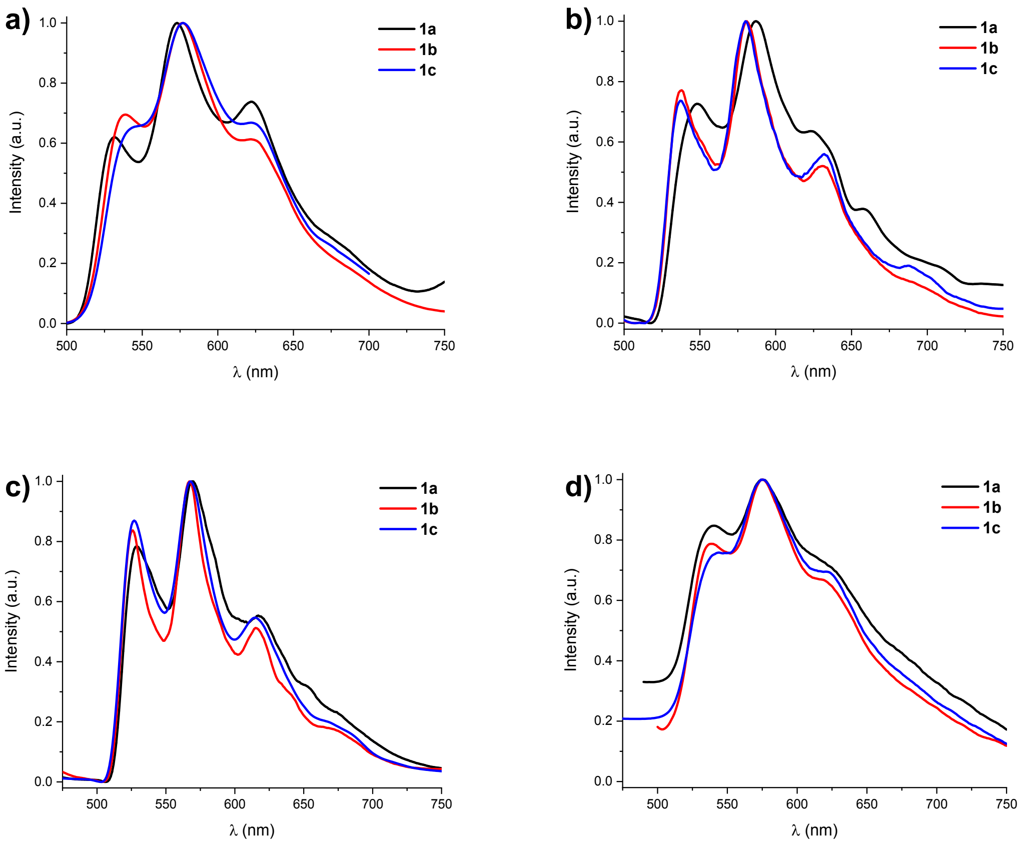

Figure S17. Emission spectra of complexes **1** in a) solid state at 298 K, b) solid state at 77K, c) THF solution (5×10^{-4} M) at 77 K and d) PS film (10% *wt*).

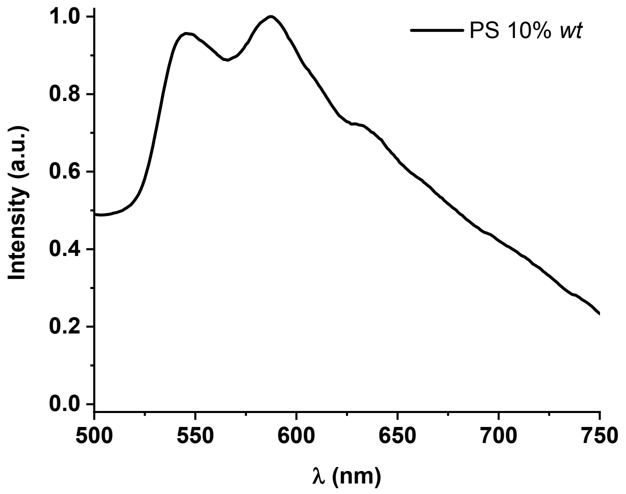

Figure S18. Emission spectra of complex **2a** in doped PS (10% *wt*).

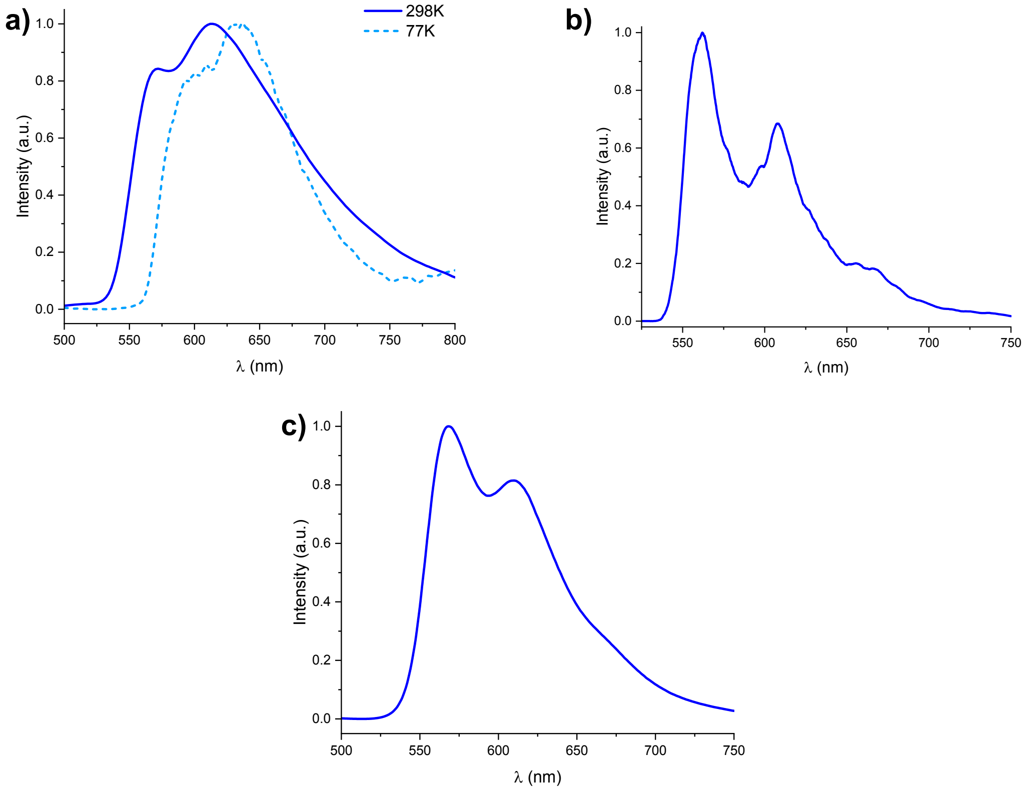

Figure S19. Emission spectra of complex **3a** in a) solid state at 298 K (solid line) and at 77K (dash line), b) THF solution at 77 K and c) PS film (10% *wt*).

Table S9. Plots and composition (%) of the frontier MOs and spin density of the first triplet state in solution for **1a**$^+$, **2a** and **3a**.

**1a**$^+$		
SOMO	SOMO-1	Spin density
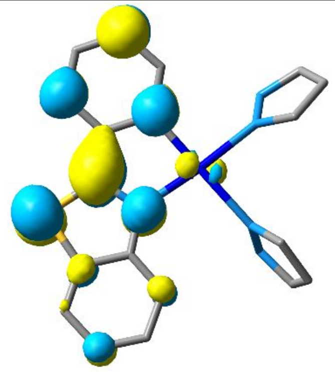	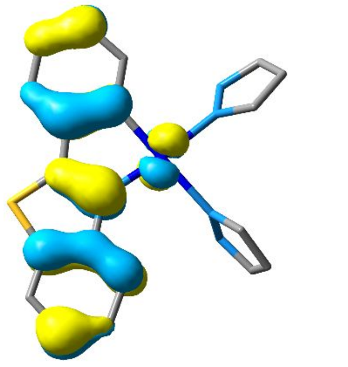	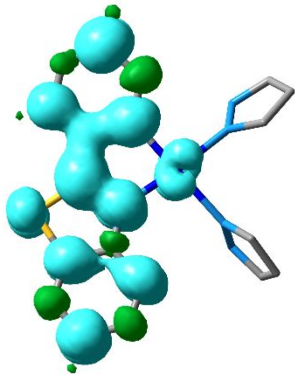
-4.24 eV<br>Pt 5 %, pbt 94 %, pzH(1) 1 %, pzH(2) 0 %	-4.88 eV<br>Pt 10 %, pbt 90 %, pzH(1) 0 %, pzH(2) 0 %	0.1303 on **Pt**
**2a**		
SOMO	SOMO-1	Spin density
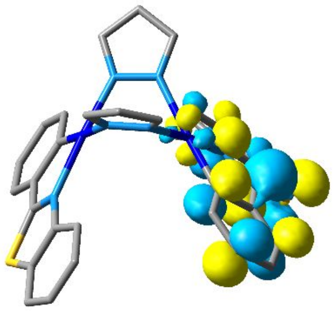	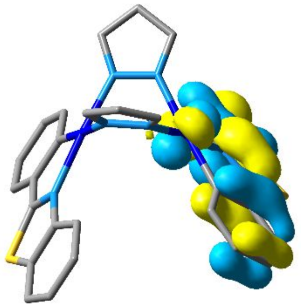	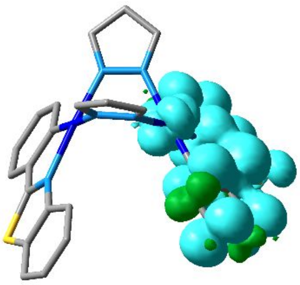
-3.71eV<br>Pt(1) 0 %, Me_2N-pbt(1) 0 %, pz(1) 0 %, Pt(2) 5 %, Me_2N-pbt(2) 94 %, pz(2) 0 %	-4.24 eV<br>Pt(1) 0 %, Me_2N-pbt(1) 0 %, pz(1) 0 %, Pt(2) 17 %, Me_2N-pbt(2) 80 %, pz(2) 2 %	0.0082 on **Pt(1)** and 0.2043 on **Pt(2)**
**3a**		
SOMO	SOMO-1	Spin density
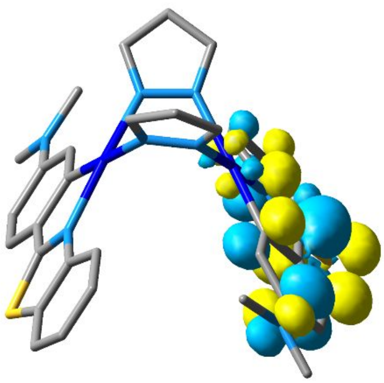	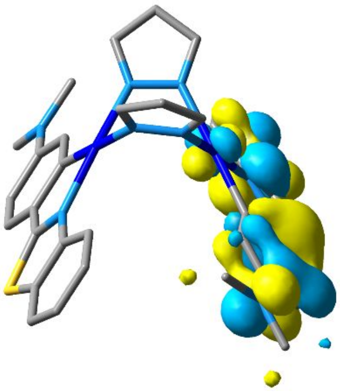	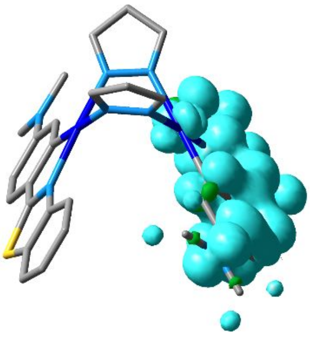
-3.04 eV<br>Pt(1) 0 %, pbt(1) 0 %, pz(1) 0 %, Pt(2) 4 %, pbt(2) 95 %, pz(2) 0 %	-3.84 eV<br>Pt(1) 0 %, pbt(1) 0 %, pz(1) 0 %, Pt(2) 6 %, pbt(2) 94 %, pz(2) 0 %	0.0011 on **Pt(1)** and 0.0798 on **Pt(2)**

Table S10. Calculated emission energies (λ_{em} /nm) in solution.

Complex	Emission energy calculated (nm)
**1a$^+$**	638
**2a**	675
**3a**	629

Singlet Oxygen Measurements

The singlet oxygen quantum yield was measured evaluating the phosphorescence of $^1O_2$ at 1274 nm with an Ediumburgh FLS1000 spectrofluorimeter equipped with Xe lamp and a NIR detector (900 – 1800 nm). The absorbance was detected with a Hewlett Packard 8453 spectrophotometer. For each sample, the emission spectra were recorded upon excitation at 365 nm. As a reference the whole procedure was repeated for the known standard phenalenone (PN). The area below the signal was integrated with Origin Pro2018. The respective singlet oxygen quantum yield $\phi(^1O_2)$ was calculated and referenced against the literature reported value for the phenalenone $\phi = 1$ in acetonitrile,[14] following the next equation:

$$\Phi_C = \Phi_R \cdot \frac{A_R}{A_C} \cdot \frac{I_C}{I_R} \quad \text{(eq. 1)}$$

In this equation ϕ_C is the $^1O_2$ quantum yield of the compound and ϕ_R is the $^1O_2$ quantum yield of the reference (phenalenone), A_C the absorbance of the compound and A_R the absorbance of reference, I_C the area of the singlet oxygen emission of the compound and I_R the area of the singlet oxygen emission of the reference.

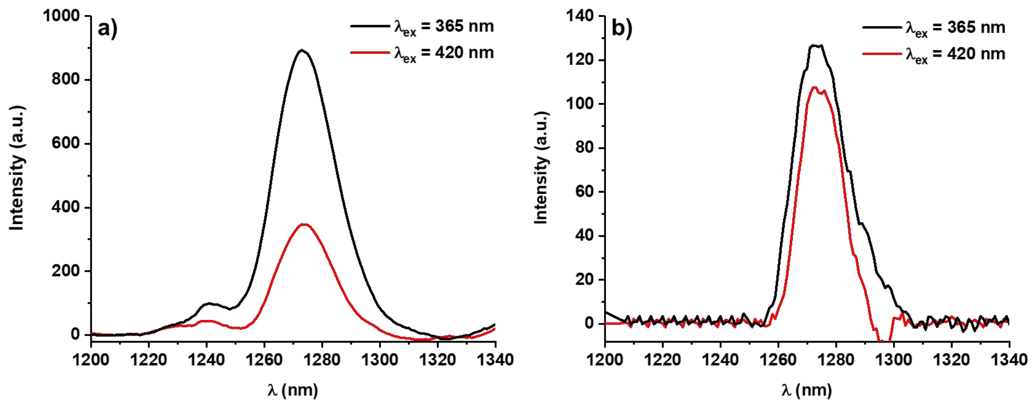

Figure S21. Emission band of the singlet oxygen from fresh solutions of a) phenalenone (PN) and b) complex **3a** in MeCN solution (5×10^{-5} M) (λ_{ex} 365 and 420 nm).

6.- Electrochemical Properties

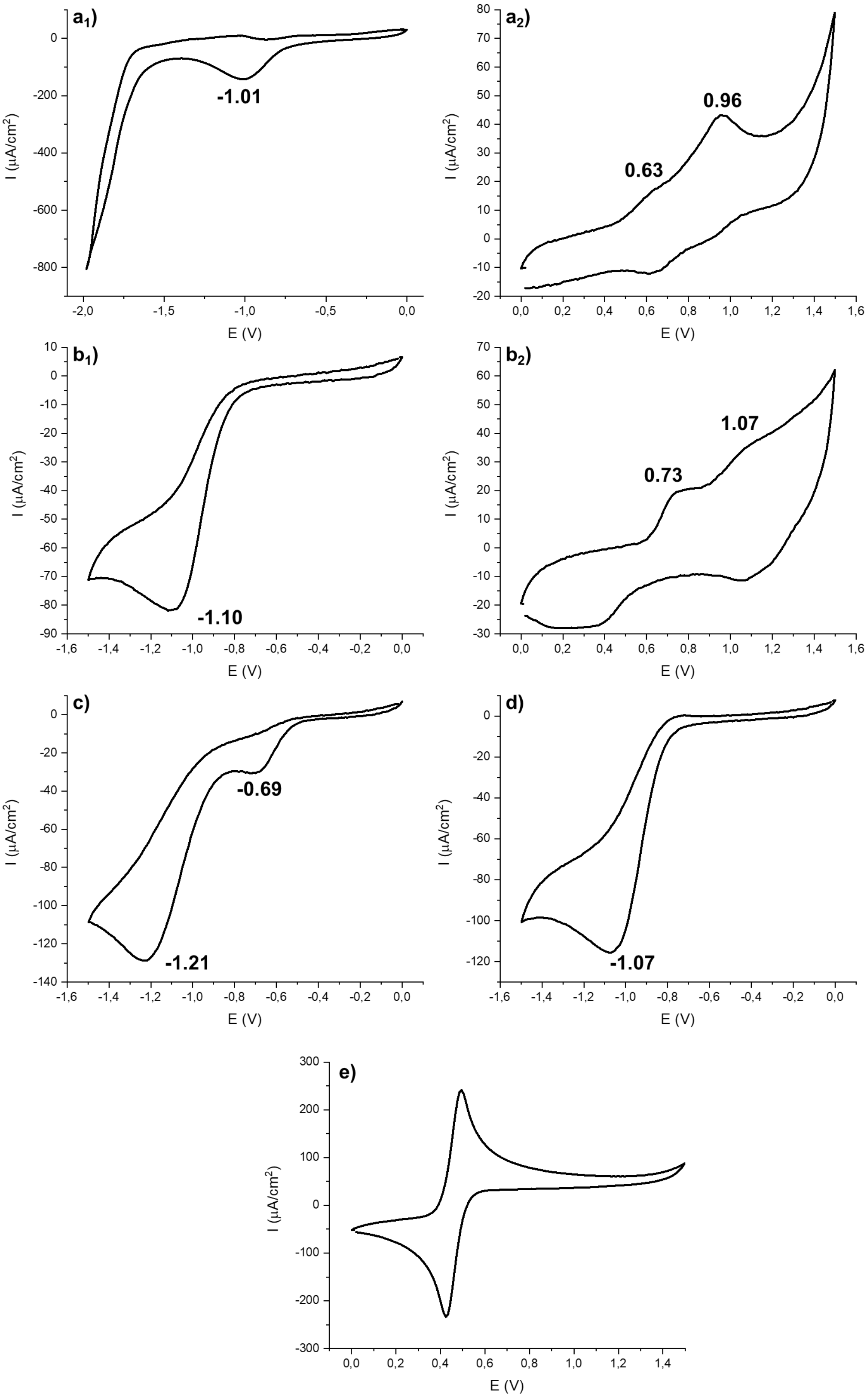

Figure S22. Cyclic Voltammograms of a) **2a** (a_1 cathodic region, a_2 anodic region); b) **3a** (b_1 cathodic region, b_2 anodic region); c) **4a**; d) **5a** (cathodic region) and e) Fc/Fc^+ couple in CH_2Cl_2 solution in the dark at 100 mVs^{-1}.

7.- Photocatalytic Studies

The photocatalytic reactions were carried out by mixing the *p*-bromothioanisole (20.3 mg, 0.1 mmol) with the corresponding amount of complex **3a** (1.03 mg, 0.001 mmol for 1% catalysis and 5.19 mg, 0.005 mmol for 5% catalysis) in CD_3OD (700 μL) and irradiating with blue light (λ = 460 nm) of an RGB LED of 50 W.

Table S11. Conversion (%) of heterogeneous visible-light oxidative reactions measured every 3 h.

Time (h)	% Photosensitizer		
	1%	5%	5%[a]
**0**	0	0	0
**3**	15.3	18.7	19.8
**6**	21.9	33.8	34.2
**9**	30.1	40.6	46.8
**12**	38.3	48.2	53.5
**15**	45.1	54.8	63.1
**18**	48.6	61.3	70.3
**21**	55.8	67.9	77.8
**24**	63.1	71.6	82.7
**27**	69.4	76.7	86.7
**30**	75.4	81.5	90.8
**33**	81.1	85.1	93.2
**36**	84.7	88.4	95.2
**38**	89.4	91.2	97.0
**41**	92.6	93.8	97.9
**44**	95.6	96.5	98.5
**47**	97.5	97.7	99.2
**50**	98.5	98.8	99.5

[a]Double amount of reagent (*p*-bromothioanisole) and photosensitizer

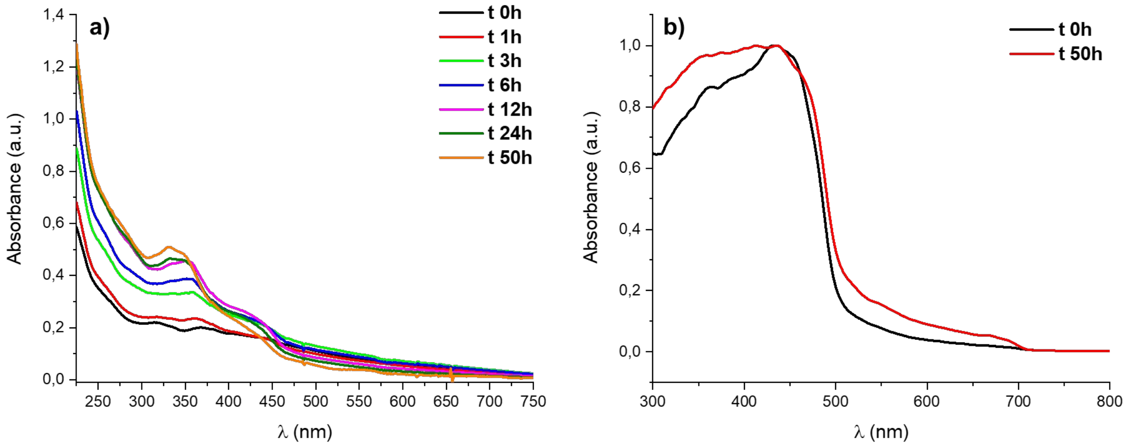

Figure S23. Photostability monitored by UV-Vis absorption spectra of a) a suspension of photosensitizer in MeOH and b) in solid state irradiated at several times (0 to 50 h) with blue light (λ = 460 nm) of an RGB LED of 50 W.

References:

(1) Gómez de Segura, D.; Lara, R.; Martínez-Junquera, M.; Lalinde, E.; Moreno, M. T. Luminescent 2-phenylbenzothiazole cyclometalated Pt^{II} and Ir^{III} complexes with chelating P^O ligands. *Dalton Trans.* **2022**, *51*, 274.

(2) Lalinde, E.; Moreno, M. T.; Lara, R.; López, I. P.; Alfaro-Arnedo, E.; Pichel, J. G.; Piñeiro-Hermida, S. Benzothiazole Based Cycloplatinated Chromophores: Synthesis, Optical and Biological Studies. *Chem. Eur. J.* **2018**, *24*, 2440.

(3) Sheldrick, G. M. SHELXT – Integrated space-group and crystal structure determination. *Acta Crystallogr., Sect. A: Found. Crystallogr.* **2015**, *71*, 3.

(4) Farrugia, L. J. WinGX suite for small-molecule single-crystal crystallography. *Appl. Crystallogr.* **1999**, *32*, 837.

(5) Sheldrick, G. Crystal structure refinement with SHELXL. *Acta Crystallogr., Sect. C.* **2015**, *71*, 3.

(6) Speck, A. L. Single-crystal structure validation with the program PLATON. *J. Appl. Cryst.* **2003**, *36*, 7.

(7) Speck, A. L. PLATON SQUEEZE: a tool for the calculation of the disordered solvent contribution to the calculated structure factors. *Acta Crystallogr., Sect C.* **2015**, *71*, 9.

(8) Frisch, M. J.; Trucks, G. W.; Schlegel, H. B.; Scuseria, G. E.; Robb, M. A.; Cheeseman, J. R.; Scalmani, G.; Barone, V.; Petersson, G. A.; Nakatsuji, H.; Li, X.; Caricato, M.; Marenich, A. V.; Bloino, J.; Janesko, B. G.; Gomperts, R.; Mennucci, B.; Hratchian, H. P.; Ortiz, J. V.; Izmaylov, A. F.; Sonnenberg, J. L.; Williams-Young, D.; Ding, F.; Lipparini, F.; Egidi, F.; Goings, J.; Peng, B.; Petrone, A.; Henderson, T.; Ranasinghe, D.; Zakrzewski, V. G.; Gao, J.; Rega, N.; Zheng, G.; Liang, W.; Hada, M.; Ehara, M.; Toyota, K.; Fukuda, R.; Hasegawa, J.; Ishida, M.; Nakajima, T.; Honda, Y.; Kitao, O.; Nakai, H.; Vreven, T.; Throssell, K.; Montgomery, J. A.; Jr., J. E. P.; Ogliaro, F.; Bearpark, M. J.; Heyd, J. J.; Brothers, E. N.; Kudin, K. N.; Staroverov, V. N.; Keith, T. A.; Kobayashi, R.; Normand, J.; Raghavachari, K.; Rendell, A. P.; Burant, J. C.; Iyengar, S. S.; Tomasi, J.; Cossi, M.; Millam, J. M.; Klene, M.; Adamo, C.; Cammi, R.; Ochterski, J. W.; Martin, R. L.; Morokuma, K.; Farkas, O.; Foresman, J. B.; Fox, D. J. *Revision A.03, Inc., Gaussian 16, Wallingford CT, 2016*, 2016.

(9) (a) Becke, A. D. Density-functional thermochemistry. III. The role of exact exchange. *J. Chem. Phys.* **1993**, *98*, 5648. (b) Becke, A. D. Density-functional

exchange-energy approximation with correct asymptotic behavior. *Phys. Rev. A.* **1988**, *38*, 3098.

(10) Grimme, S.; Antony, J.; Ehrlich, S.; Krieg, H. A consistent and accurate ab initio parametrization of density functional dispersion correction (DFT-D) for the 94 elements H-Pu. *J. Chem. Phys.* **2010**, *132*, 154104.

(11) Wadt, W. R.; Hay, P. J. Ab initio effective core potentials for molecular calculations. Potentials for main group elements Na to Bi. *J. Chem. Phys.* **1985**, *82*, 284.

(12) Barone, V.; Cossi, M. Quantum Calculation of Molecular Energies and Energy Gradients in Solution by a Conductor Solvent Model. *J. Phys. Chem. A.* **1998**, *102*, 1995.

(13) O'Boyle, N. M.; Tenderholt, A. L.; Langner, K. M. cclib: A library for package-independent computational chemistry algorithms. *J. Comput. Chem.* **2008**, *29*, 839.

(14) Schmidt, R.; Tanielian, C.; Dunsbach, R.; Wolff, C. Phenalenone, a universal reference compound for the determination of quantum yields of singlet oxygen $O_2(^1\Delta_g)$ sensitization. *J. Photochem. Photobiol. A: Chem.* **1994**, *79*, 11.
